# Supplementary material for: Genome-wide analysis of cardiac ventricular phenotypes reveals novel loci and therapeutic targets for heart failure
Source: Nat Commun. 2026 Feb 27;17:3293. doi: 10.1038/s41467-026-69982-0 (PMC13066029; doi:10.1038/s41467-026-69982-0)
Supplement: Supplementary file 1 — Supplementary Information [file 41467_2026_69982_MOESM1_ESM.pdf]

Article Title: Genome-wide analysis of cardiac ventricular phenotypes reveals novel loci and therapeutic targets for heart failure

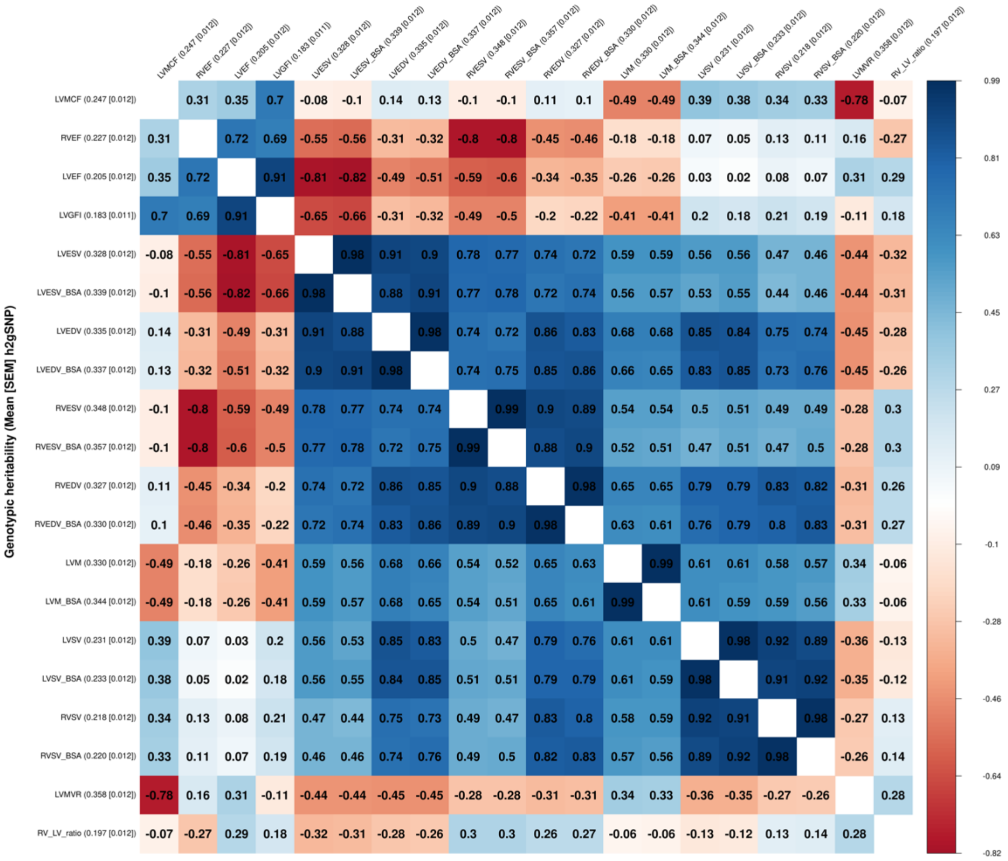

Supplementary Fig 1. Correlogram for left and right ventricular traits. Single-nucleotide polymorphism (SNP) heritability and phenotype correlation between all left and right ventricular traits. Heritability estimated from genotyped SNPs is presented along the axes per each trait. Source data are provided as a Source Data file. SNP, single nucleotide polymorphism; LVEDV, Left Ventricular End-Diastolic Volume; LVESV, Left Ventricular End-Systolic Volume; LVSV, Left Ventricular Stroke Volume; LVEF, Left Ventricular Ejection Fraction; LVGFI, Left Ventricular Global Function Index; LVMCF, Left Ventricular Myocardial Contraction Fraction; LVM, Left Ventricular Mass; LVMVR, Left Ventricular Mass-to-Volume Ratio; LVEDV BSA, Left Ventricular End-Diastolic Volume Indexed to Body Surface Area; LVESV BSA, Left Ventricular End-Systolic Volume Indexed to Body Surface Area; LVSV BSA, Left Ventricular Stroke Volume Indexed to Body Surface Area; LVM BSA, Left Ventricular Mass Indexed to Body Surface Area; RVEDV, Right Ventricular End-Diastolic Volume; RVESV, Right Ventricular End-Systolic Volume; RVSV, Right Ventricular Stroke Volume; RVEF, Right Ventricular Ejection Fraction; RV LV ratio, Right Ventricular to Left Ventricular Ratio; RVEDV BSA, Right Ventricular End-Diastolic Volume Indexed to Body Surface Area; RVESV BSA, Right Ventricular End-Systolic Volume Indexed to Body Surface Area; RVSV BSA, Right Ventricular Stroke Volume Indexed to Body Surface Area; SEM, standard error of the mean.

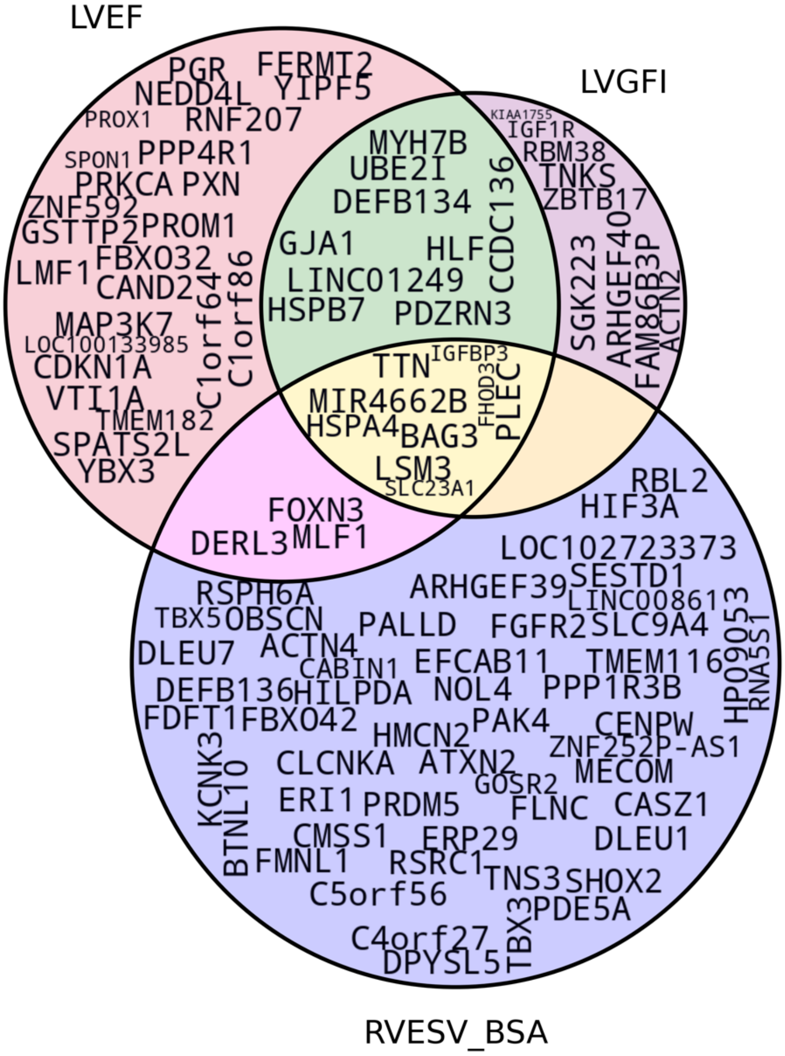

Supplementary Fig 2. Overlapping loci between the top three traits with the largest number of shared associations. Source data are provided as a Source Data file. LVEF, Left Ventricular Ejection Fraction; LVGFI, Left Ventricular Global Function Index; RVESV BSA, Right Ventricular End-Systolic Volume Indexed to Body Surface Area.

LVEDV

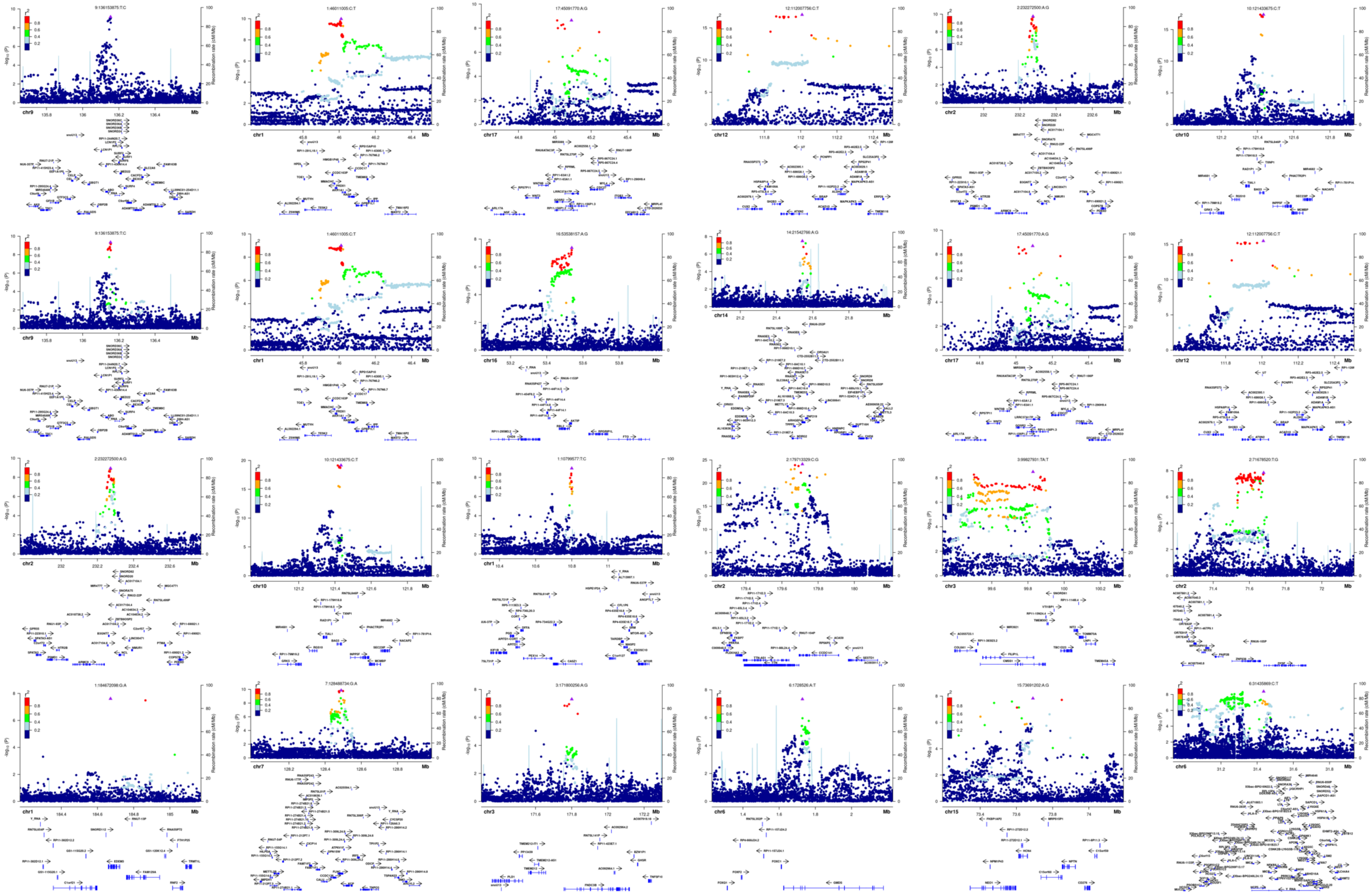
9:136153875:T:C
1:46011005:C:T
17:45091770:A:G
12:112007756:C:T
2:232272500:A:G
10:121433675:C:T
9:136153875:T:C
1:46011005:C:T
16:53538157:A:G
14:21542766:A:G
17:45091770:A:G
12:112007756:C:T
2:232272500:A:G
10:121433675:C:T
1:10799577:T:C
2:179713329:C:G
3:99827931:TA:T
2:71678520:T:G
1:184672098:G:A
7:128488734:G:A
3:171800256:A:G
6:1728526:A:T
15:73691202:A:G
6:31435869:C:T

LVEDV

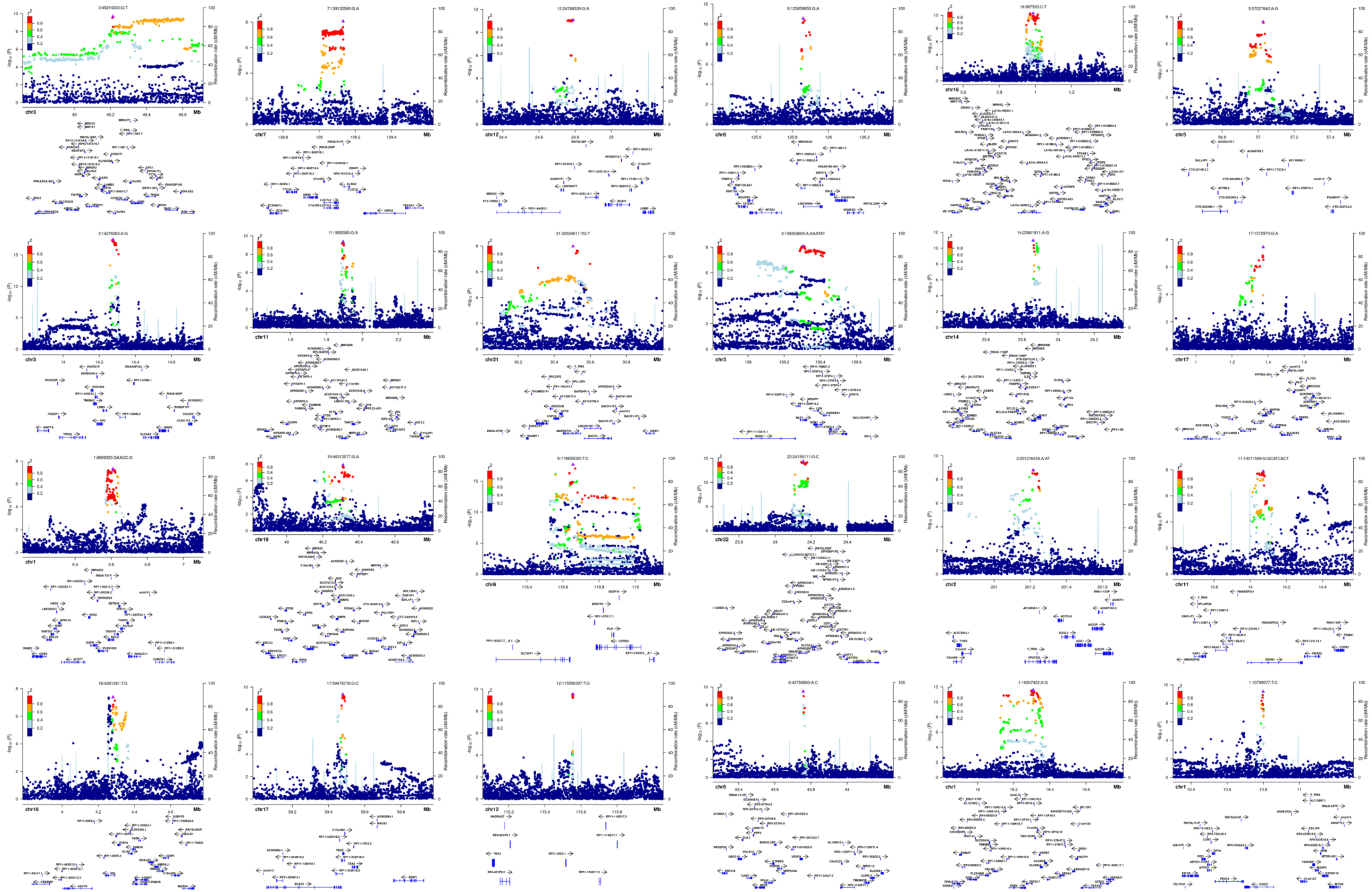

LVEDV

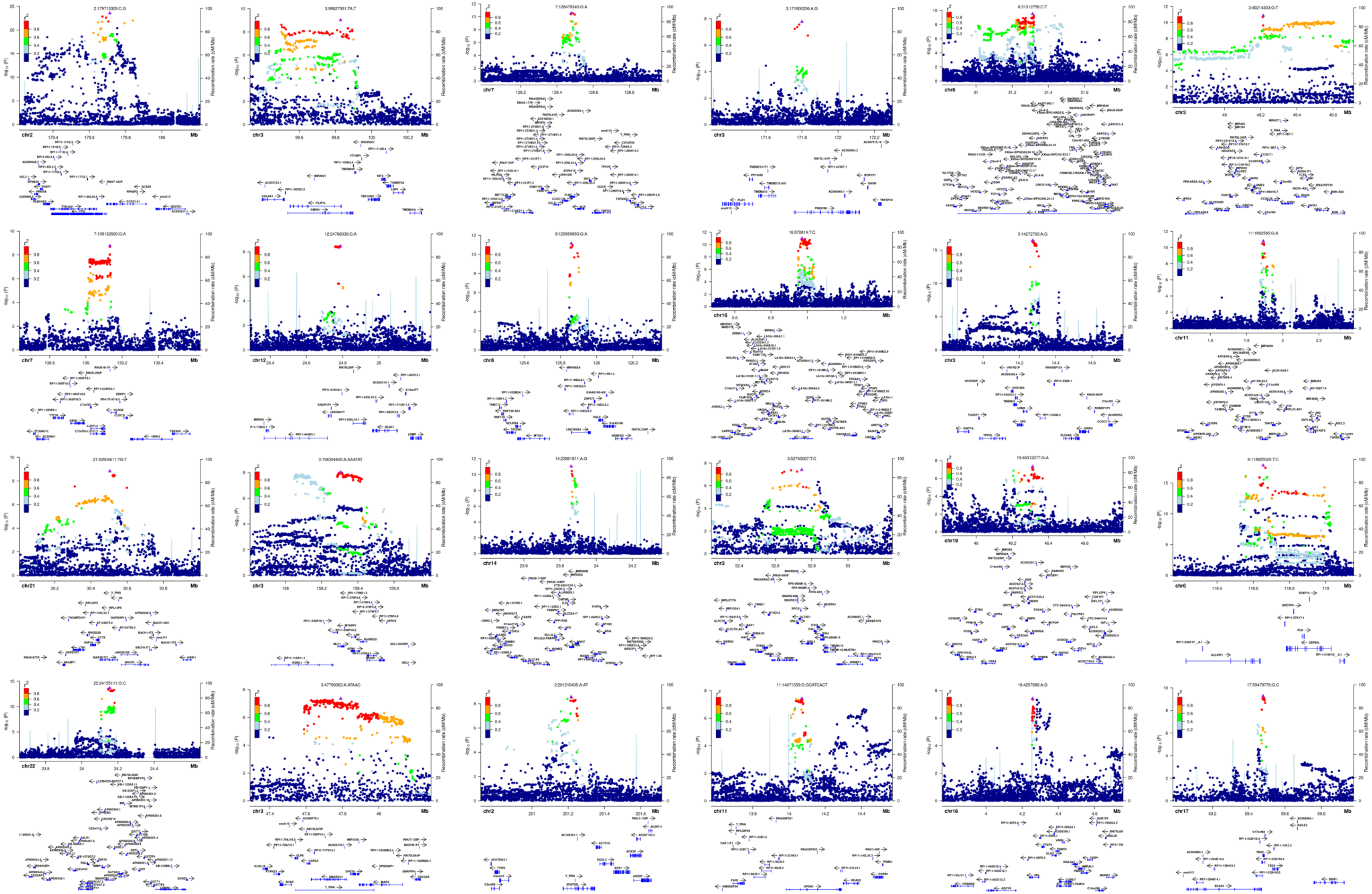

LVEDV

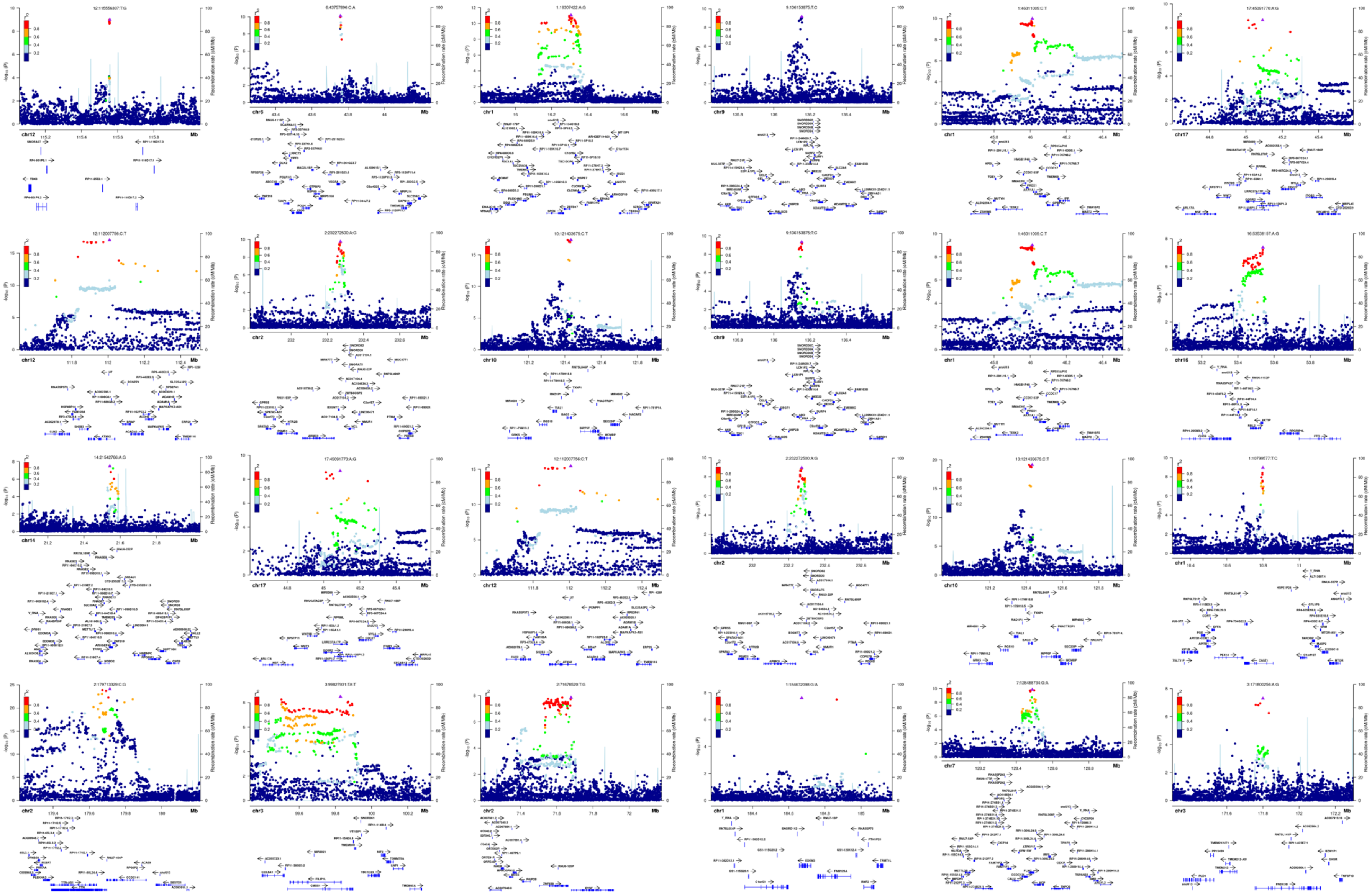

LVEDV

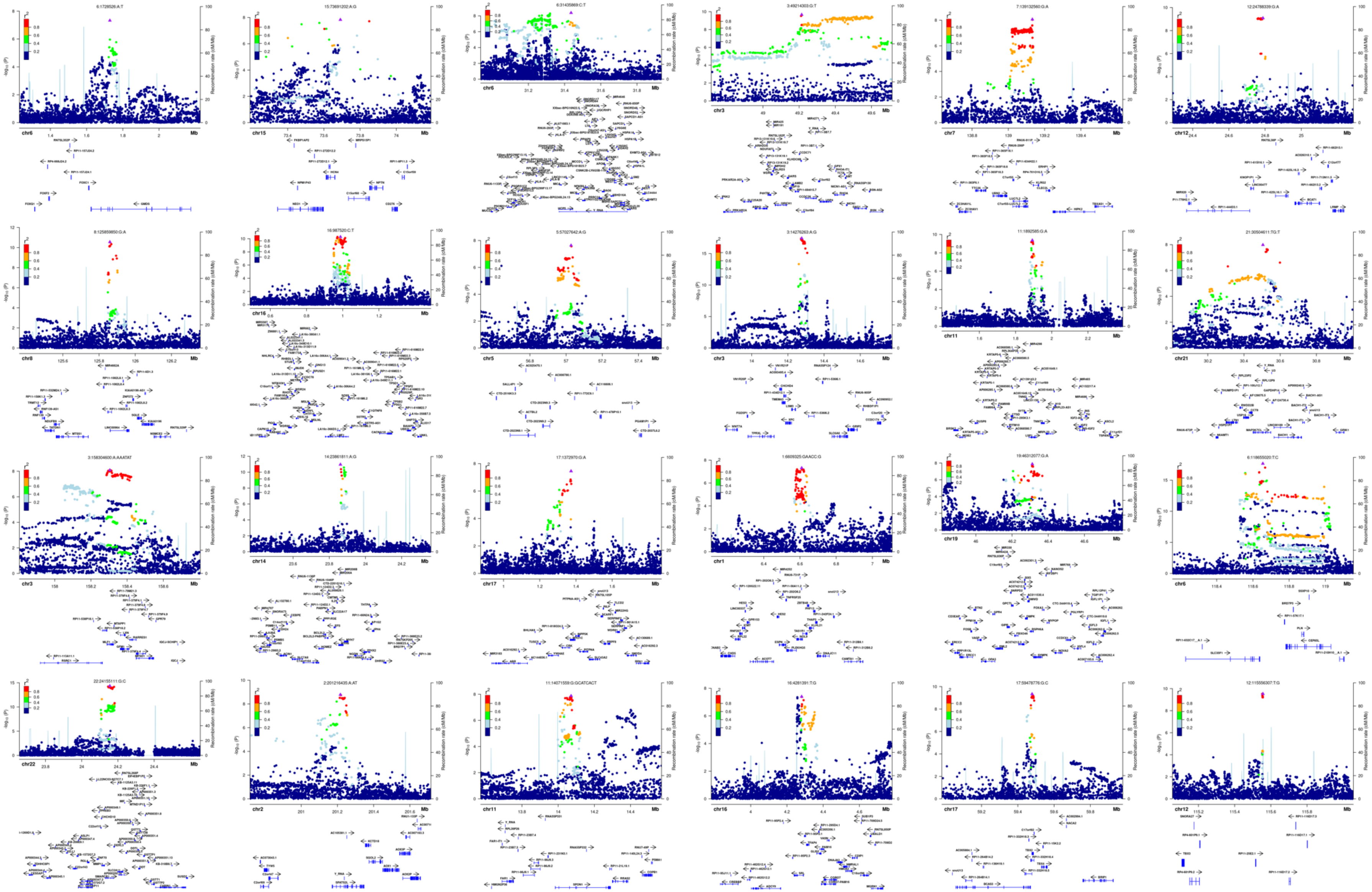
6:1728526:A:T
15:73691202:A:G
6:31435869:C:T
3:49214303:G:T
7:139132560:G:A
12:24788339:G:A
8:125859850:G:A
16:987520:C:T
5:57027642:A:G
3:14276263:A:G
11:1892585:G:A
21:30504611:TG:T
3:158304600:A:AAATAT
14:23861811:A:G
17:1372970:G:A
1:6609325:GAACC:G
19:46312077:G:A
6:118655020:T:C
22:24155111:G:C
2:201216435:A:AT
11:14071559:G:GCATCACT
16:4281391:T:G
17:59478776:G:C
12:115556307:T:G

LVEDV

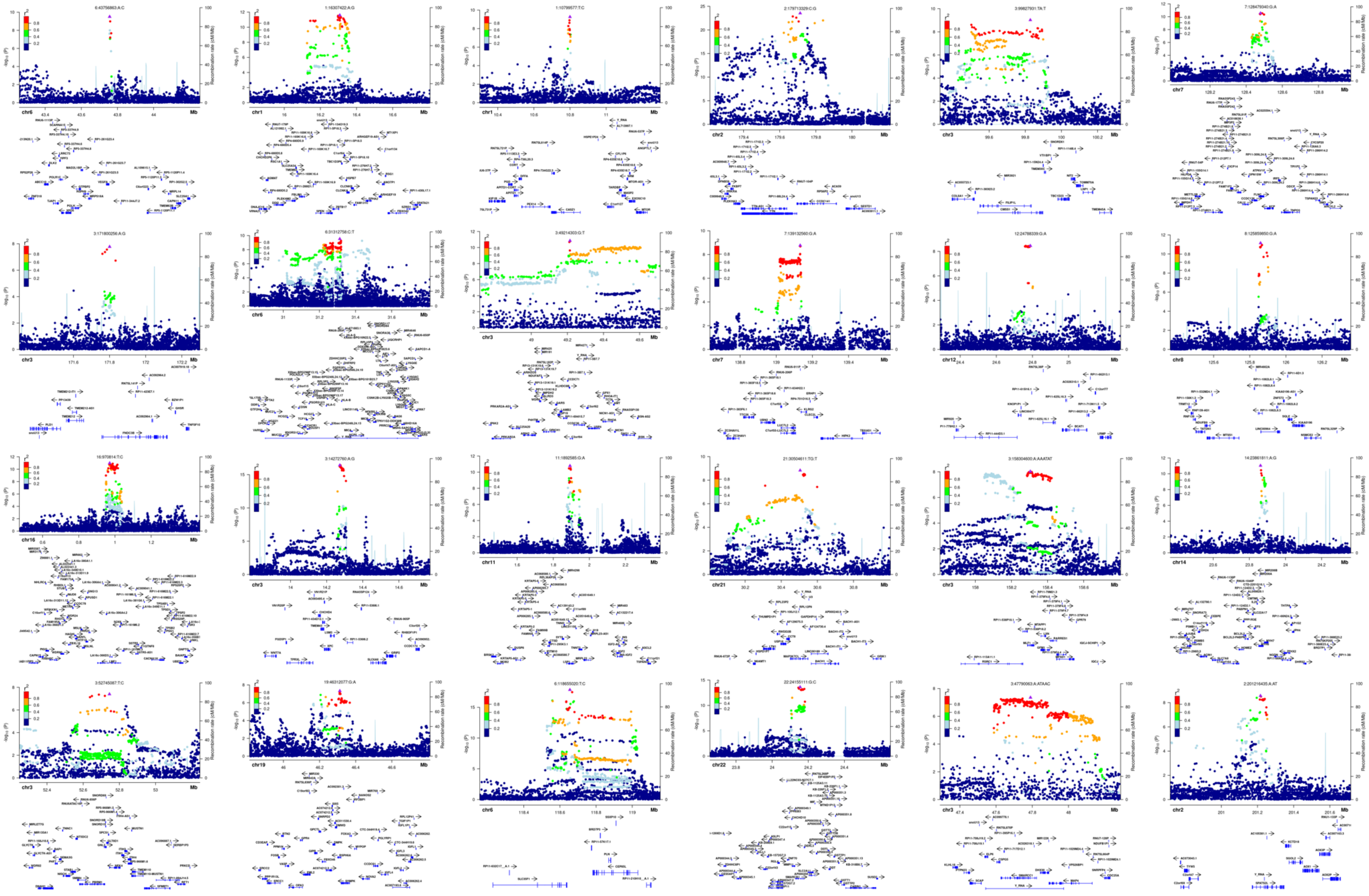

LVEDV

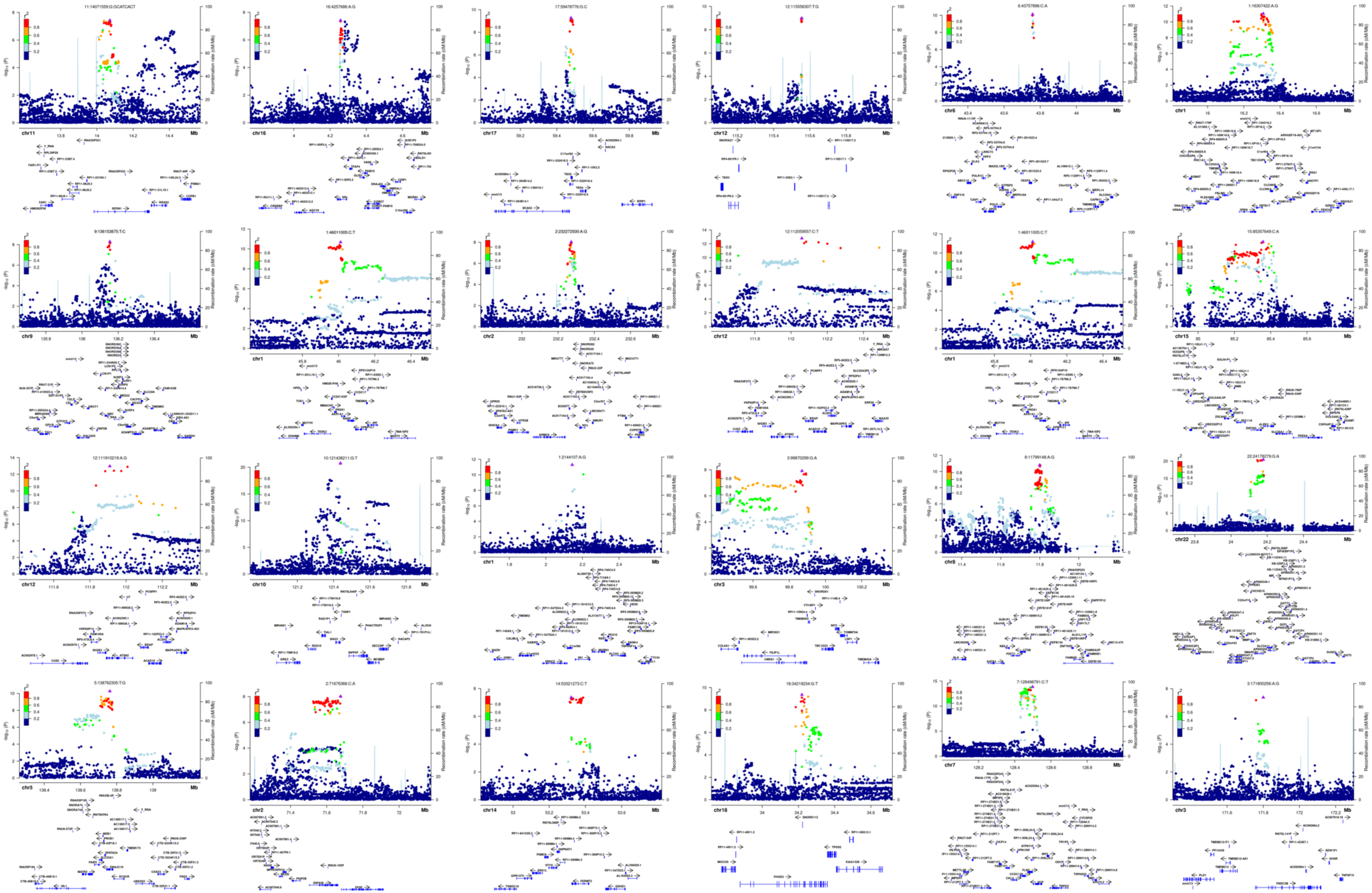

LVEDV

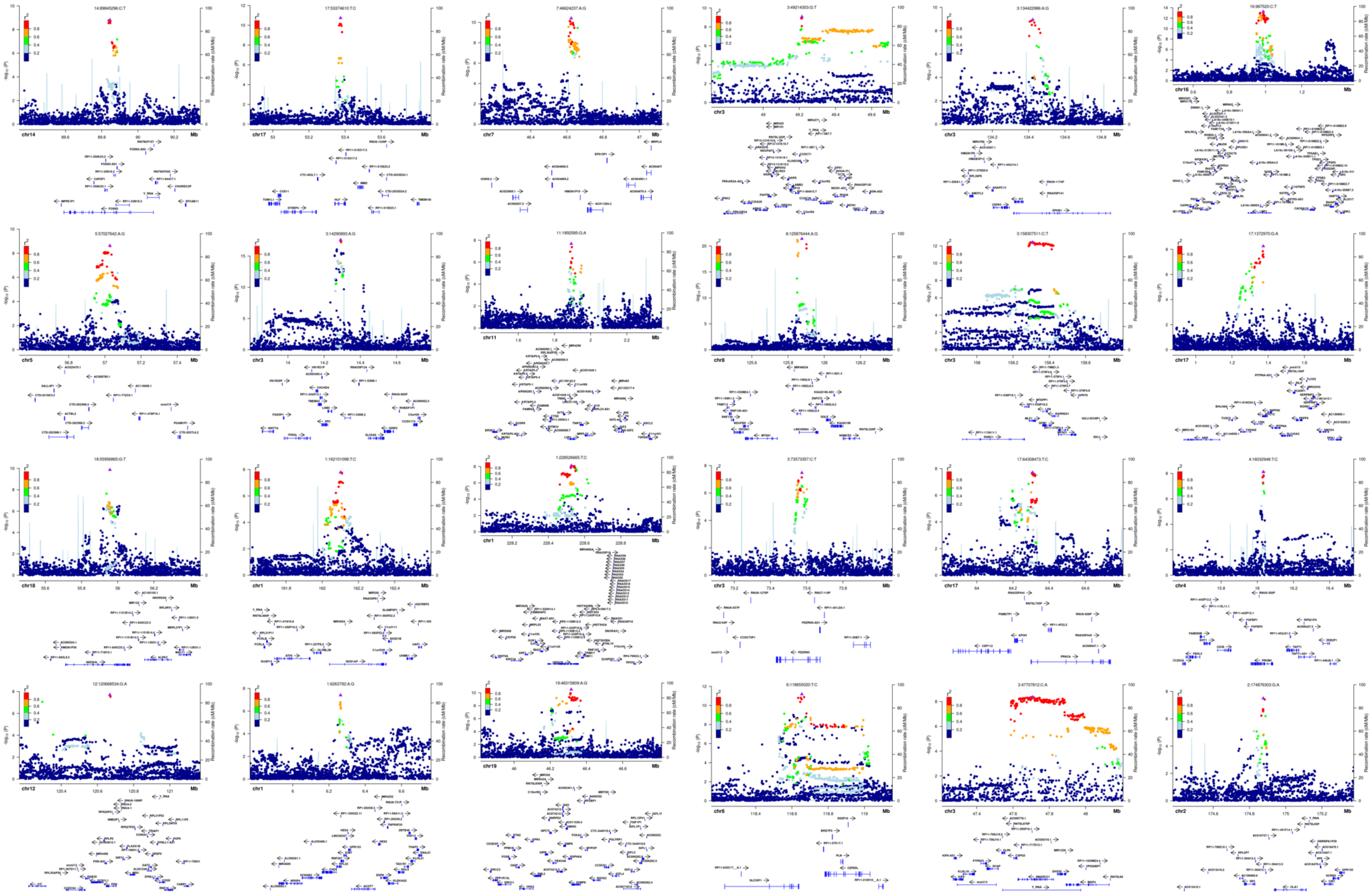

LVEDV

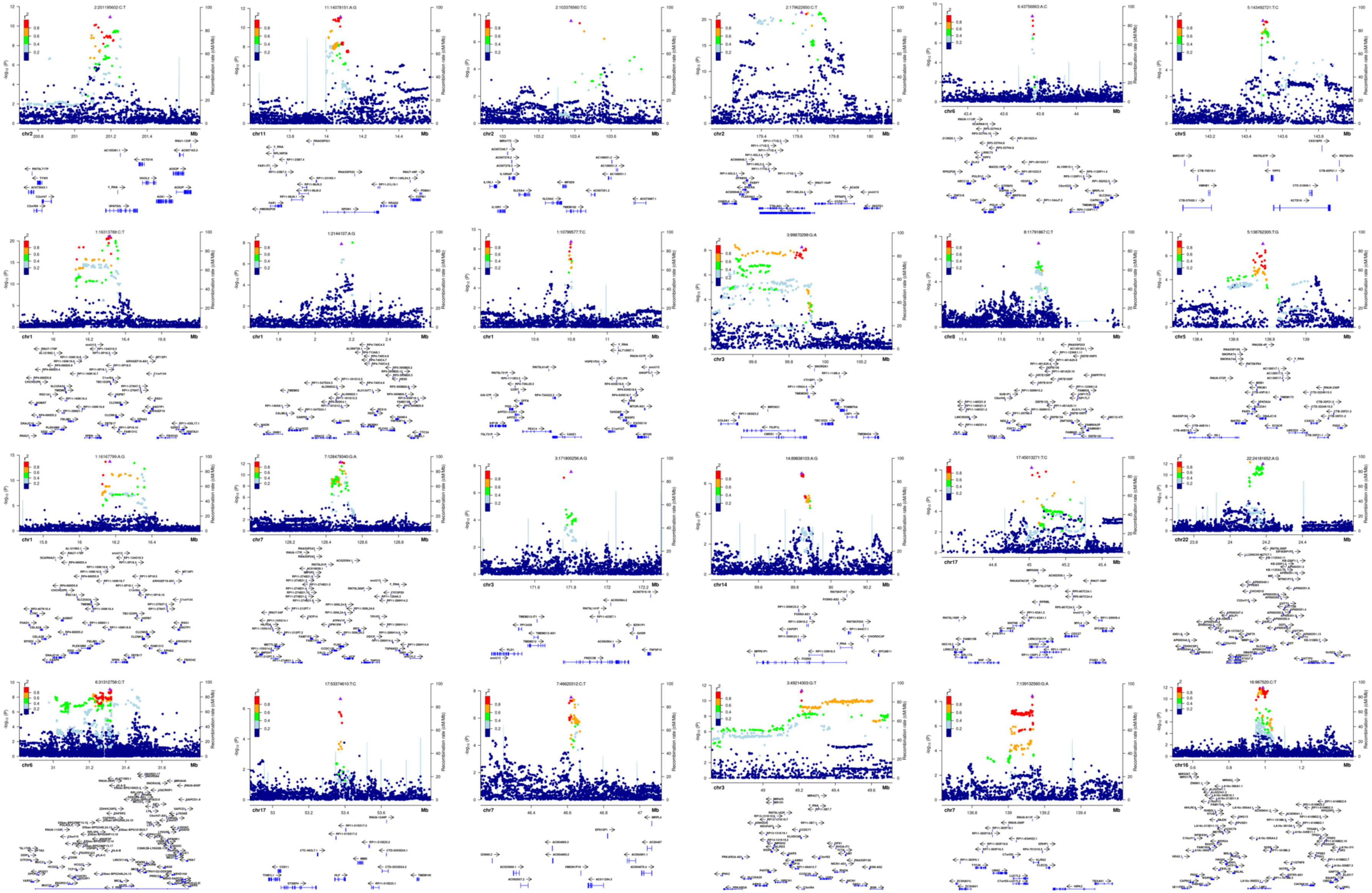
2:201195602:C:T
11:14078151:A:G
2:103376560:T:C
2:179622850:C:T
6:43756863:A:C
5:143492721:T:C
1:16313788:C:T
1:2144107:A:G
1:10799577:T:C
3:99870299:G:A
8:11791867:C:T
5:138762305:T:G
1:16167799:A:G
7:128479340:G:A
3:171800256:A:G
14:89838103:A:G
17:45013271:T:C
22:24181652:A:G
6:31312758:C:T
17:53374610:T:C
7:46620312:C:T
3:49214303:G:T
7:139132560:G:A
16:987520:C:T

LVEDV

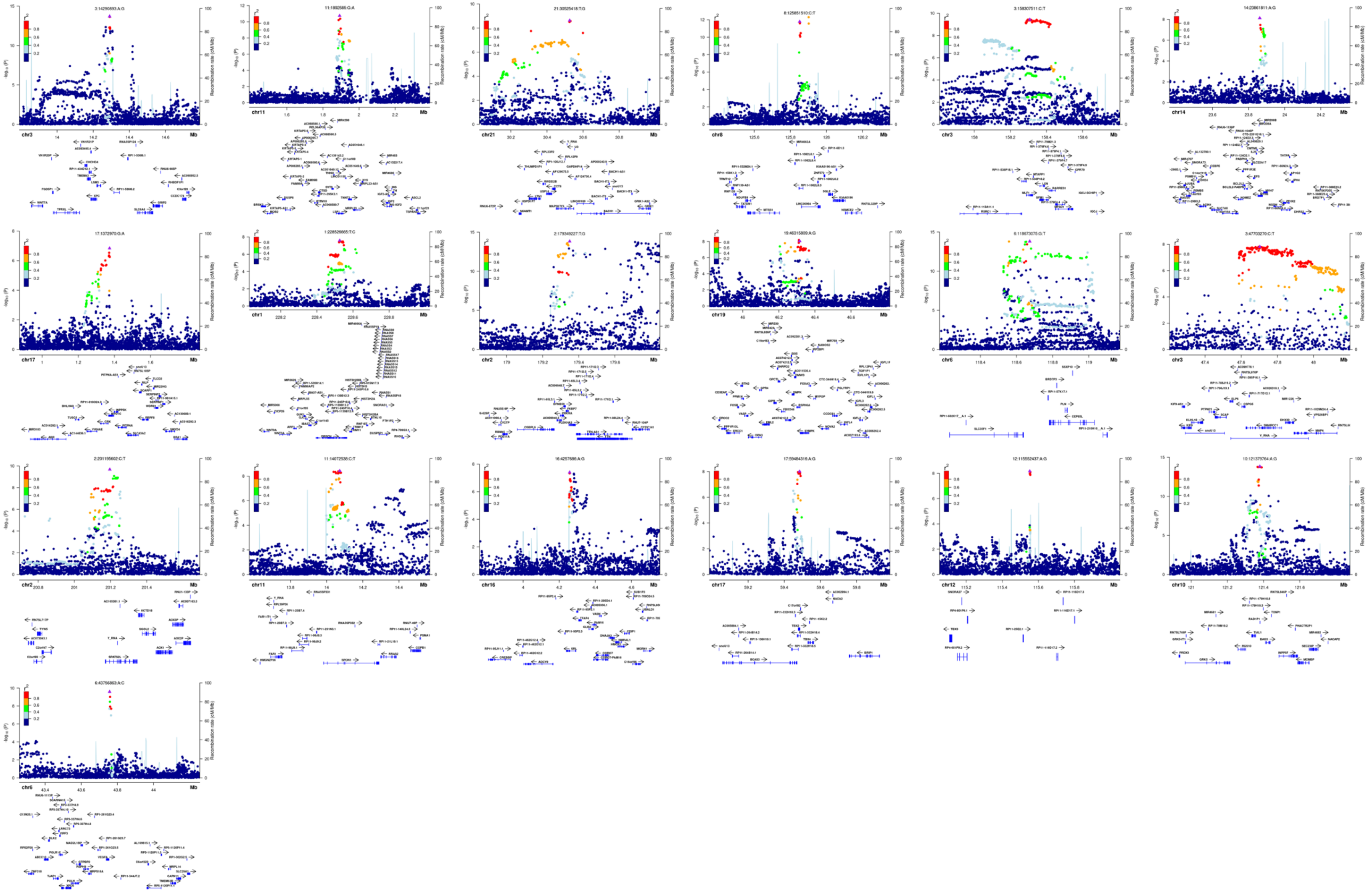

LVEDV_BSA

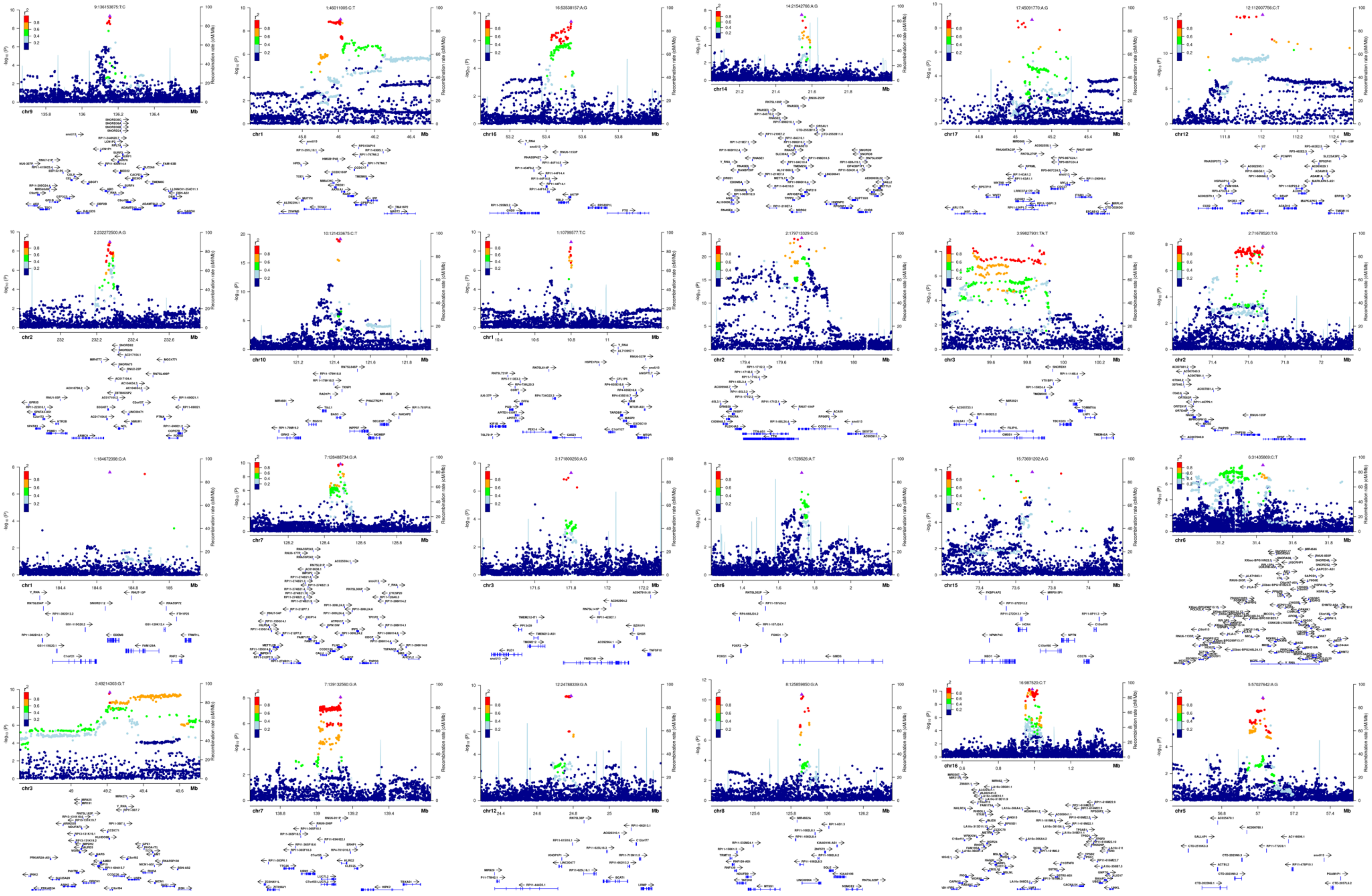

LVEDV_BSA

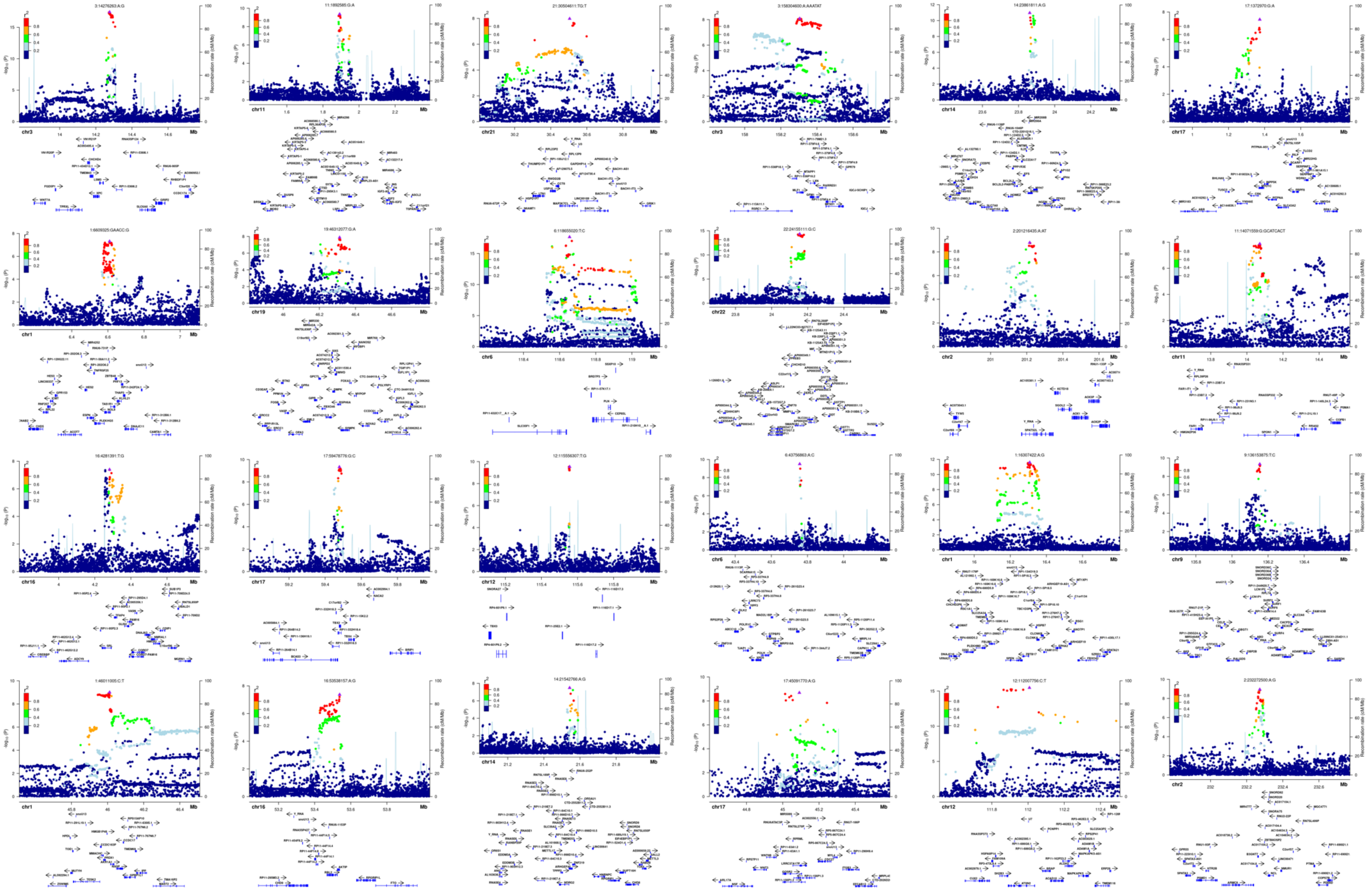
3:14276263:A:G
11:1892585:G:A
21:30504611:TG:T
3:158304600:A:AAATAT
14:23861811:A:G
17:1372970:G:A
1:6609325:GAACC:G
19:46312077:G:A
6:118655020:T:C
22:24155111:G:C
2:201216435:A:AT
11:14071559:G:GCATCACT
16:4281391:T:G
17:59478776:G:C
12:115556307:T:G
6:43756863:A:C
1:16307422:A:G
9:136153875:T:C
1:46011005:C:T
16:53538157:A:G
14:21542766:A:G
17:45091770:A:G
12:112007756:C:T
2:232272500:A:G

LVEDV_BSA

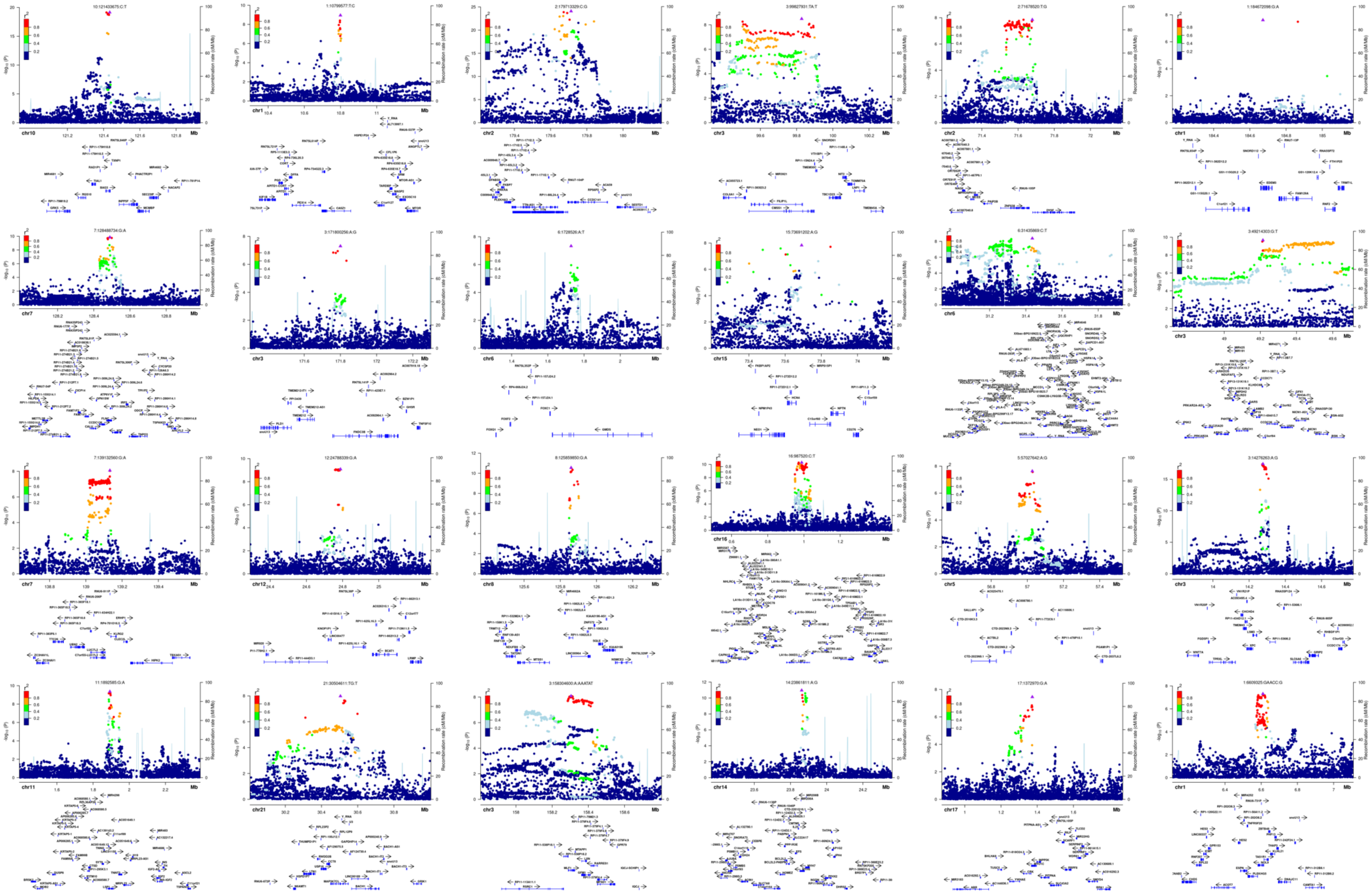
10:121433675:C:T
1:10799577:T:C
2:179713329:C:G
3:99827931:TA:T
2:71678520:T:G
1:184672098:G:A
7:128488734:G:A
3:171800256:A:G
6:1728526:A:T
15:73691202:A:G
6:31435869:C:T
3:49214303:G:T
7:139132560:G:A
12:24788339:G:A
8:125859850:G:A
16:987520:C:T
5:57027642:A:G
3:14276263:A:G
11:1892585:G:A
21:30504611:TG:T
3:158304600:A:AAATAT
14:23861811:A:G
17:1372970:G:A
1:6609325:GAACC:G

LVEDV_BSA

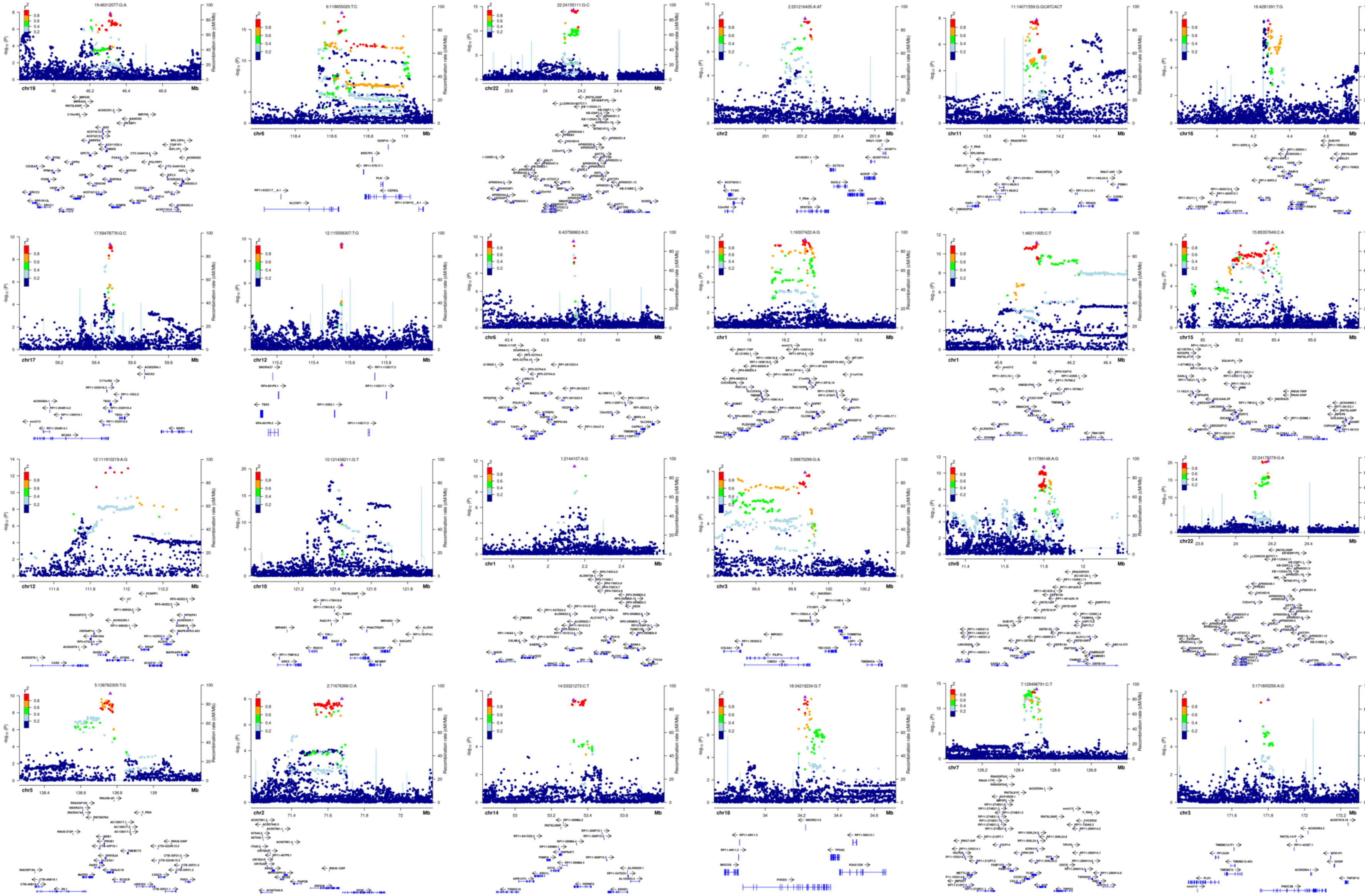

LVEDV_BSA

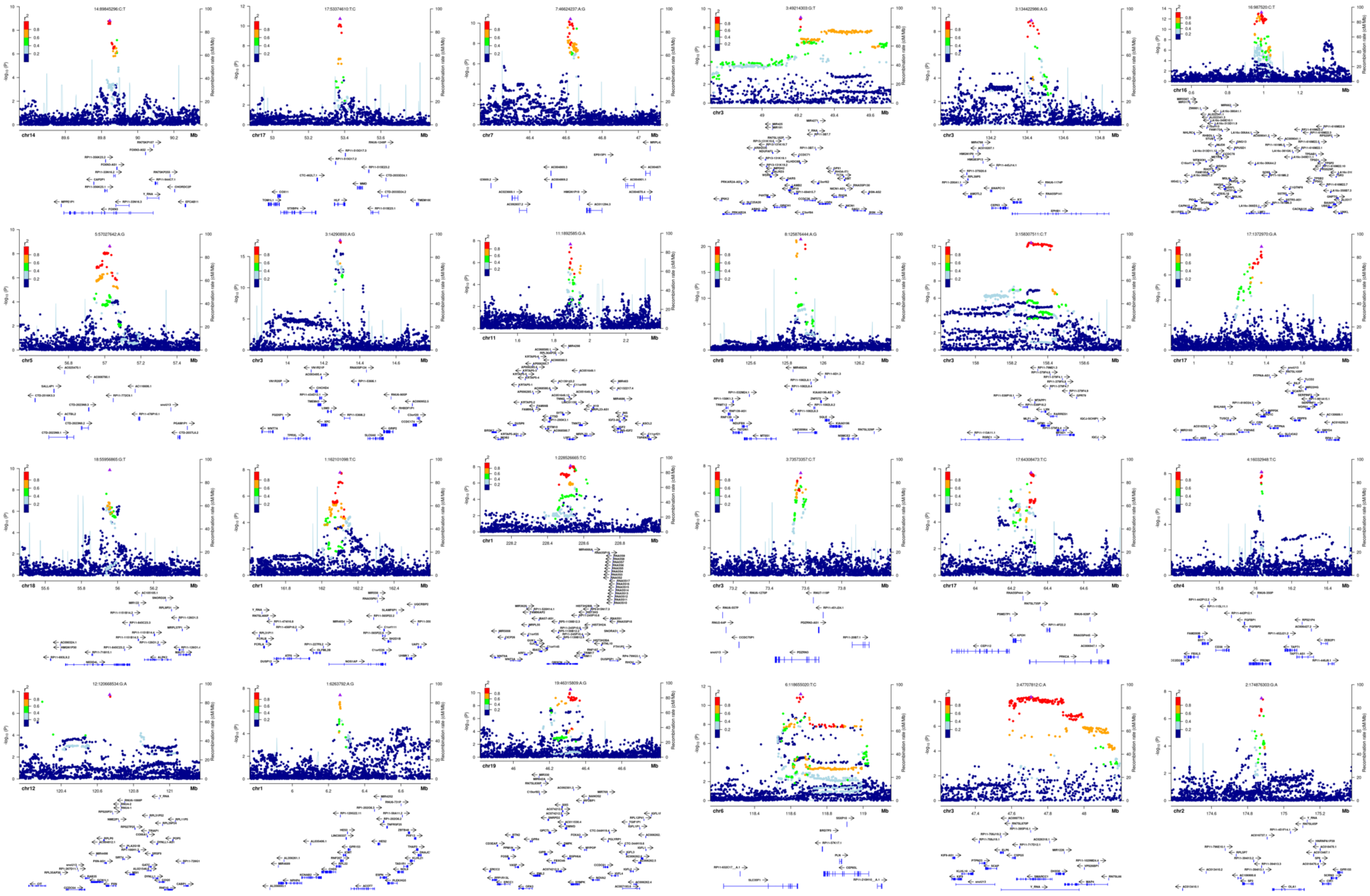

LVEDV_BSA

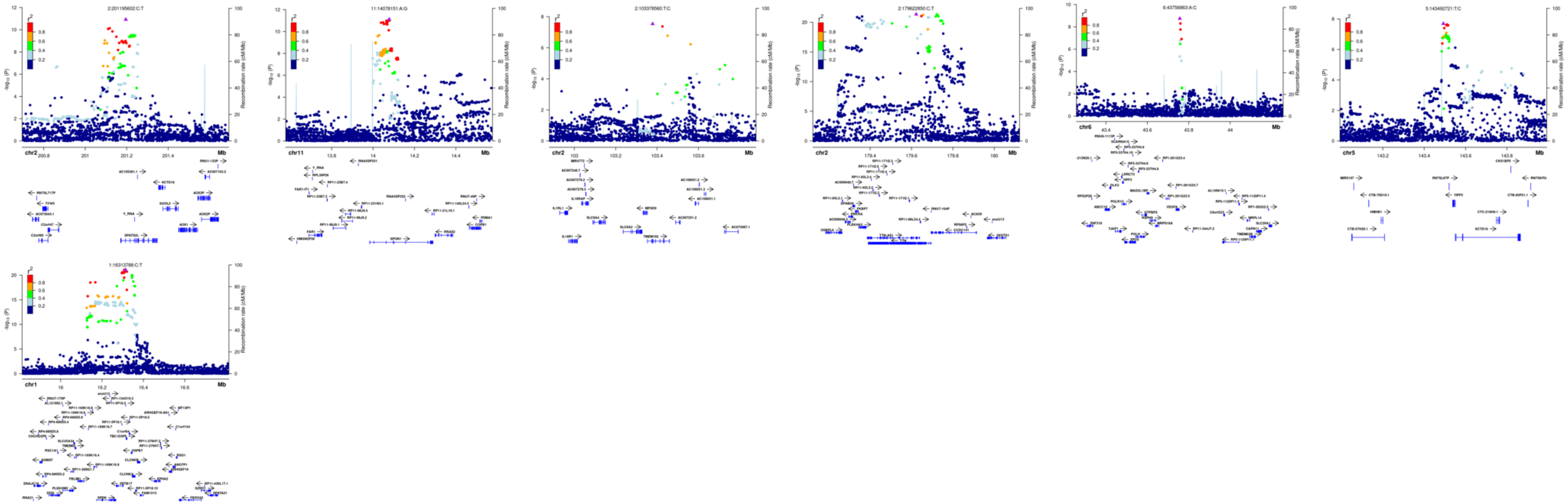
2:201195602:C:T
11:14078151:A:G
2:103378560:T:C
2:179622850:C:T
6:43756863:A:C
5:143492721:T:C
1:16313788:C:T

LVEF

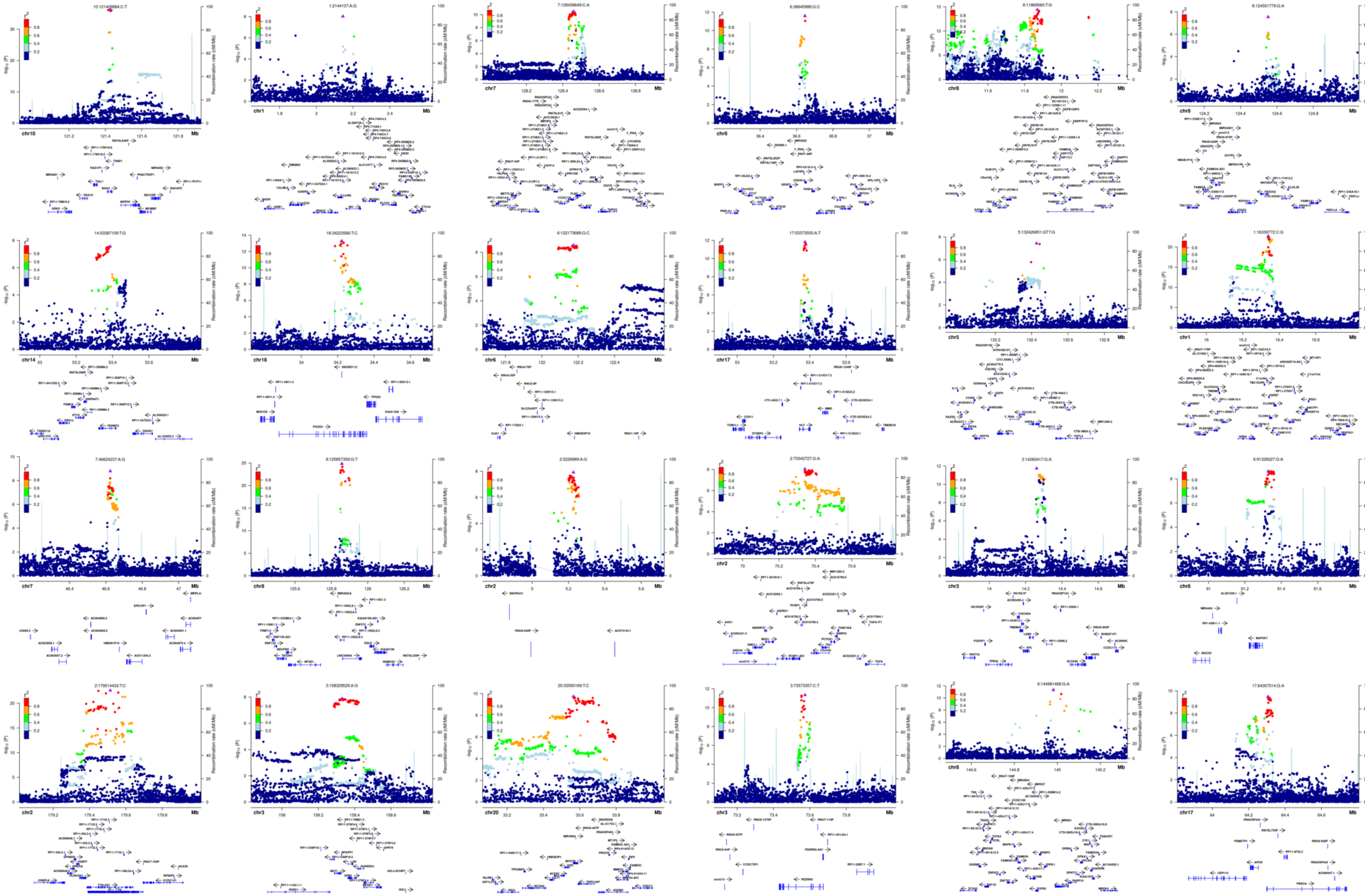

LVEF

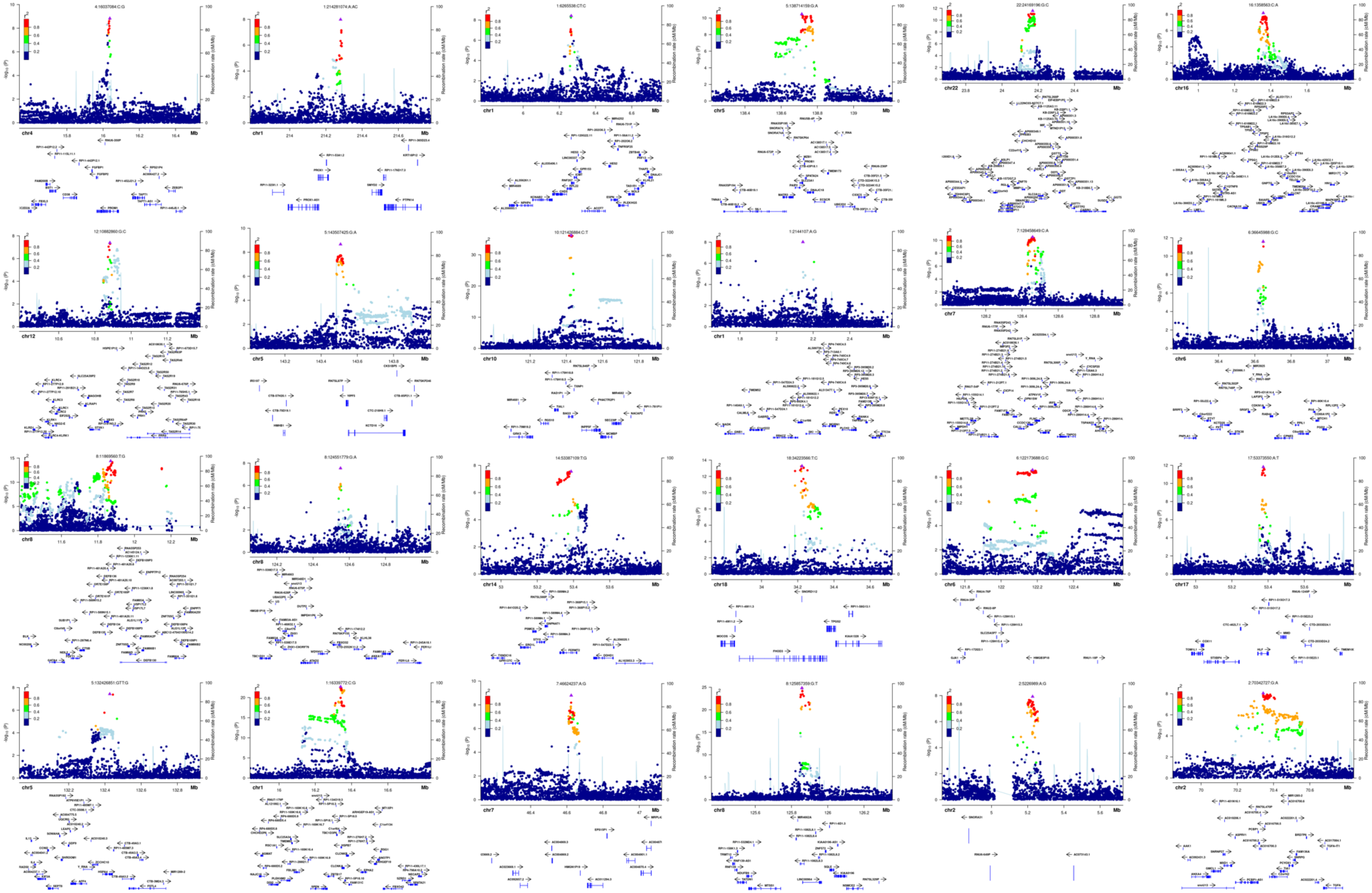

LVEF

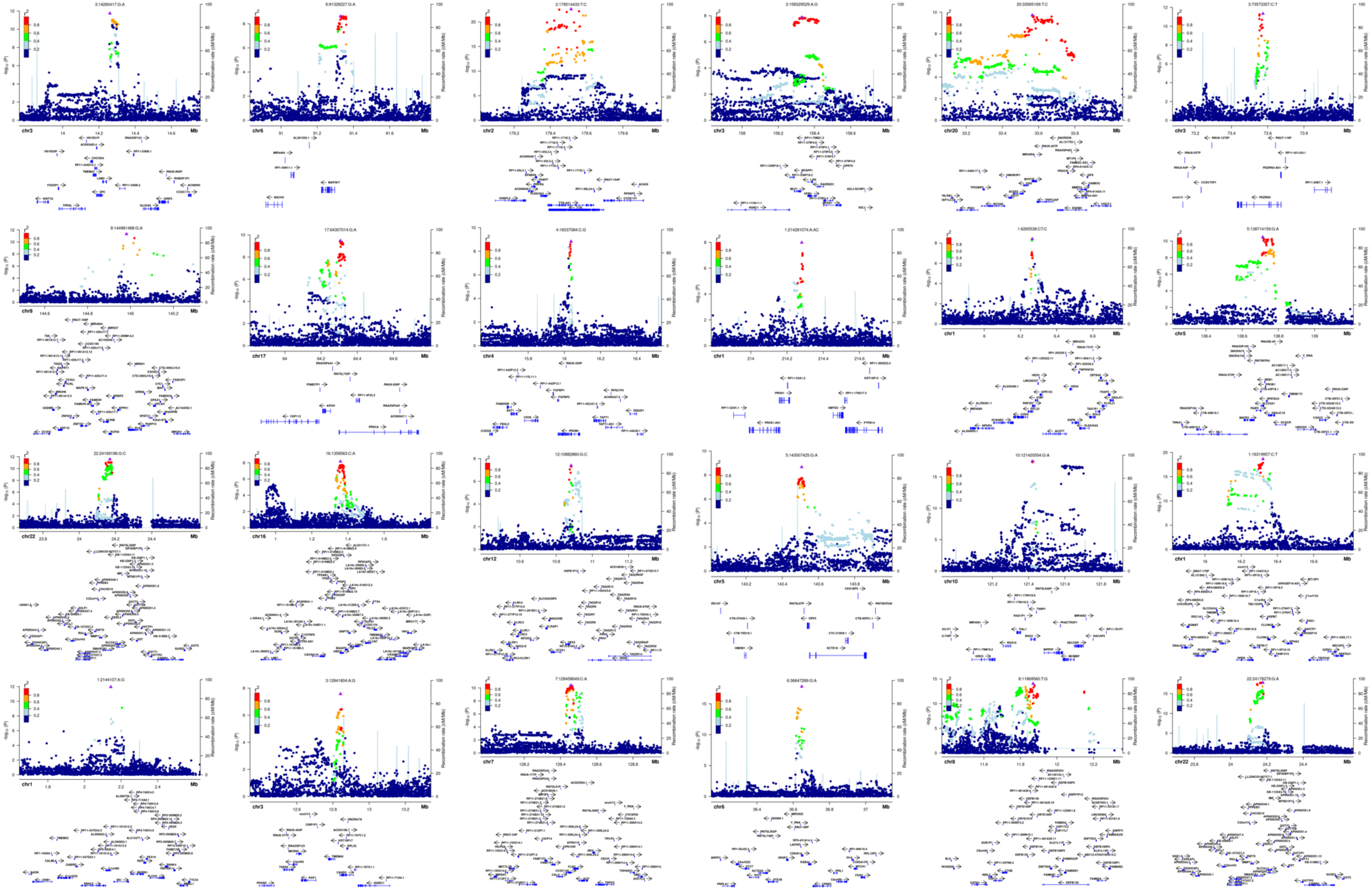

LVEF

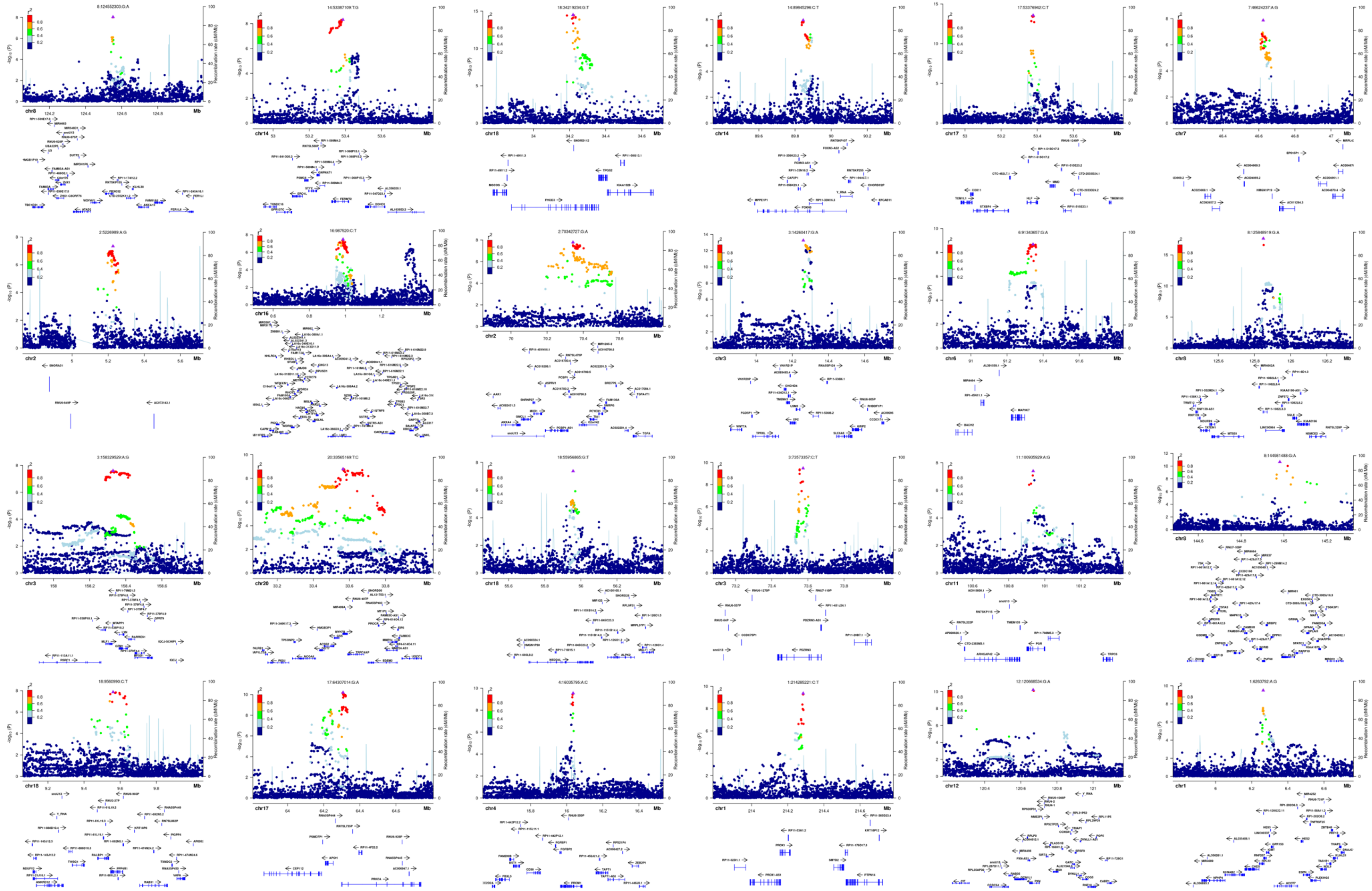

LVEF

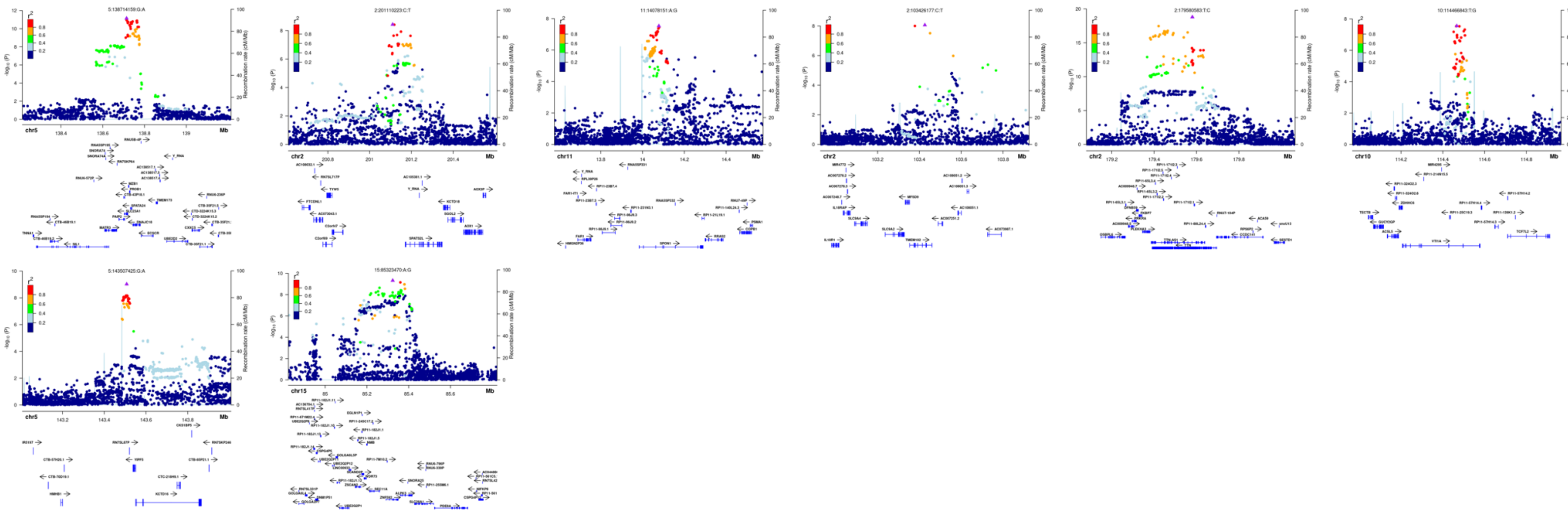
5:138714159:G:A
2:201110223:C:T
11:14078151:A:G
2:103426177:C:T
2:179580583:T:C
10:114466843:T:G
5:143507425:G:A
15:85323470:A:G

LVESV

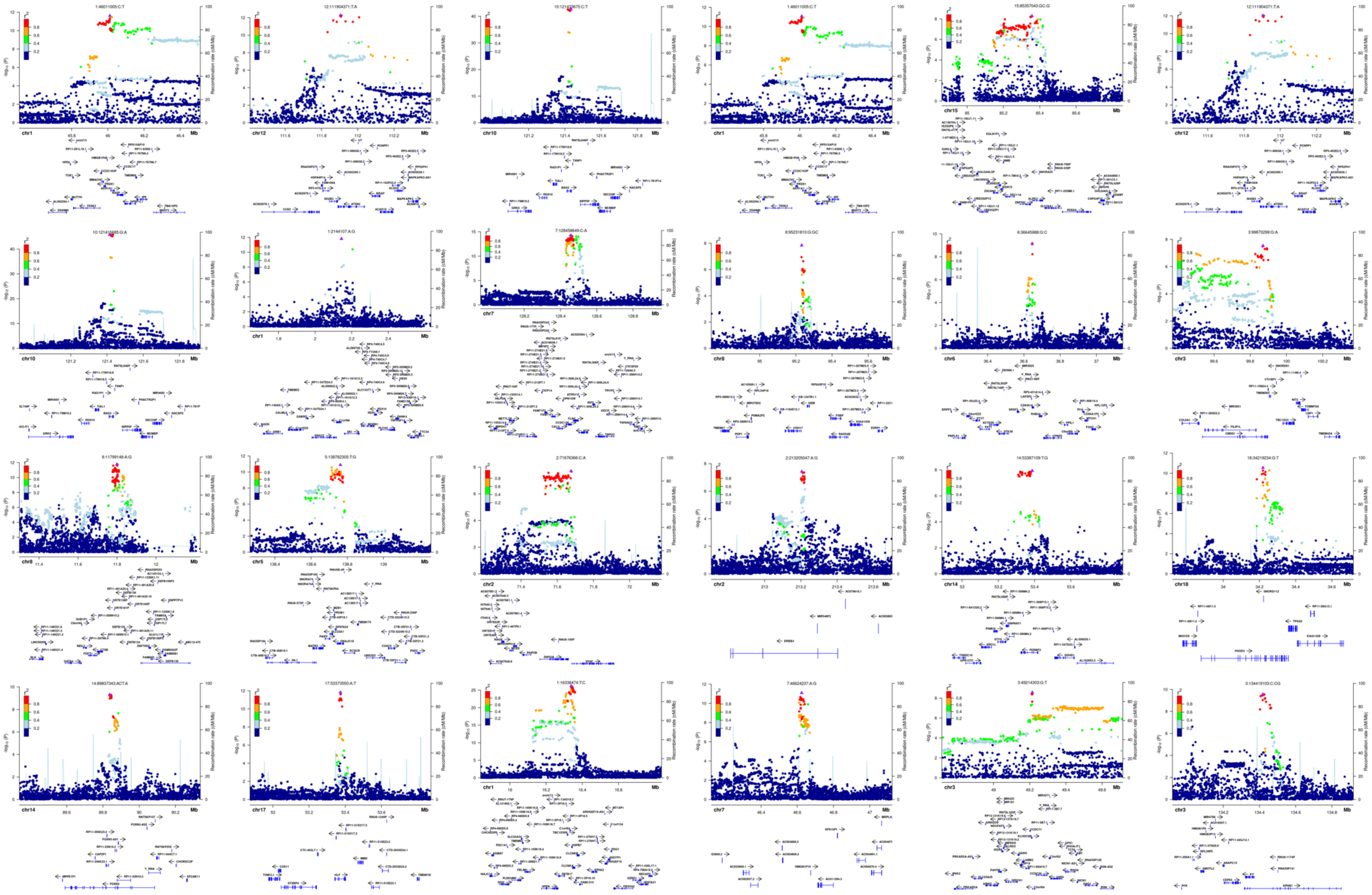

LVESV

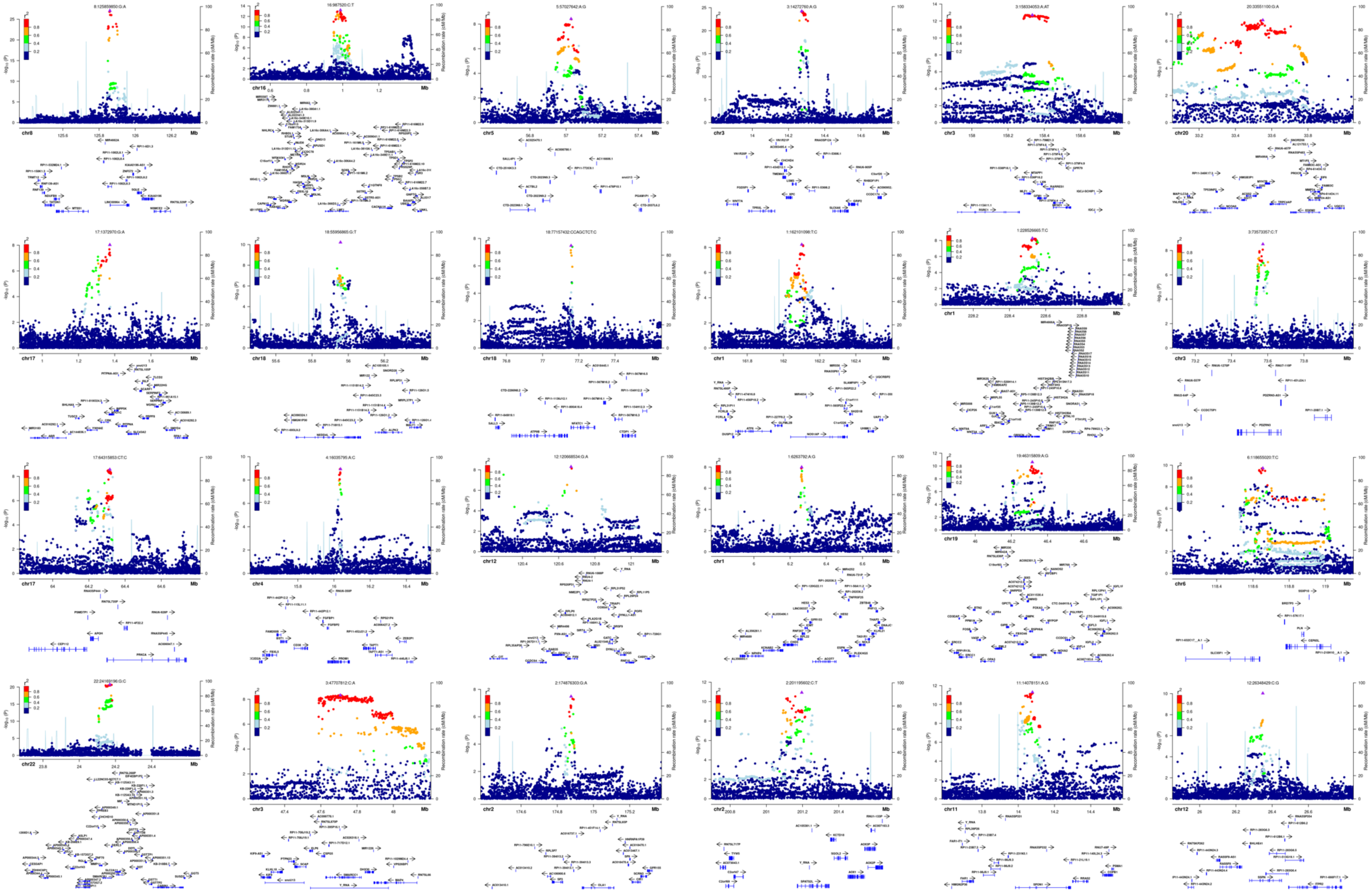

LVESV

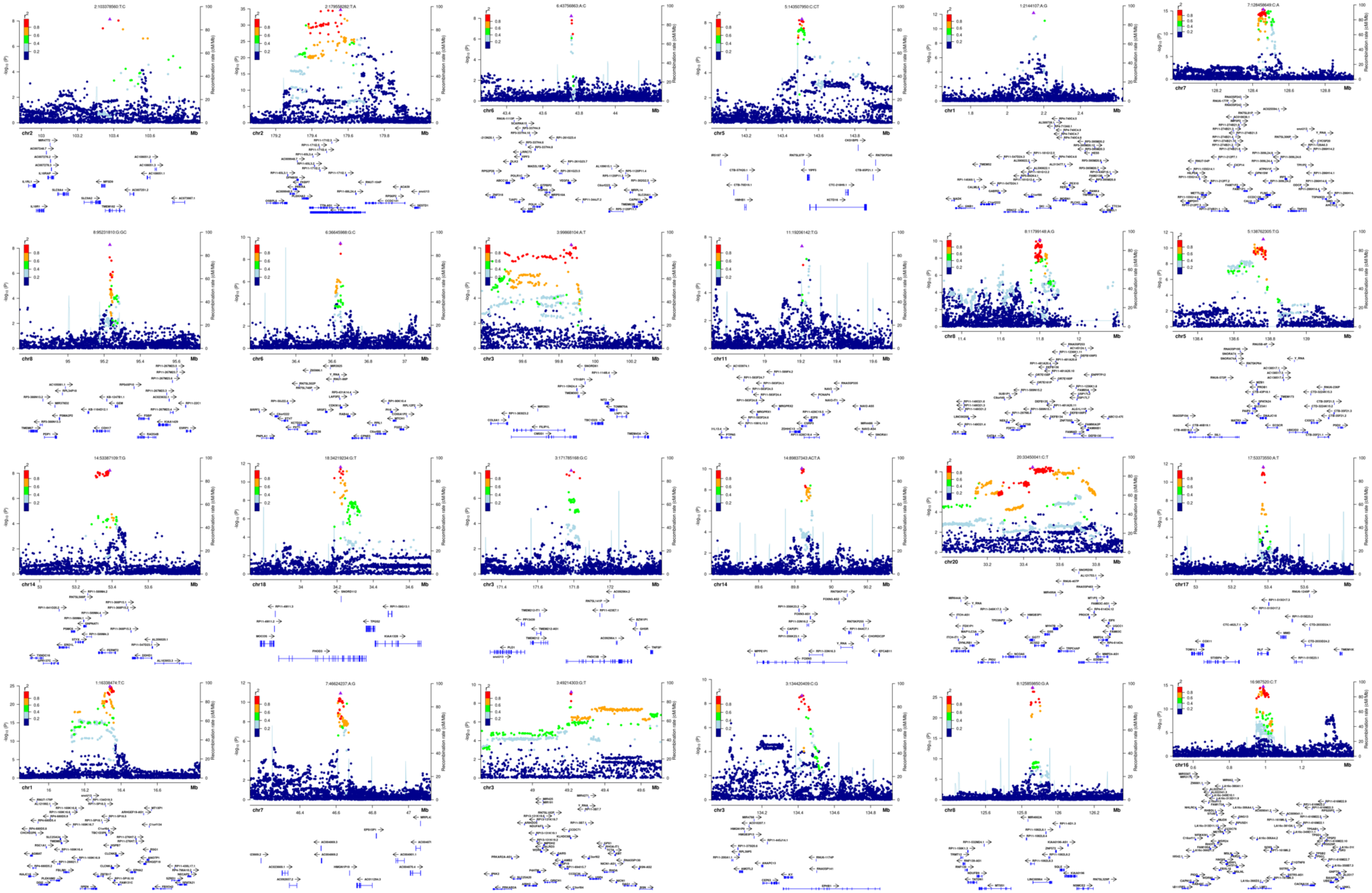

LVESV

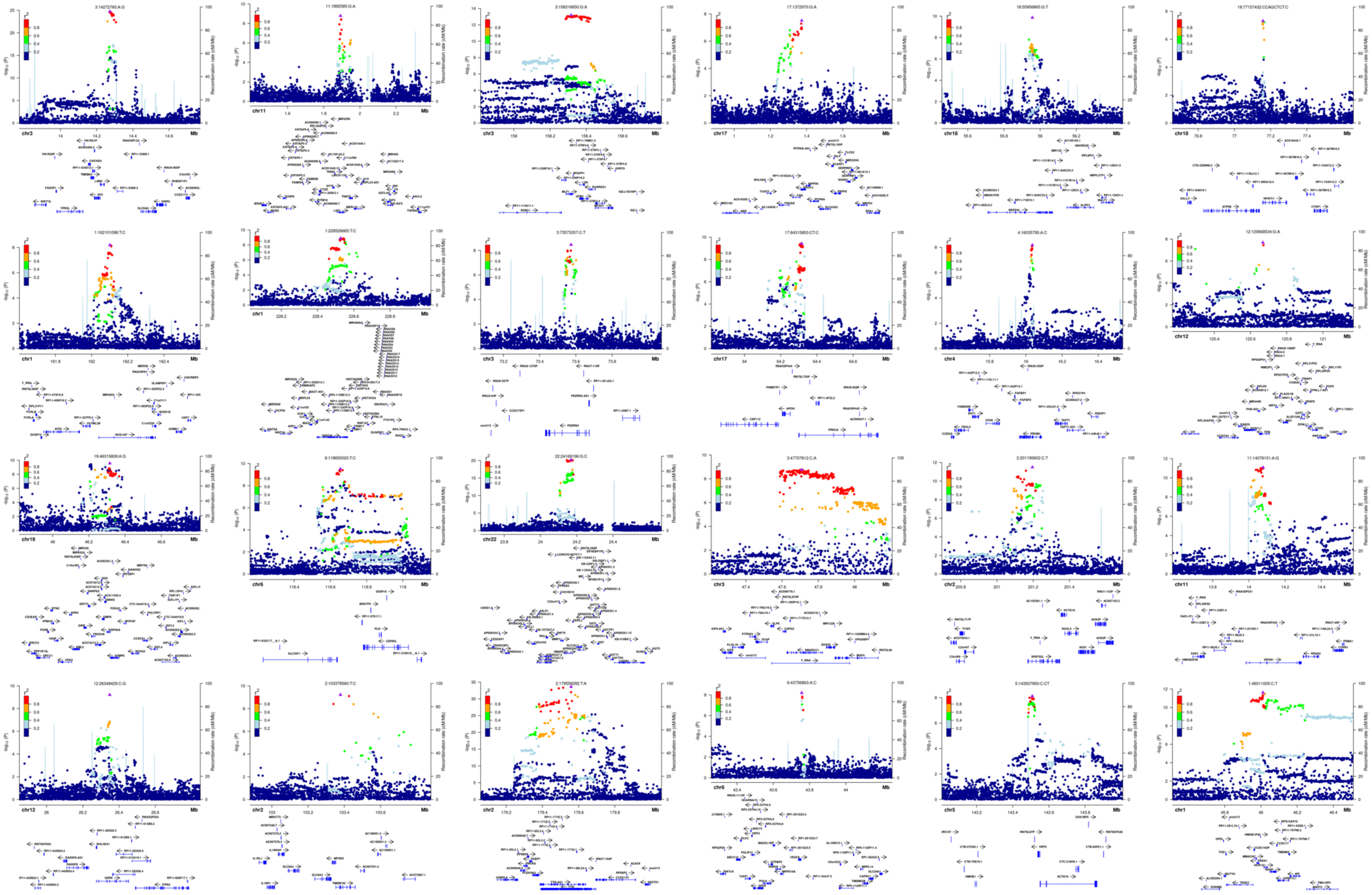

LVESV

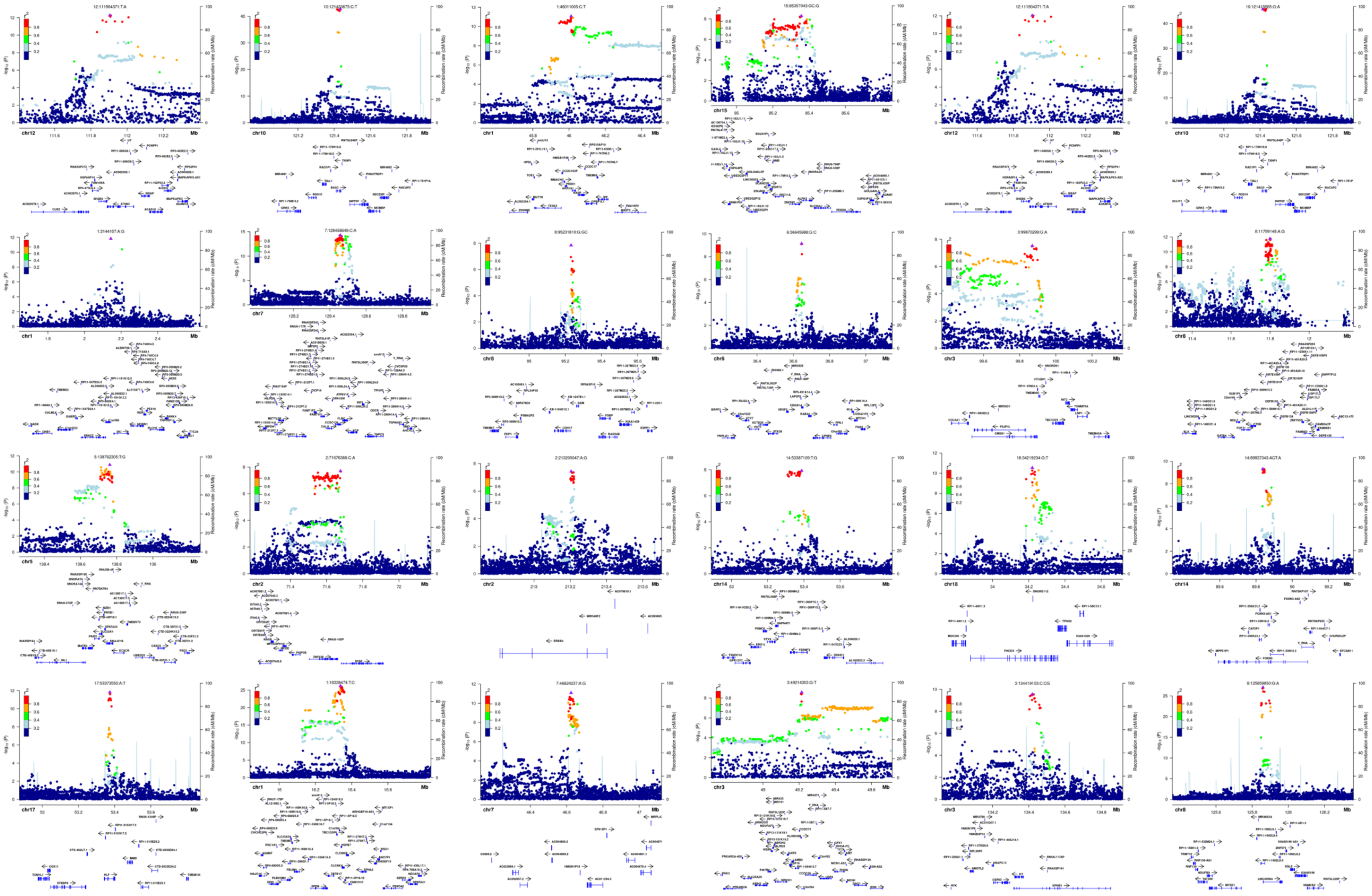

LVESV

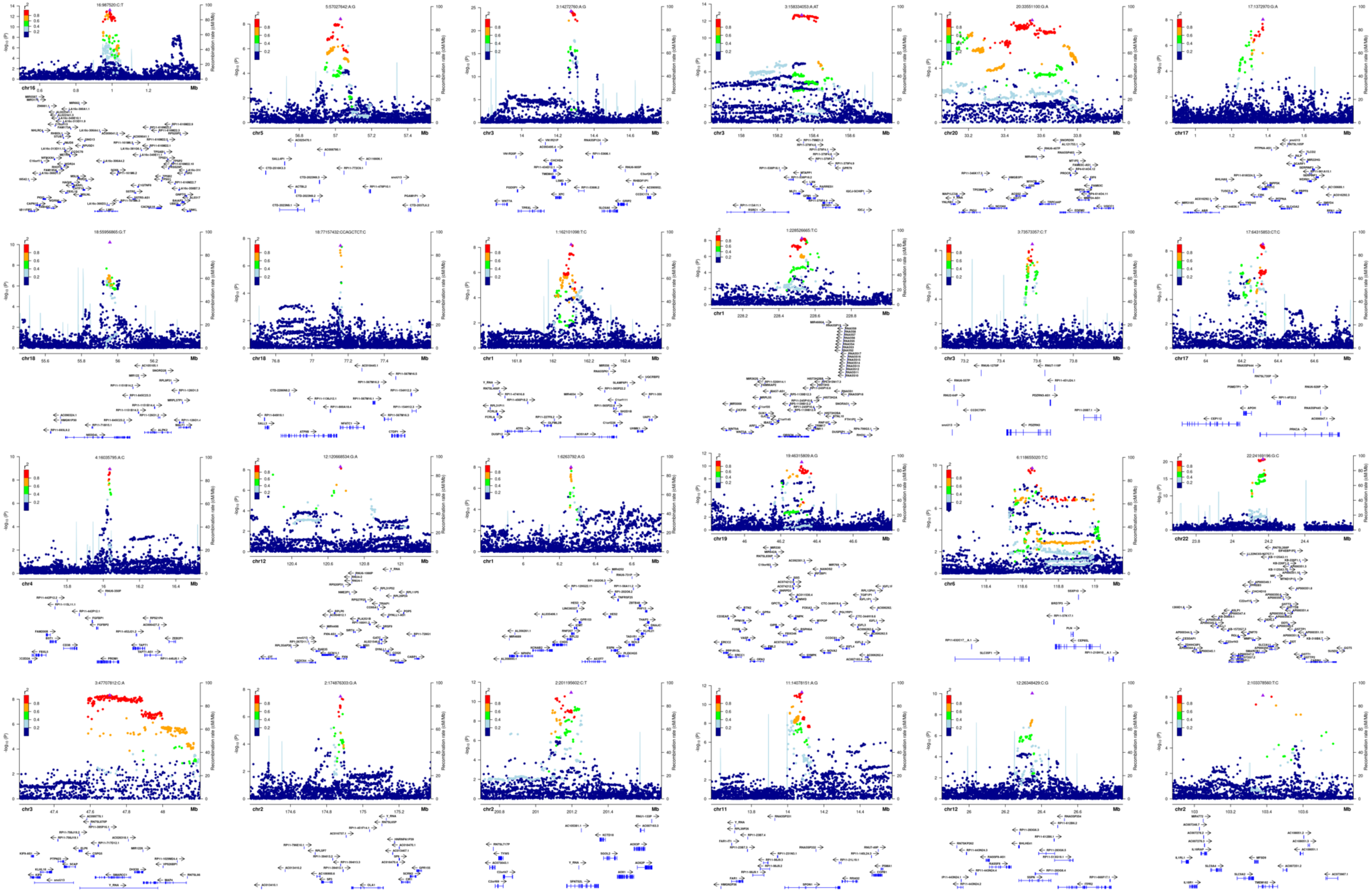
16:987520:C:T
5:57027642:A:G
3:14272760:A:G
3:158334053:A:AT
20:33551100:G:A
17:1372970:G:A
18:55956865:G:T
18:77157432:CCAGCTCT:C
1:162101098:T:C
1:228526665:T:C
3:73573357:C:T
17:64315853:CT:C
4:16035795:A:C
12:120668534:G:A
1:6263792:A:G
19:46315809:A:G
6:118655020:T:C
22:24169196:G:C
3:47707812:C:A
2:174876303:G:A
2:201195602:C:T
11:14078151:A:G
12:26349429:C:G
2:103378560:T:C

LVESV

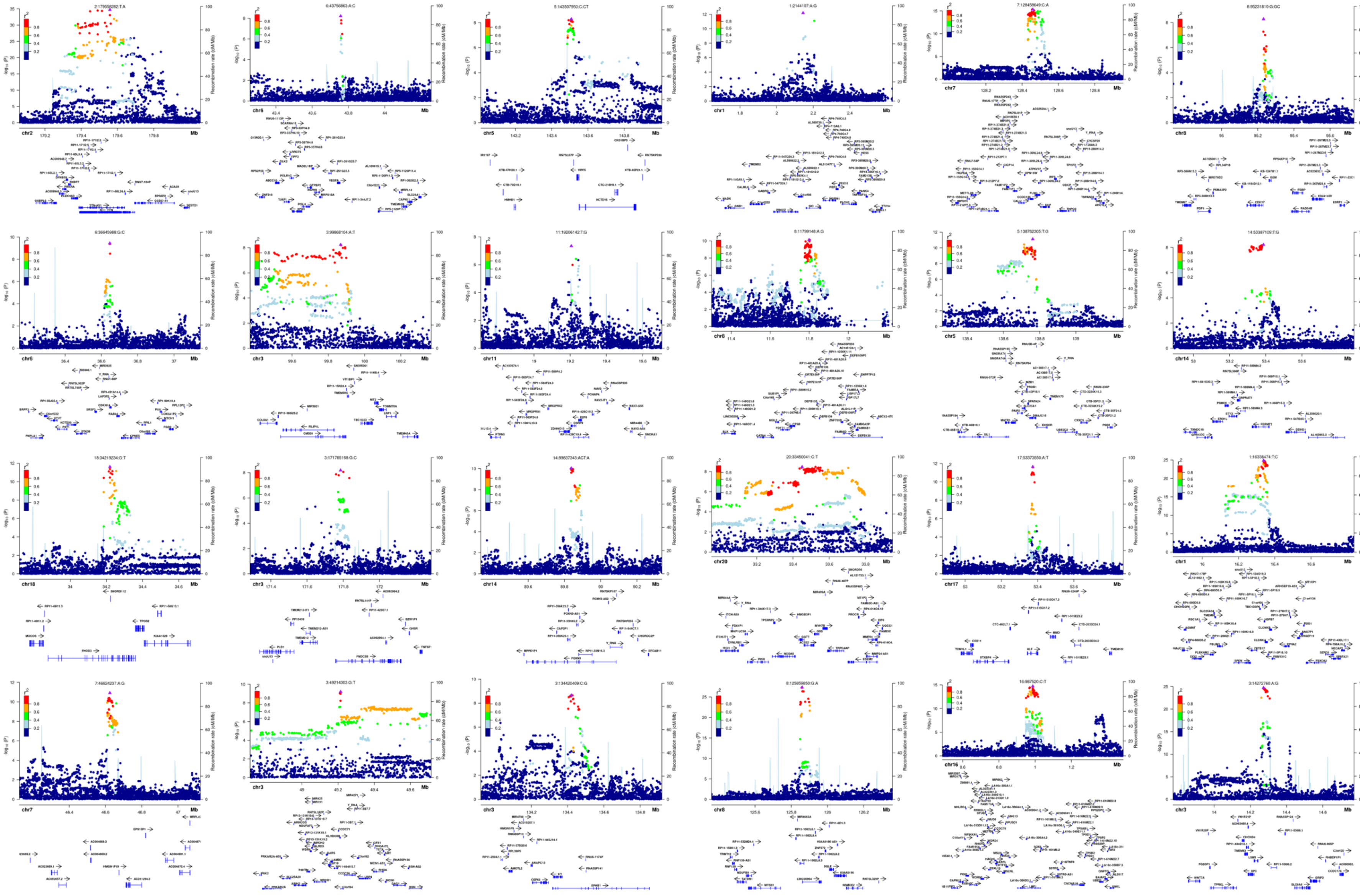

LVESV

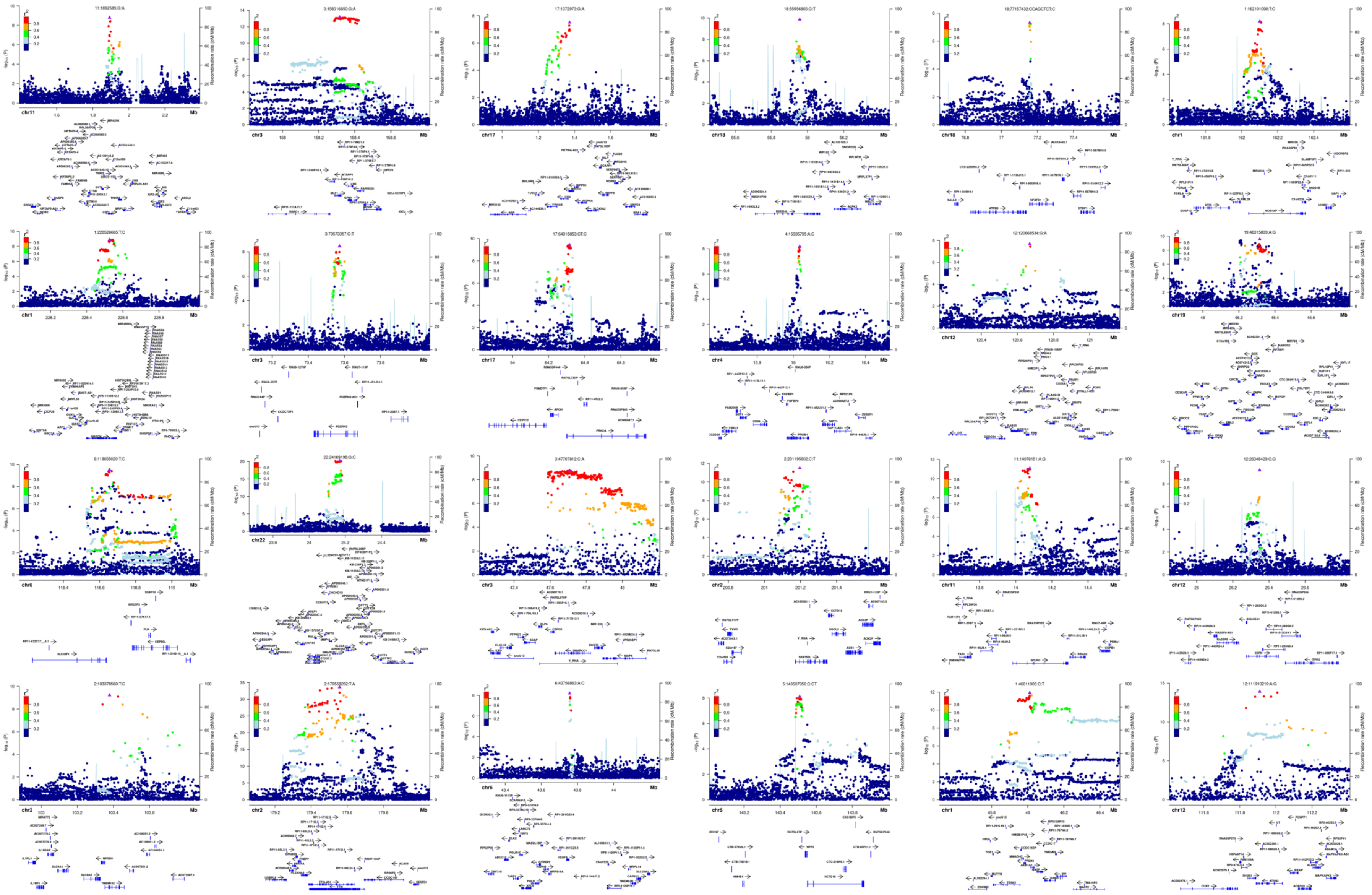

LVESV

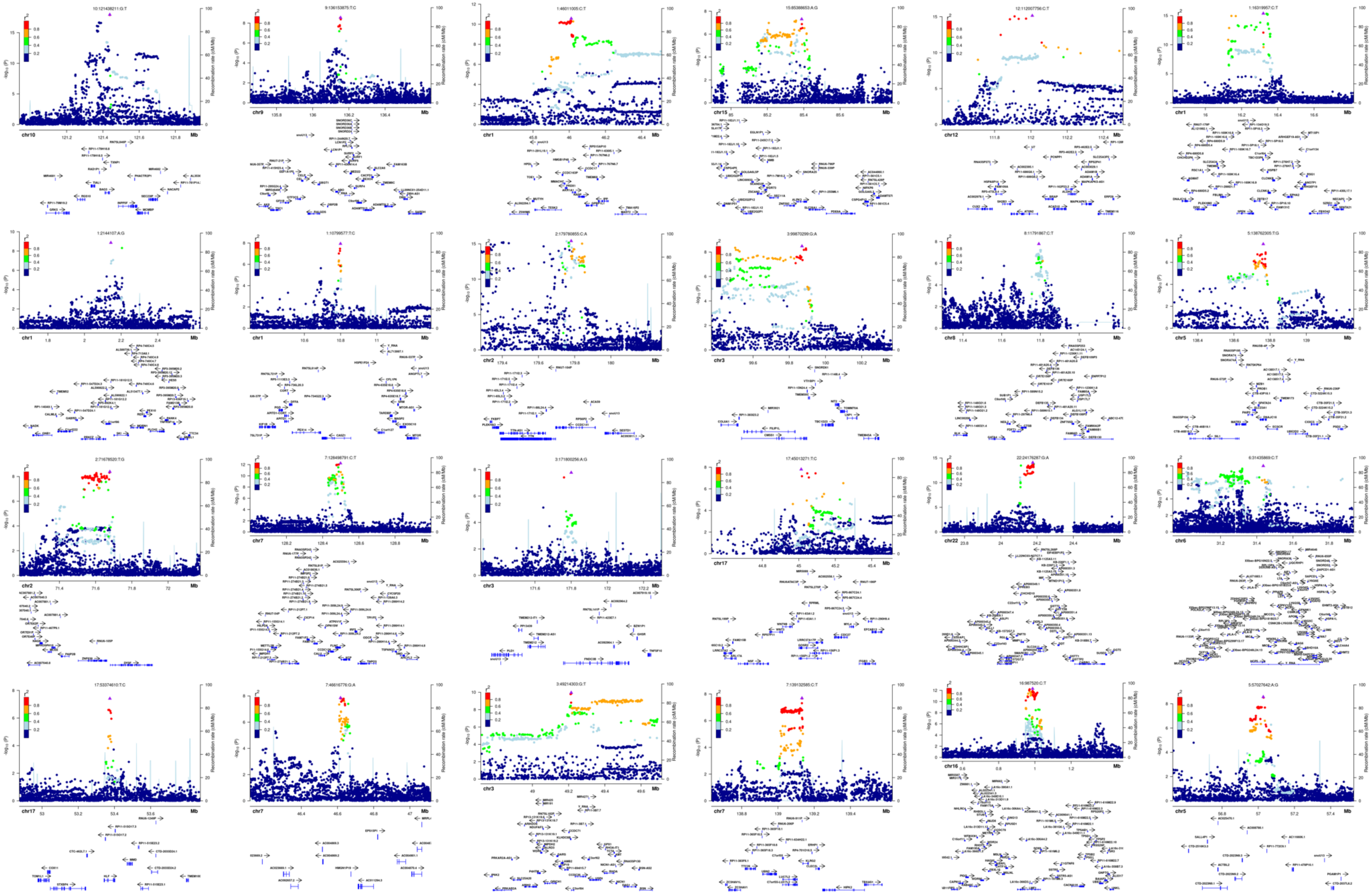
10:121436211:G:T
9:136153875:T:C
1:46011005:C:T
15:85388653:A:G
12:112007756:C:T
1:16319957:C:T
1:2144107:A:G
1:10799577:T:C
2:179780855:C:A
3:99870299:G:A
8:11791867:C:T
5:138762305:T:G
2:71678520:T:G
7:128498791:C:T
3:171800256:A:G
17:45013271:T:C
22:24176287:G:A
6:31435869:C:T
17:53374610:T:C
7:46616776:G:A
3:49214303:G:T
7:139132585:C:T
16:987520:C:T
5:57027642:A:G

LVESV

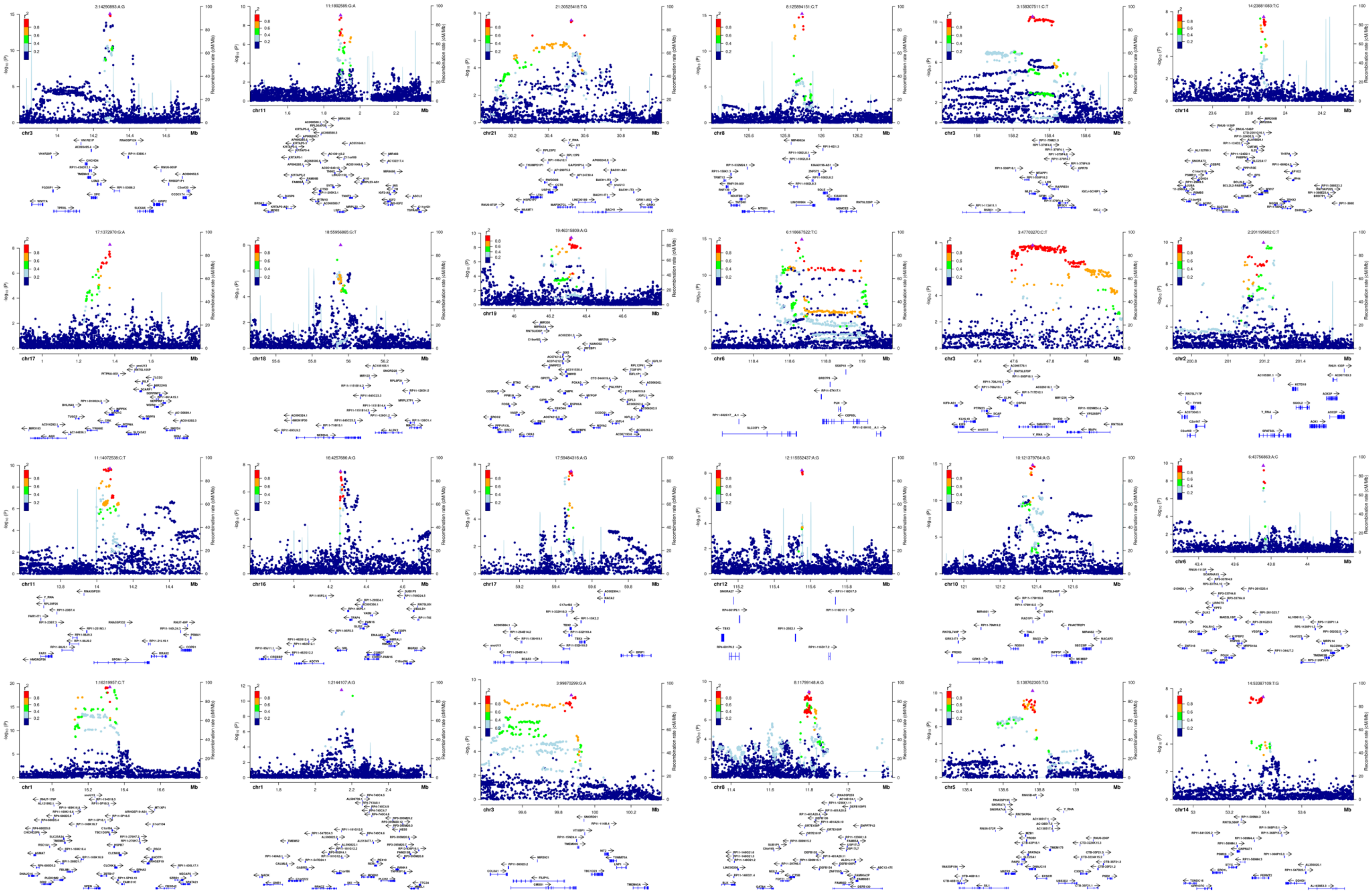

LVESV

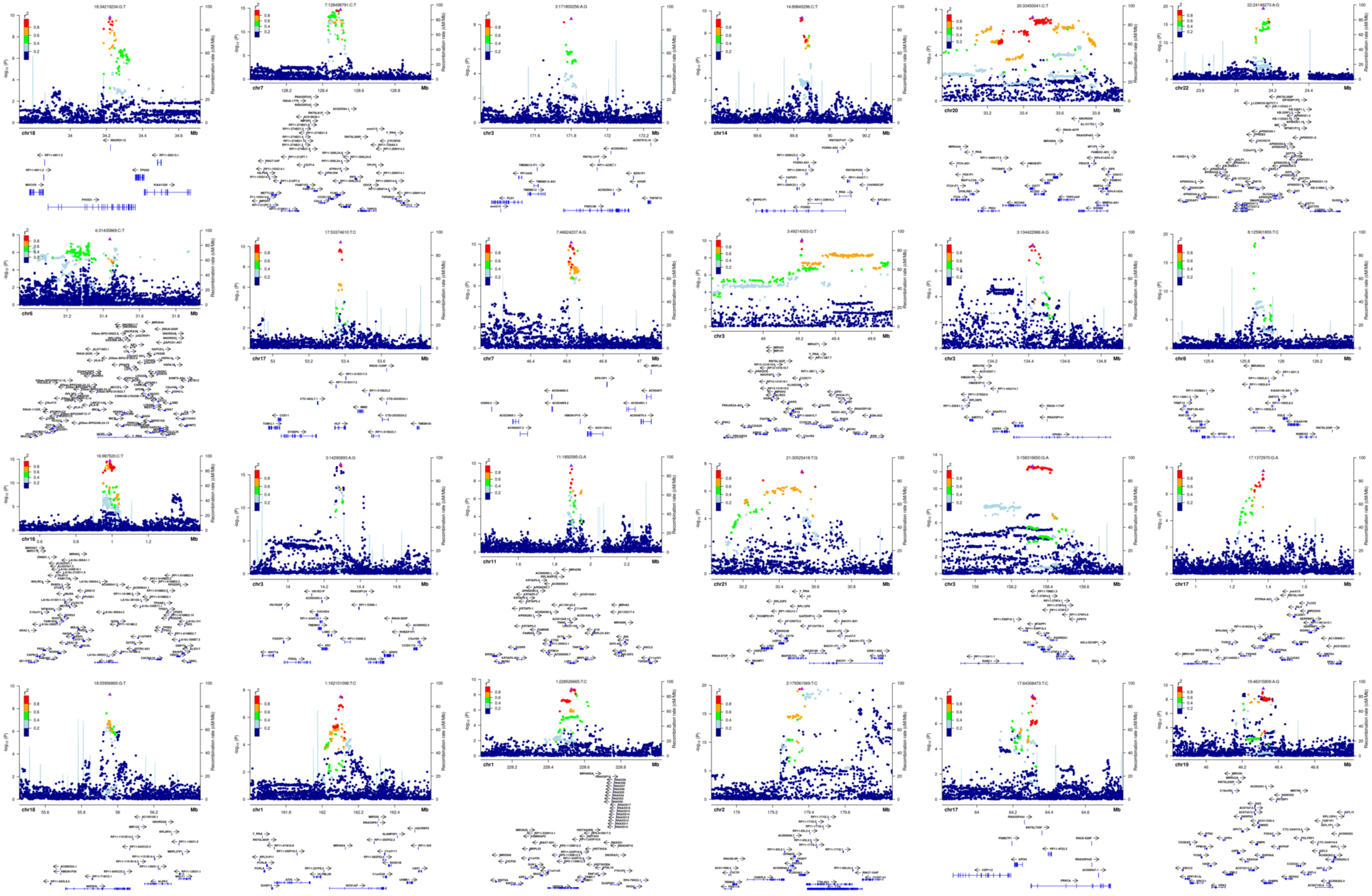

LVESV

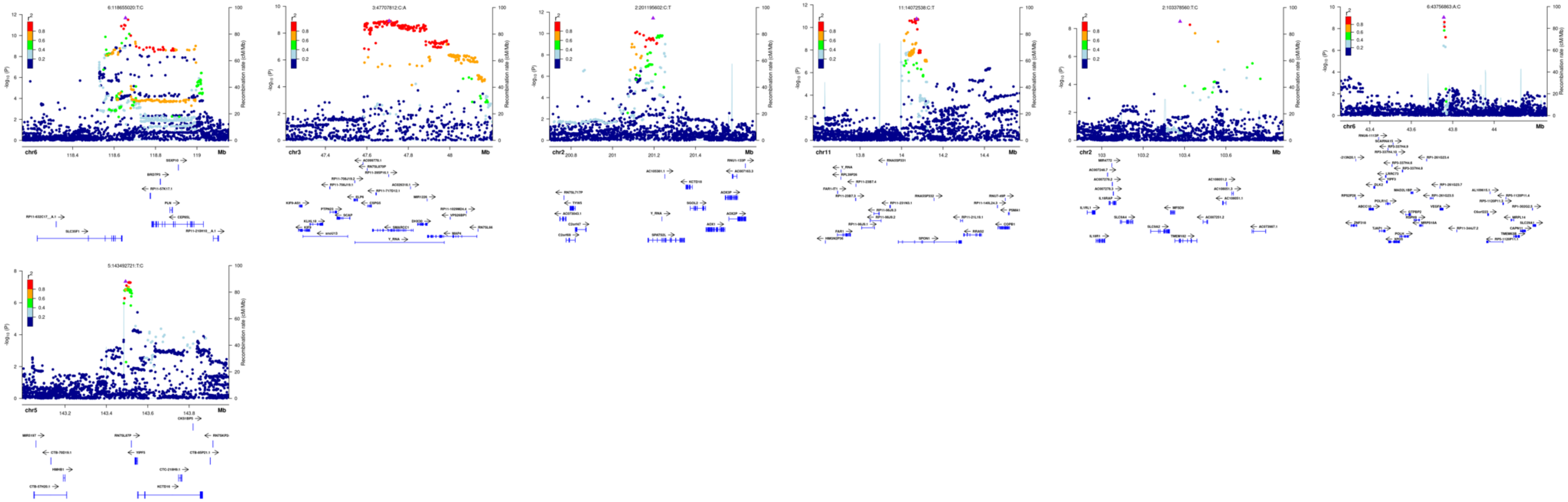
6:118655020:T:C
3:47707812:C:A
2:201195602:C:T
11:14072538:C:T
2:103378560:T:C
6:43756863:A:C
5:143492721:T:C

LVESV_BSA

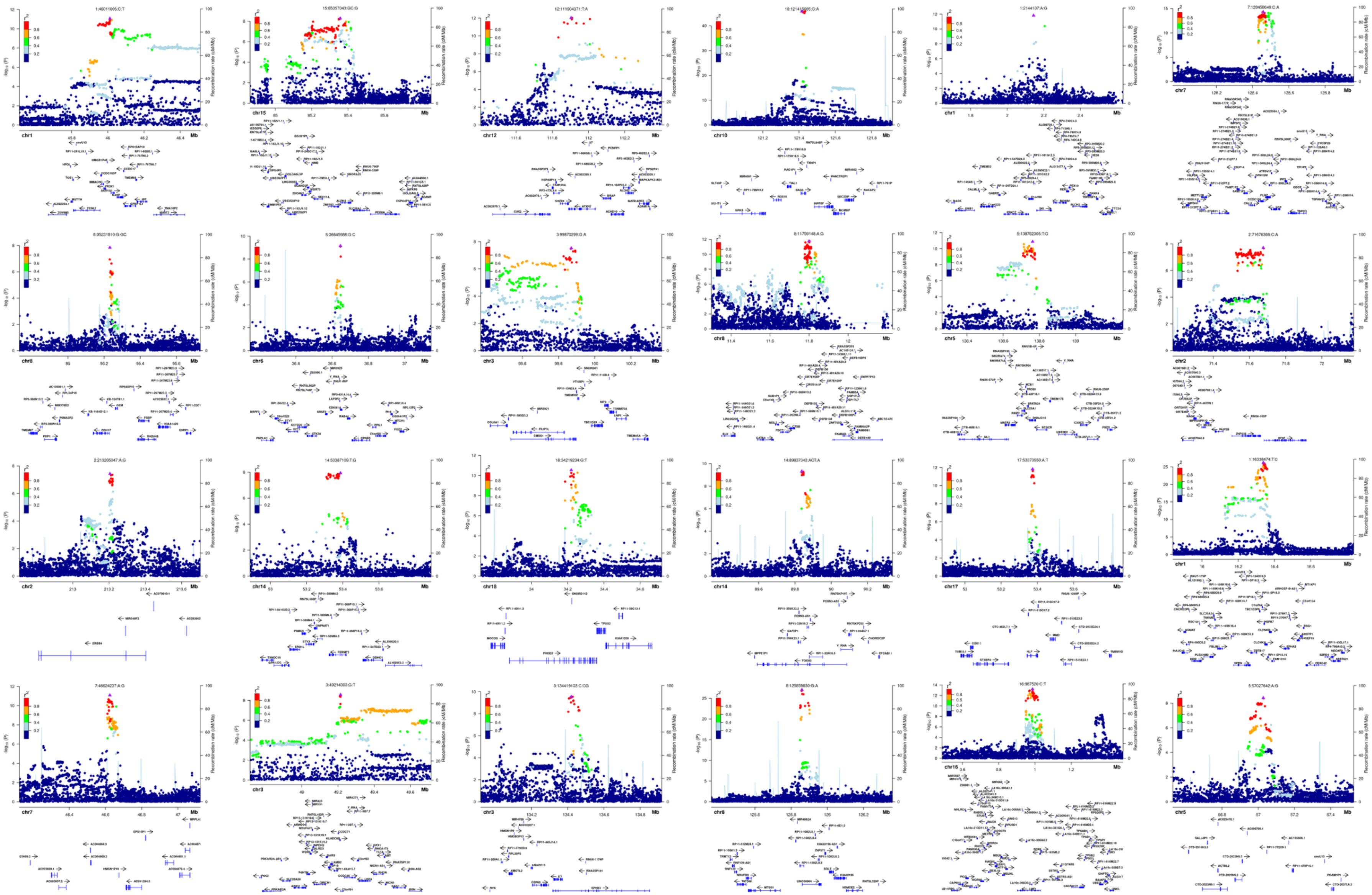

LVESV_BSA

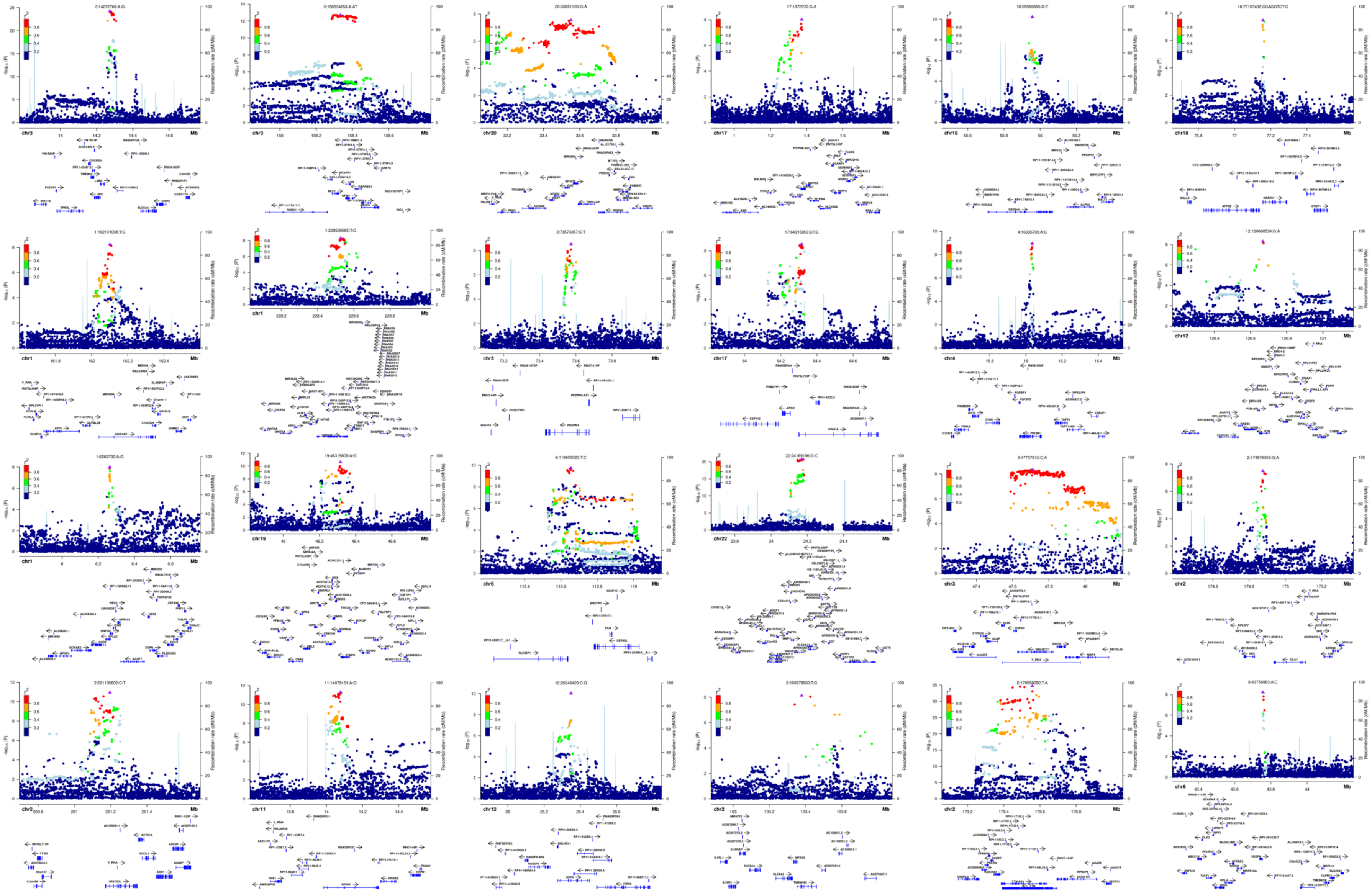

LVESV_BSA

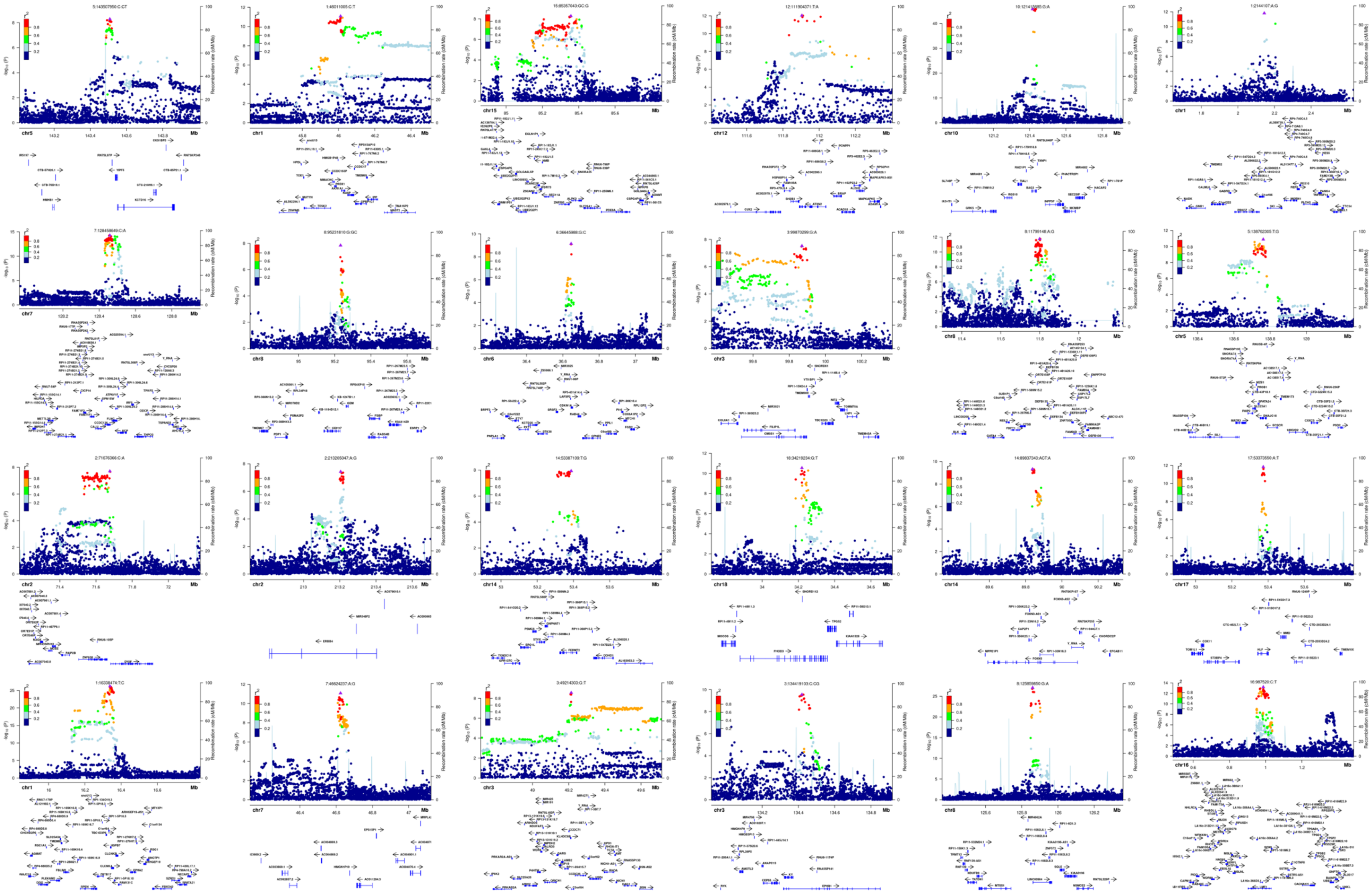

5:143507950:C:CT
1:46011005:C:T
15:85357043:GC:G
12:111904371:T:A
10:121415685:G:A
1:2144107:A:G
7:128458649:C:A
8:95231810:G:GC
6:36645988:G:C
3:99870299:G:A
8:11799148:A:G
5:138762305:T:G
2:71676366:C:A
2:213205047:A:G
14:53387109:T:G
18:34219234:G:T
14:89837343:ACT:A
17:53373550:A:T
1:16338474:T:C
7:46624237:A:G
3:49214303:G:T
3:134419103:C:CG
8:125859850:G:A
16:987520:C:T

LVESV_BSA

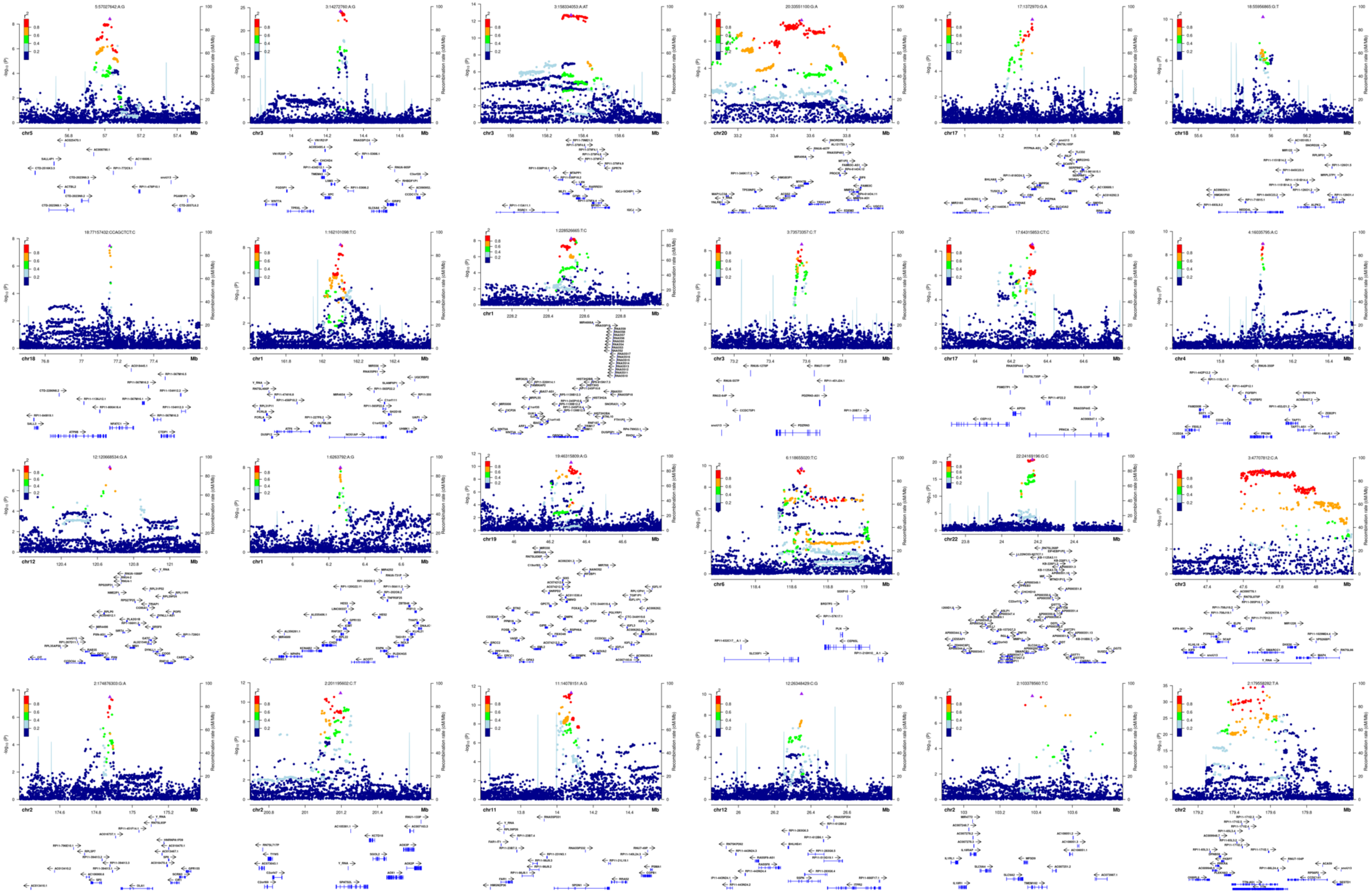

LVESV_BSA

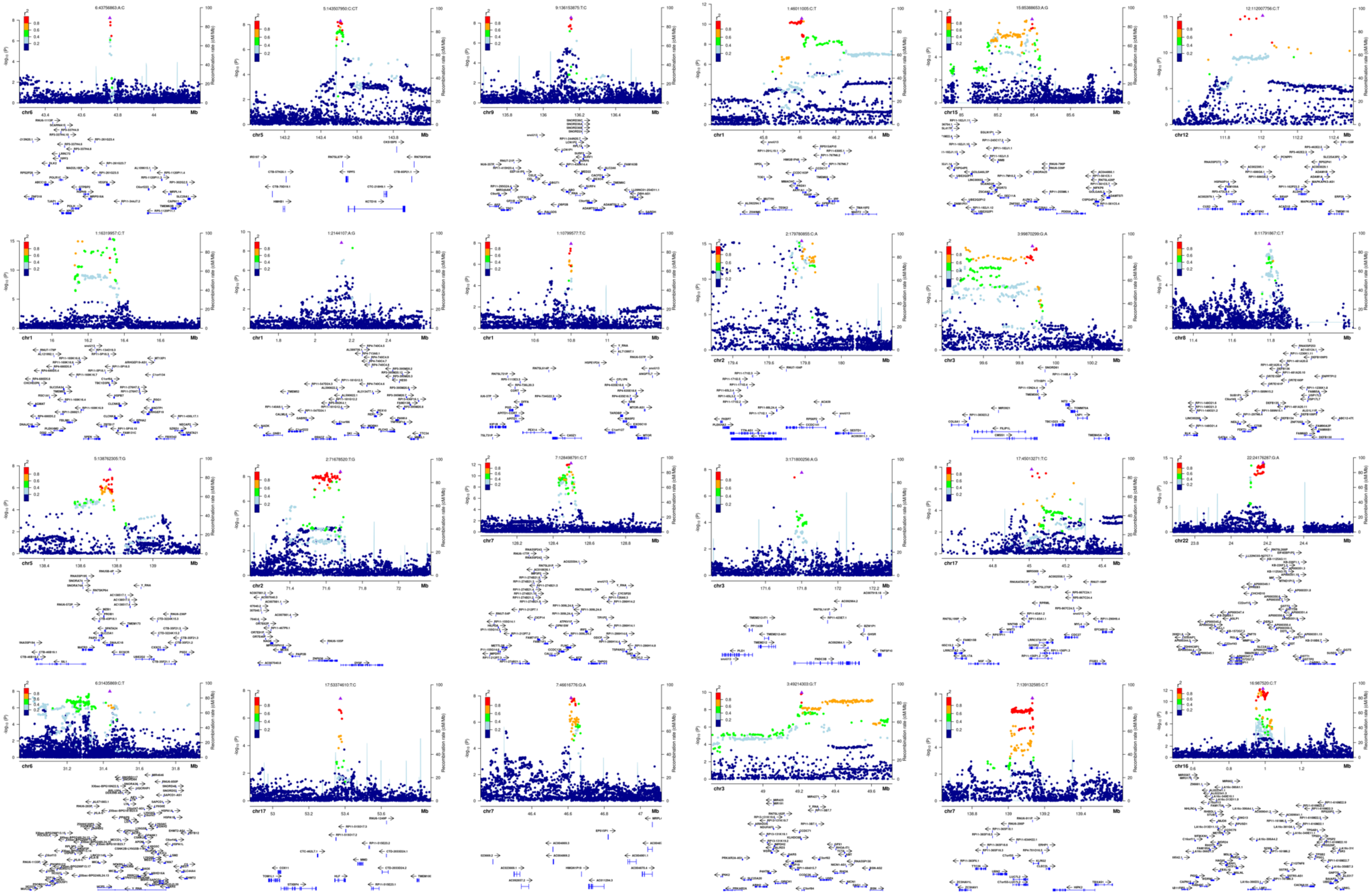

LVESV_BSA

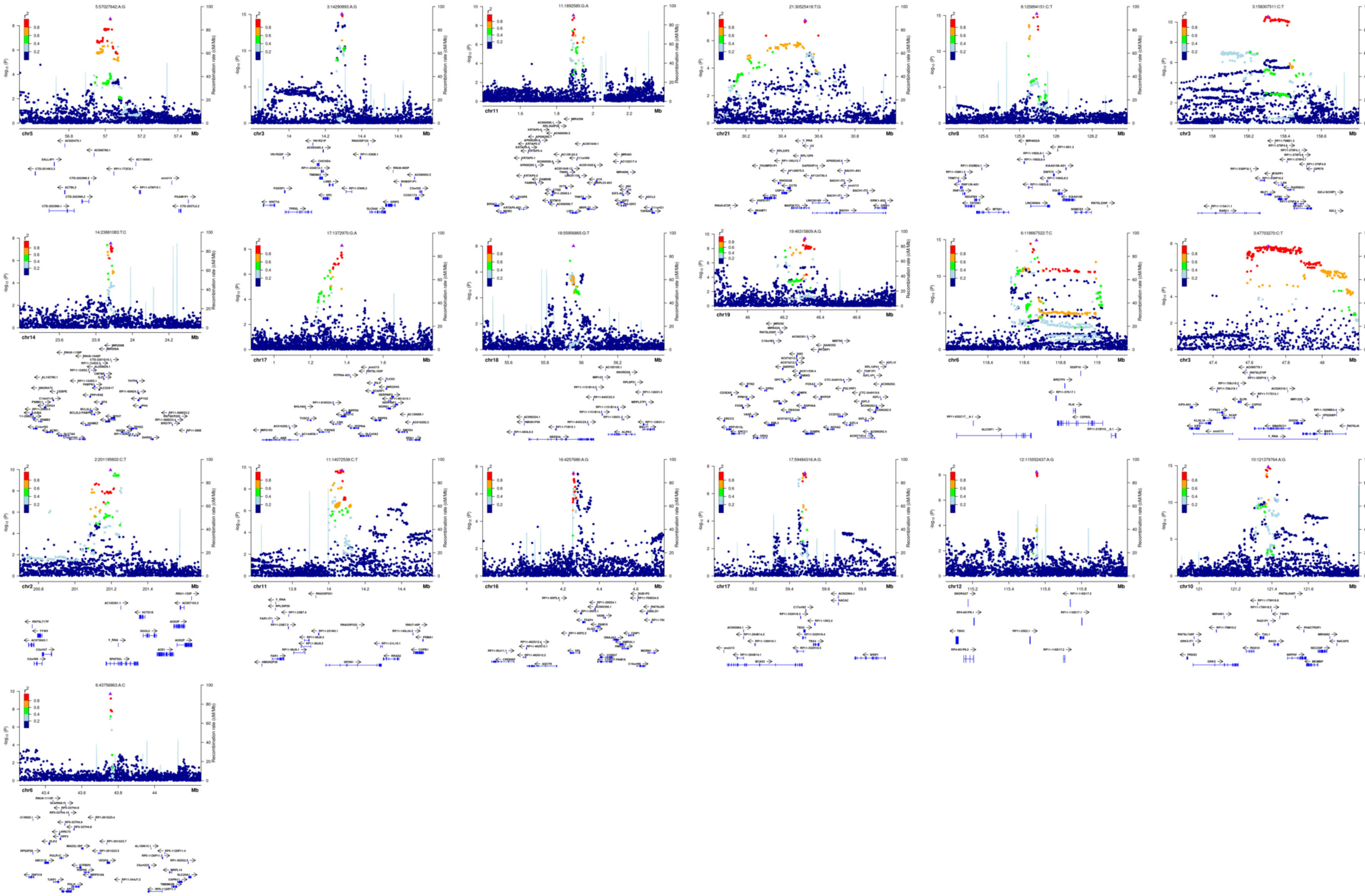

LVGFI

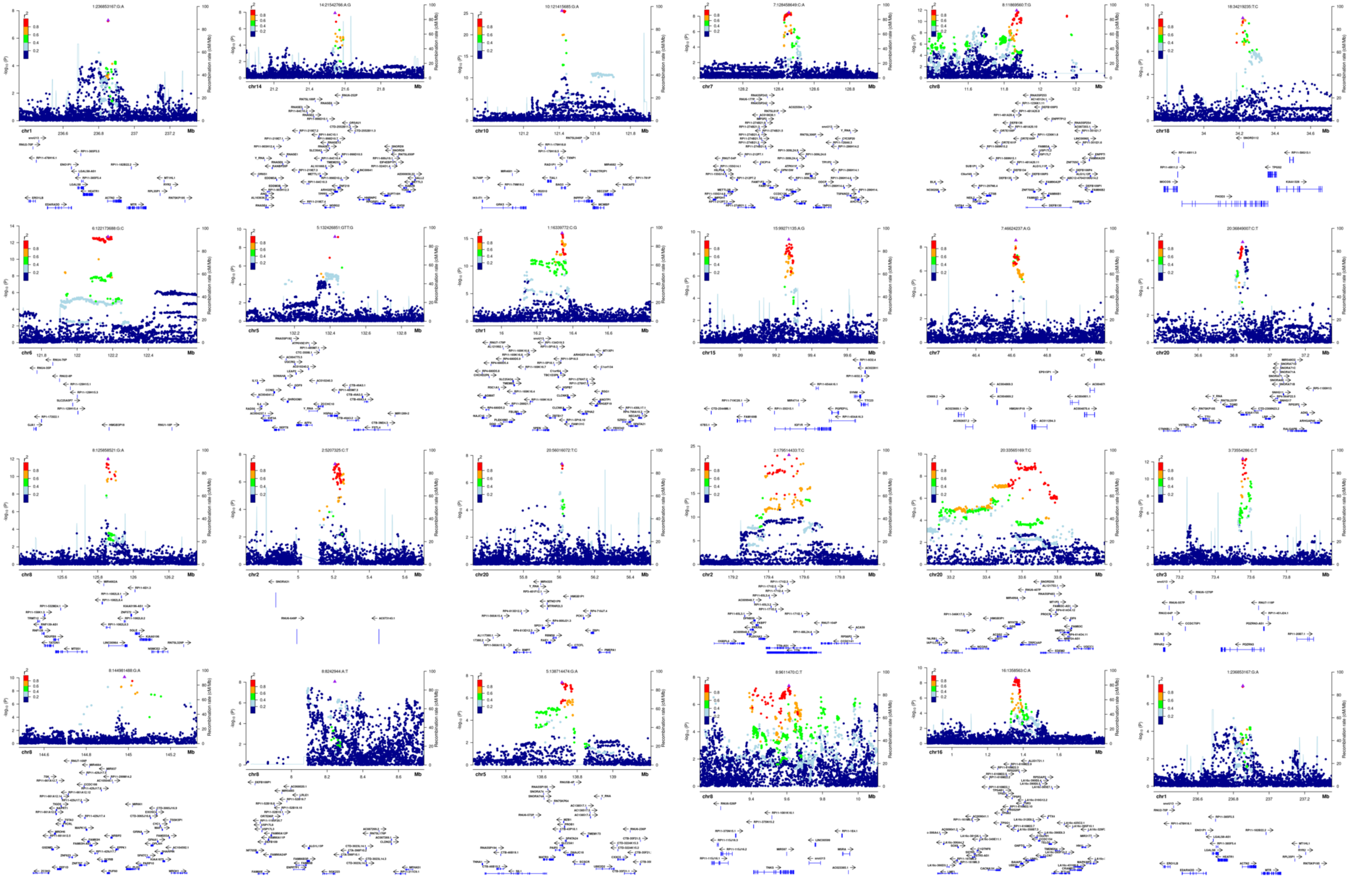

LVGFI

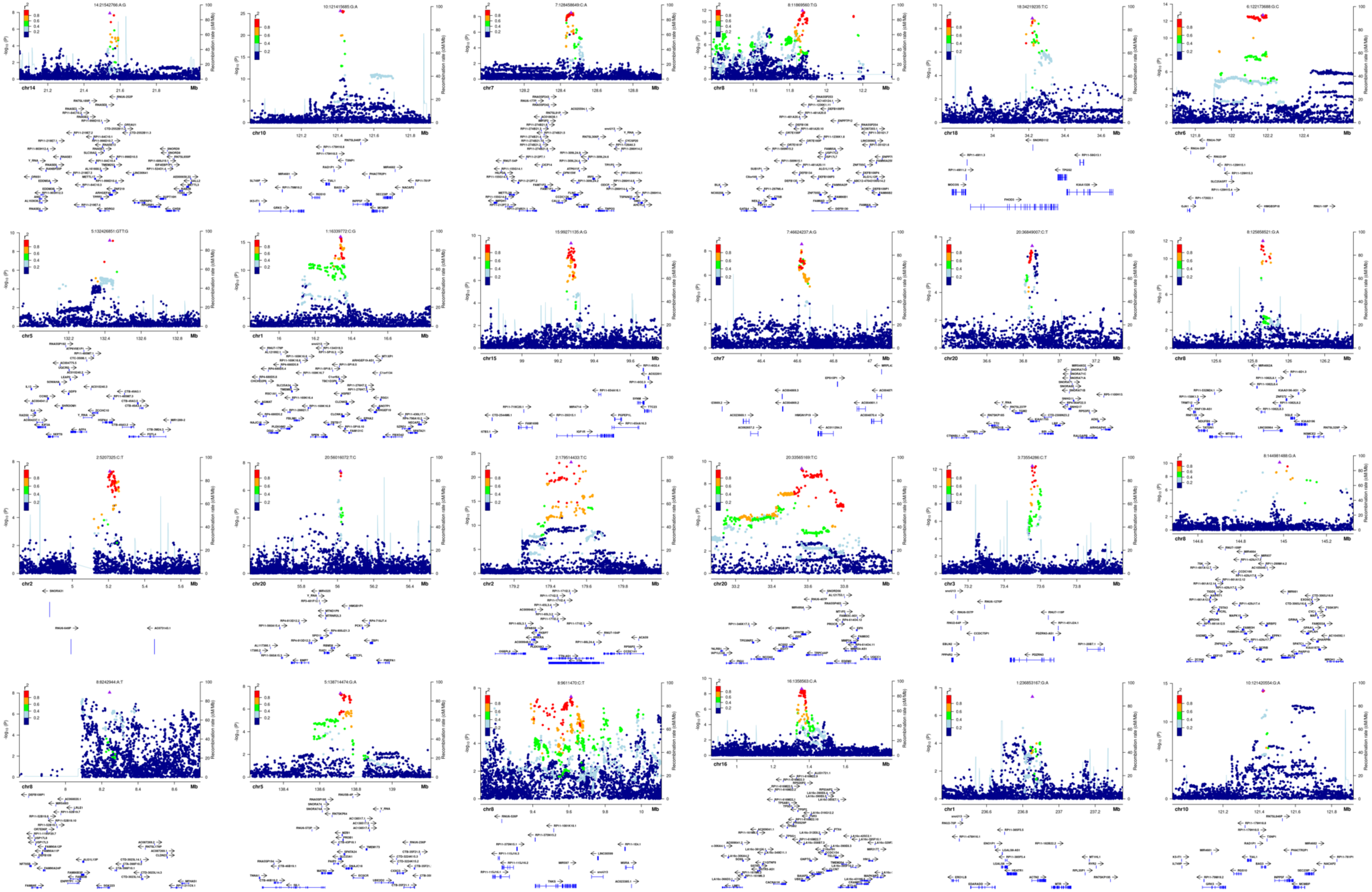
14:21542766:A:G
10:121415685:G:A
7:128458649:C:A
8:11869560:T:G
18:34219235:T:C
6:122173688:G:C
5:132426851:GTT:G
1:16339772:C:G
15:99271135:A:G
7:46624237:A:G
20:36849007:C:T
8:125858521:G:A
2:5207325:C:T
20:56016072:T:C
2:179514433:T:C
20:33565169:T:C
3:73554286:C:T
8:144981498:G:A
8:8242944:A:T
5:138714474:G:A
8:9611470:C:T
16:1358563:C:A
1:236853167:G:A
10:121420554:G:A

LVGFI

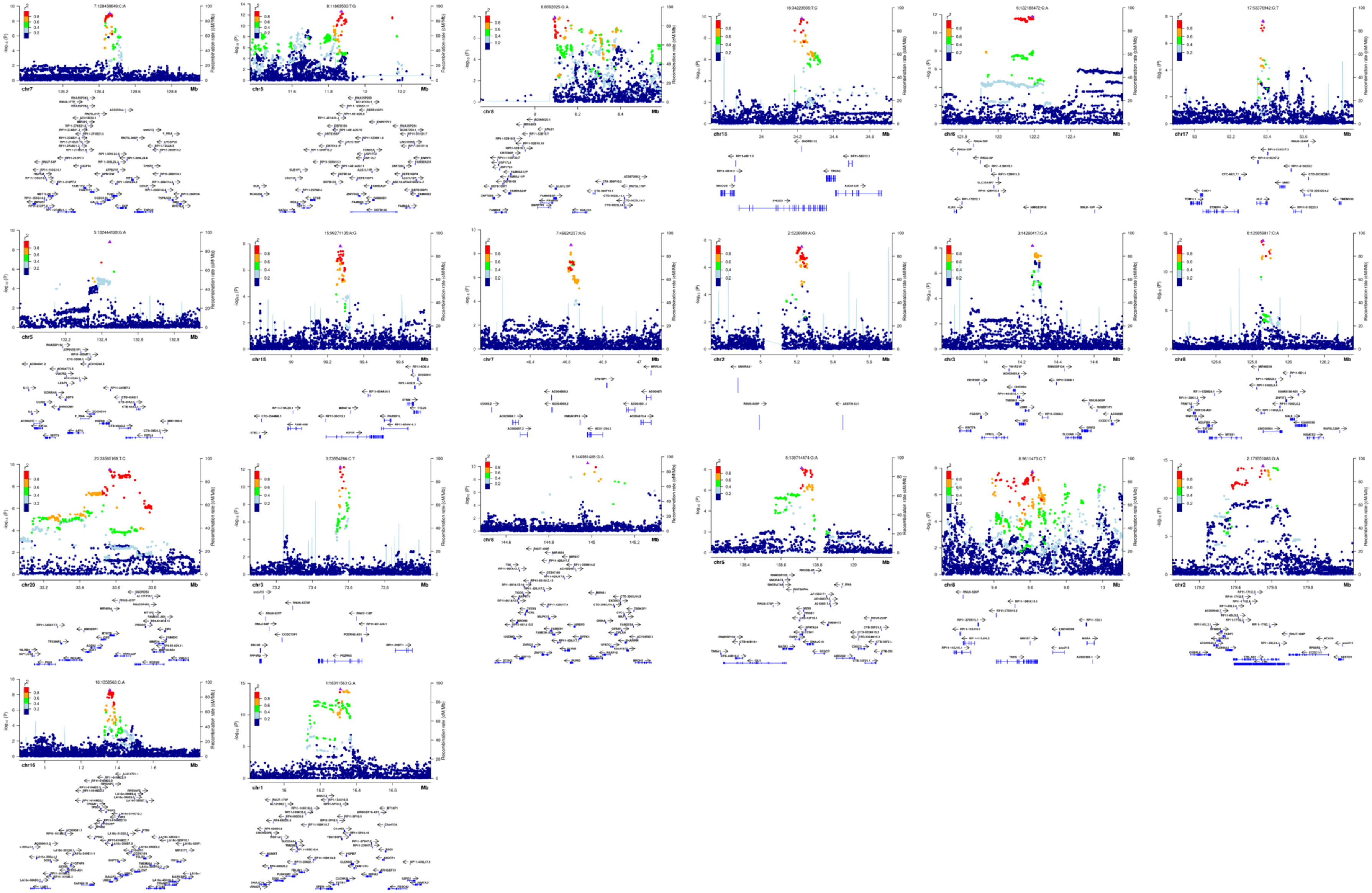
7:128458649:C:A
8:11869560:T:G
8:8092025:G:A
18:34223566:T:C
6:122188472:C:A
17:53376942:C:T
5:132444128:G:A
15:99271135:A:G
7:46624237:A:G
2:5226989:A:G
3:14260417:G:A
8:125859817:C:A
20:33565169:T:C
3:73554286:C:T
8:144981488:G:A
5:138714474:G:A
8:9611470:C:T
2:179551083:G:A
16:1358563:C:A
1:16311563:G:A

LVM

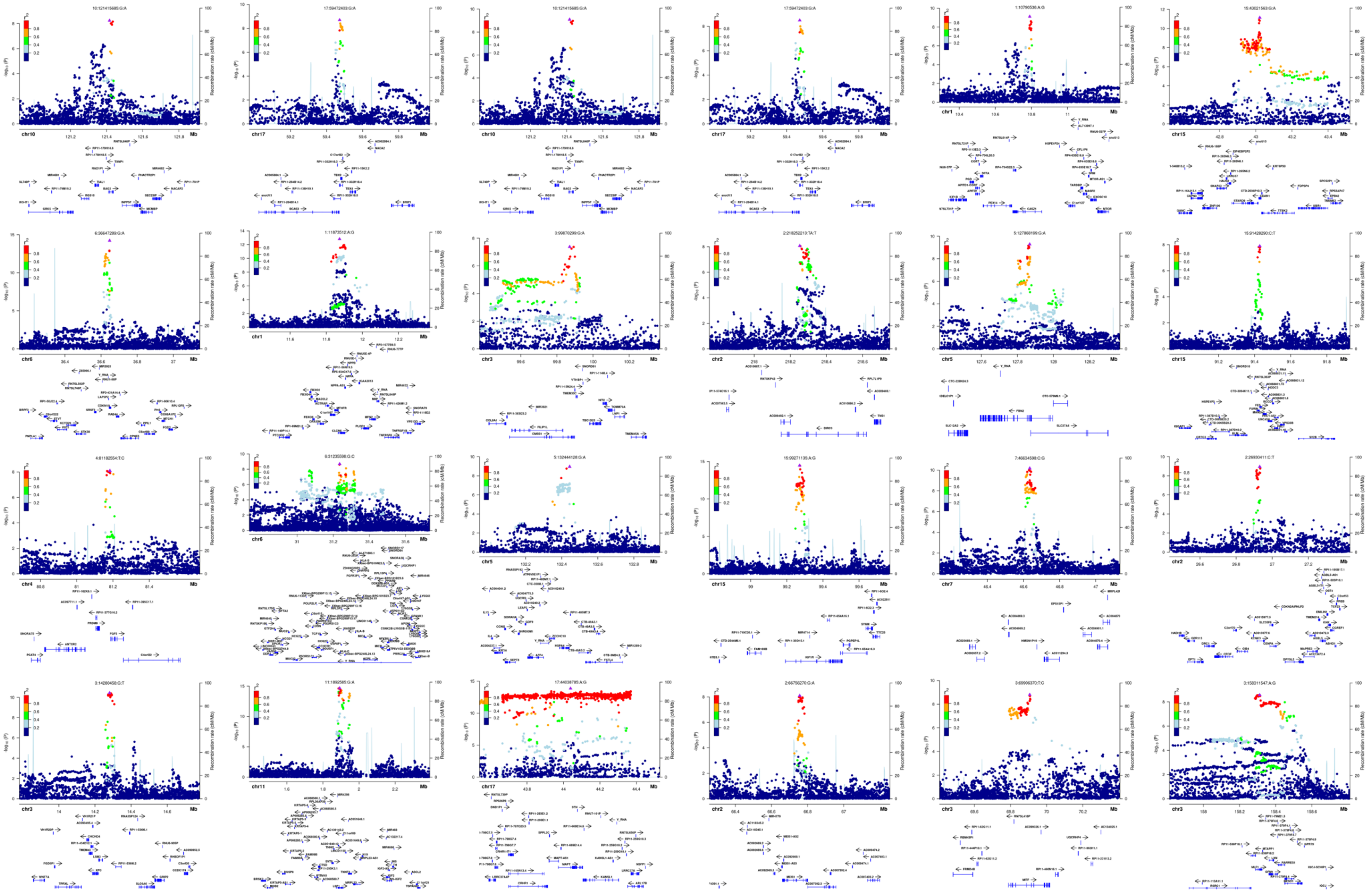

LVM

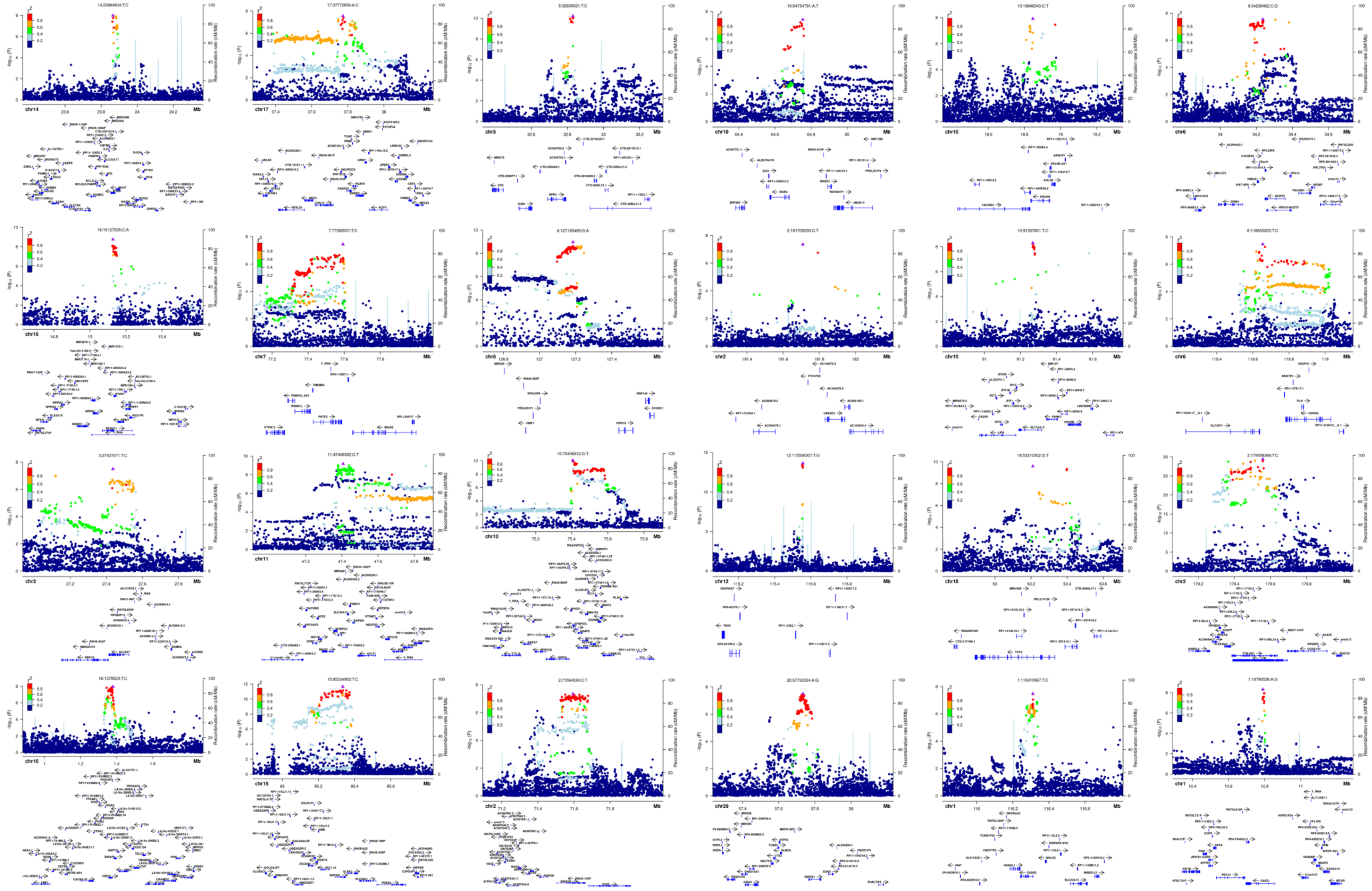

LVM

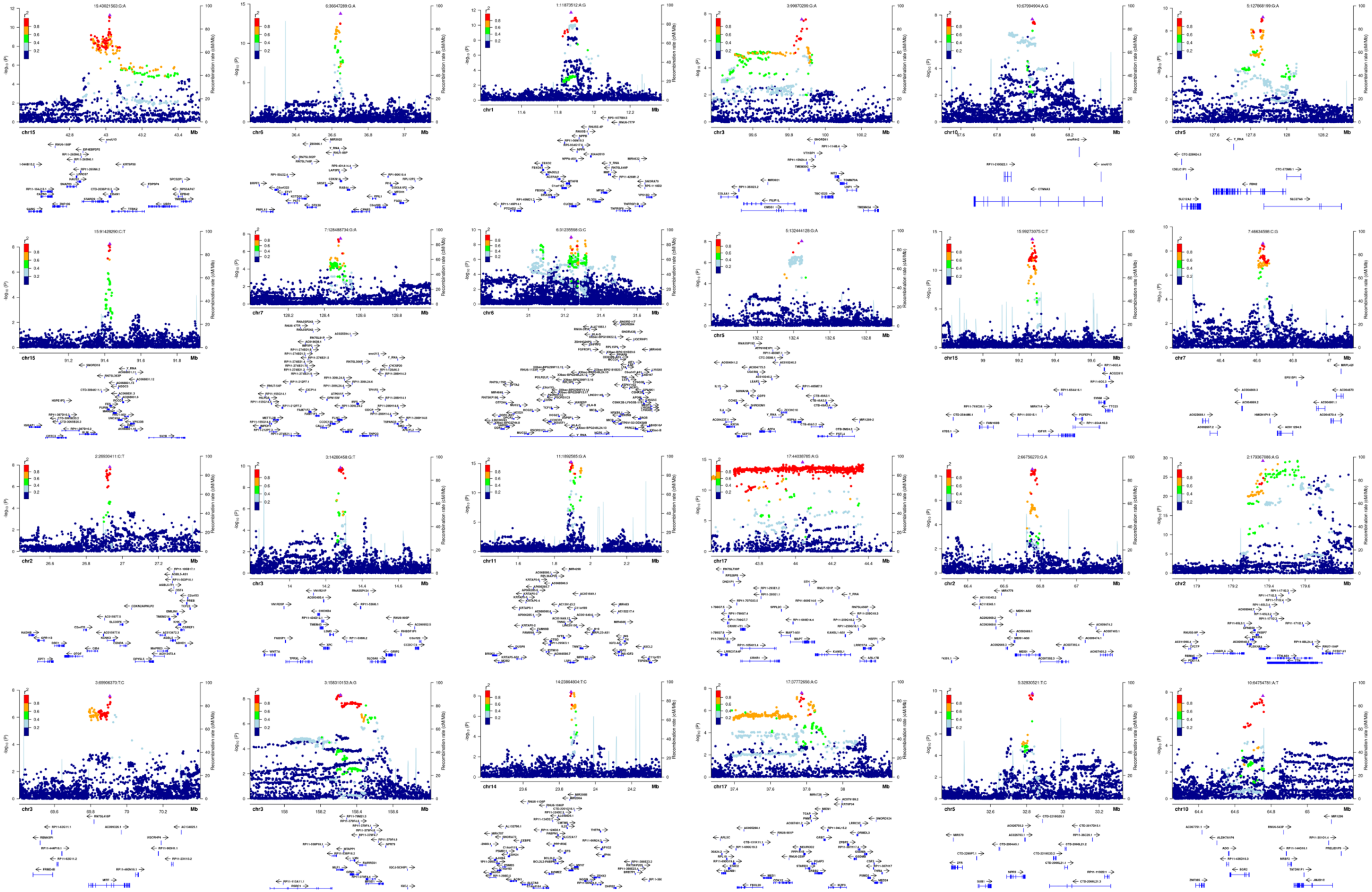

LVM

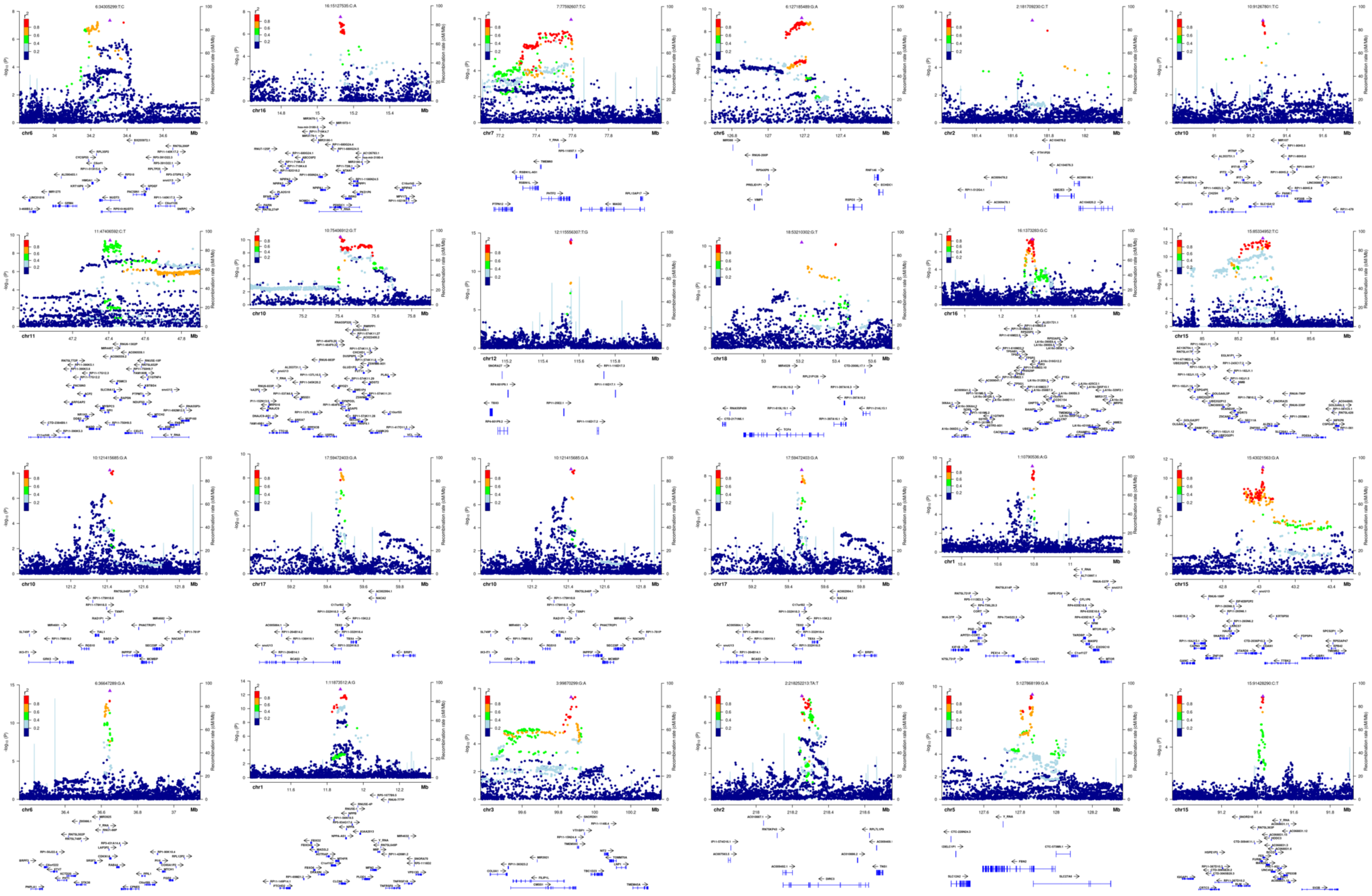

LVM

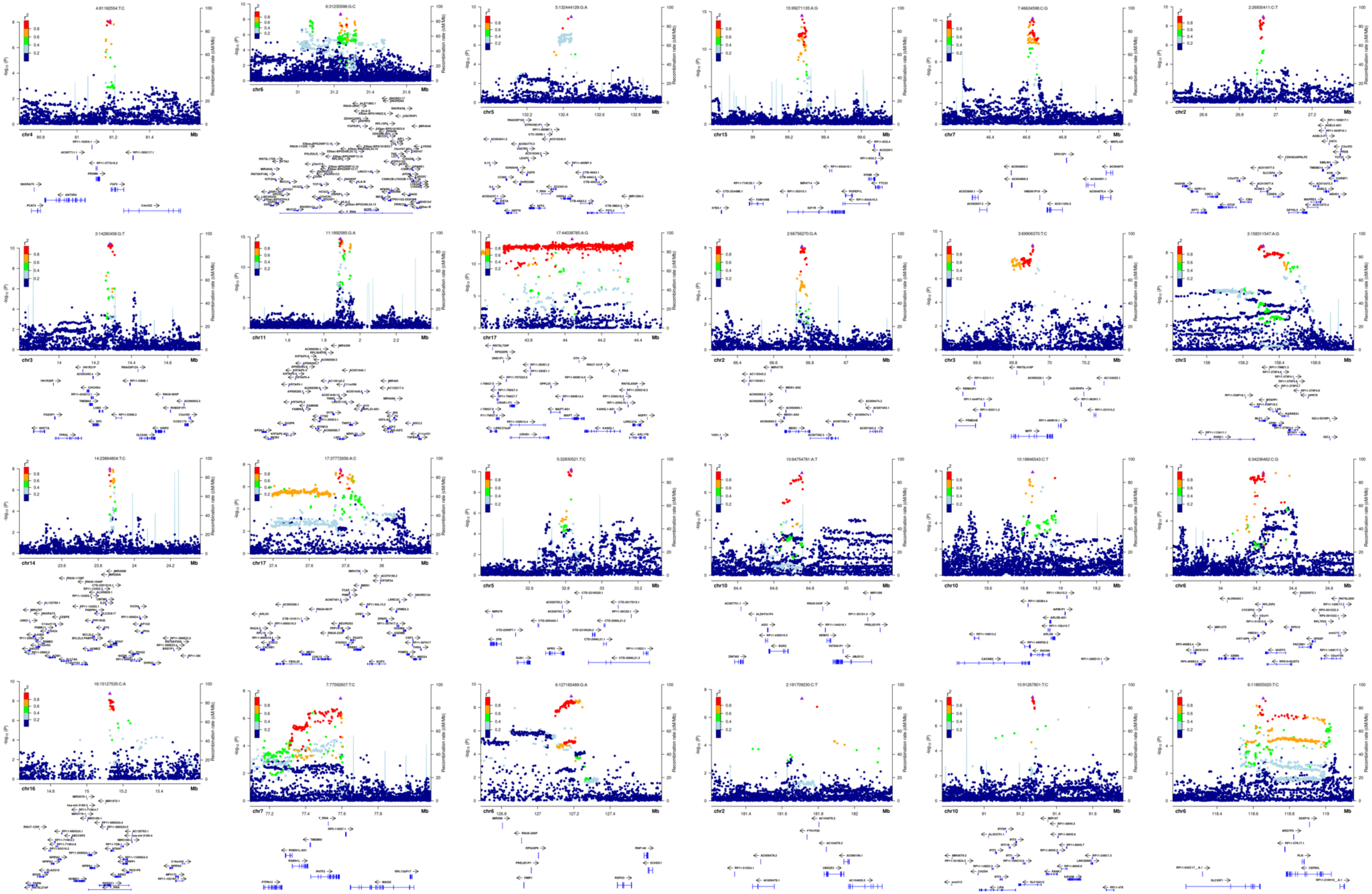

4:81182554:T:C
6:31235598:G:C
5:132444128:G:A
15:99271135:A:G
7:46634598:C:G
2:26930411:C:T
3:14280458:G:T
11:1892585:G:A
17:44038785:A:G
2:66756270:G:A
3:69906370:T:C
3:158311547:A:G
14:23864804:T:C
17:37772656:A:C
5:32830521:T:C
10:64754781:A:T
10:18846543:C:T
6:34236462:C:G
16:15127535:C:A
7:77592607:T:C
6:127185489:G:A
2:181709230:C:T
10:91267801:T:C
6:118655020:T:C

LVM

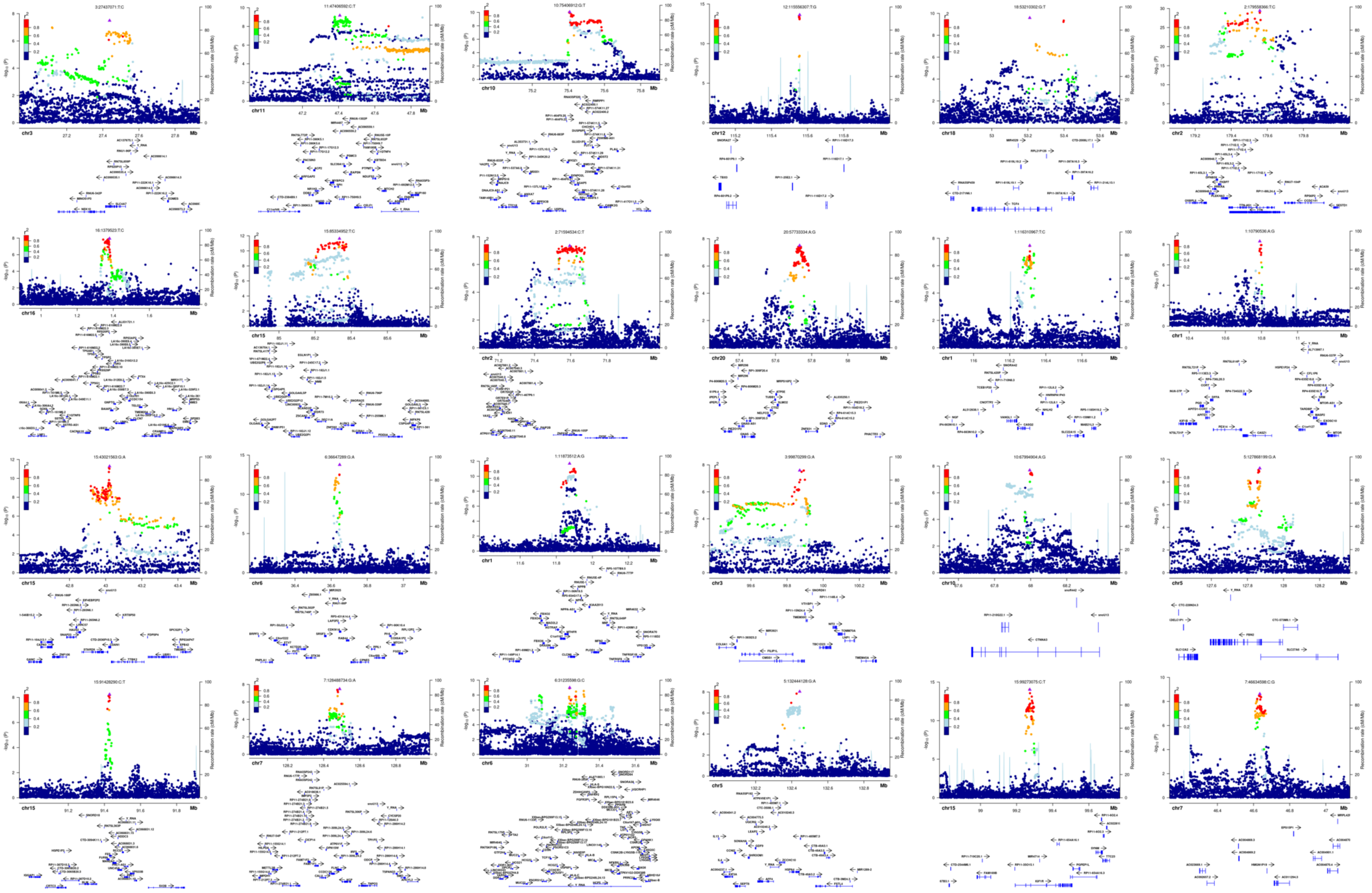

LVM

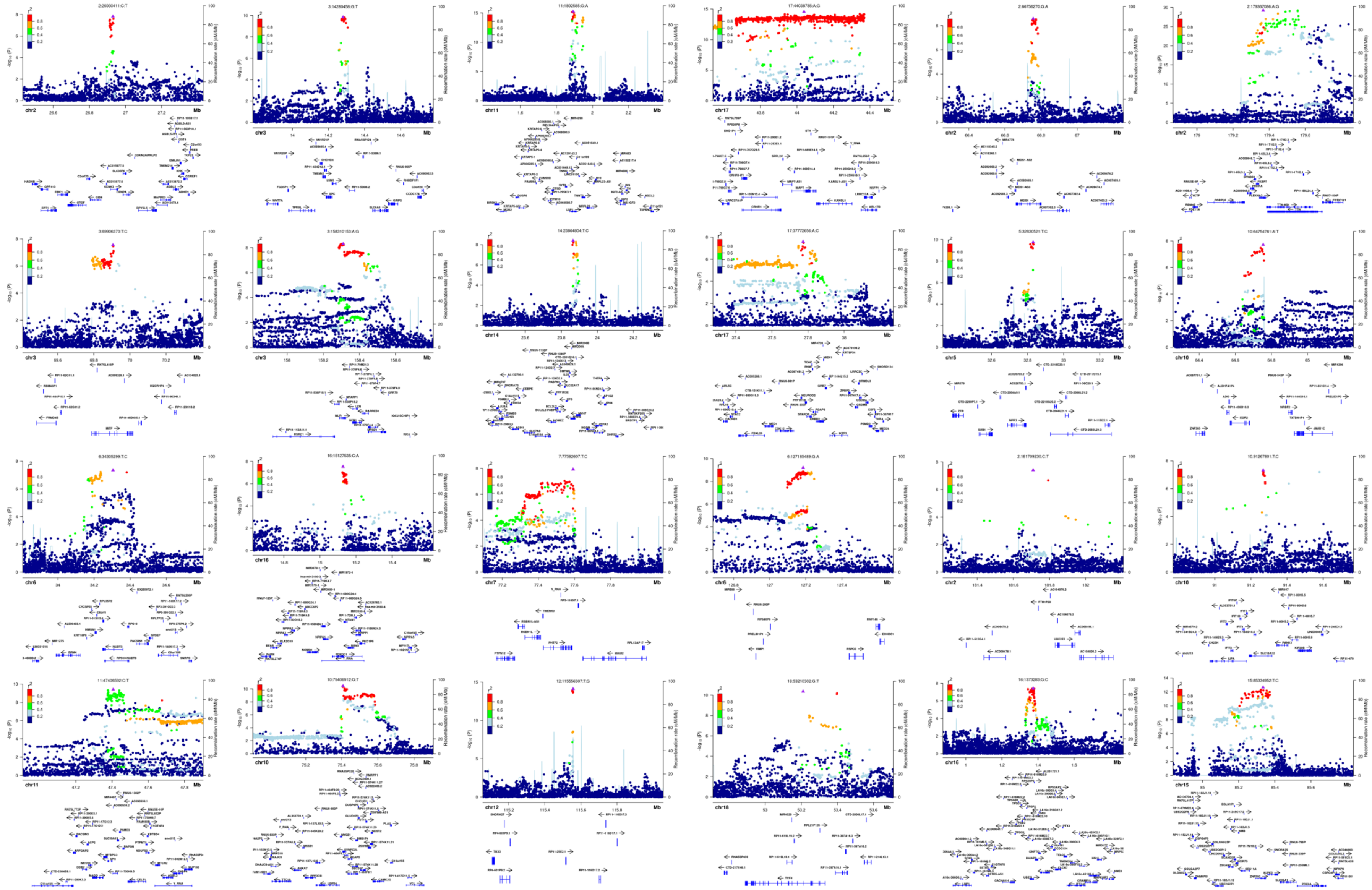

LVM_BSA

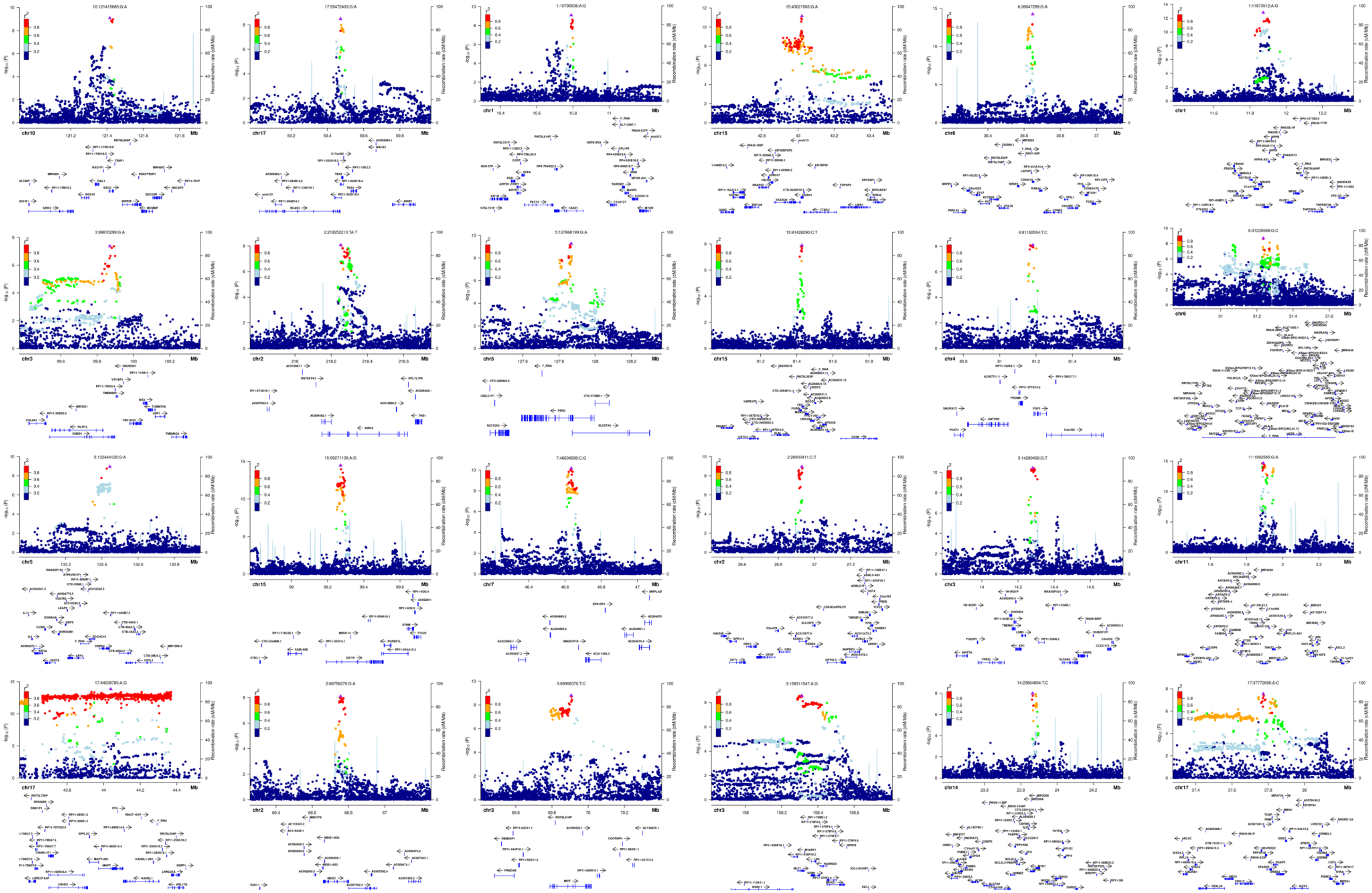
10:121415685:G:A
17:59472403:G:A
1:10790536:A:G
15:43021563:G:A
6:36647289:G:A
1:11873512:A:G
3:99870299:G:A
2:218252213:TA:T
5:127868199:G:A
15:91428290:C:T
4:81182554:T:C
6:31235598:G:C
5:132444128:G:A
15:99271135:A:G
7:46634598:C:G
2:26930411:C:T
3:14280456:G:T
11:1892585:G:A
17:44038785:A:G
2:66756270:G:A
3:69906370:T:C
3:158311547:A:G
14:23864804:T:C
17:37772656:A:C

LVM_BSA

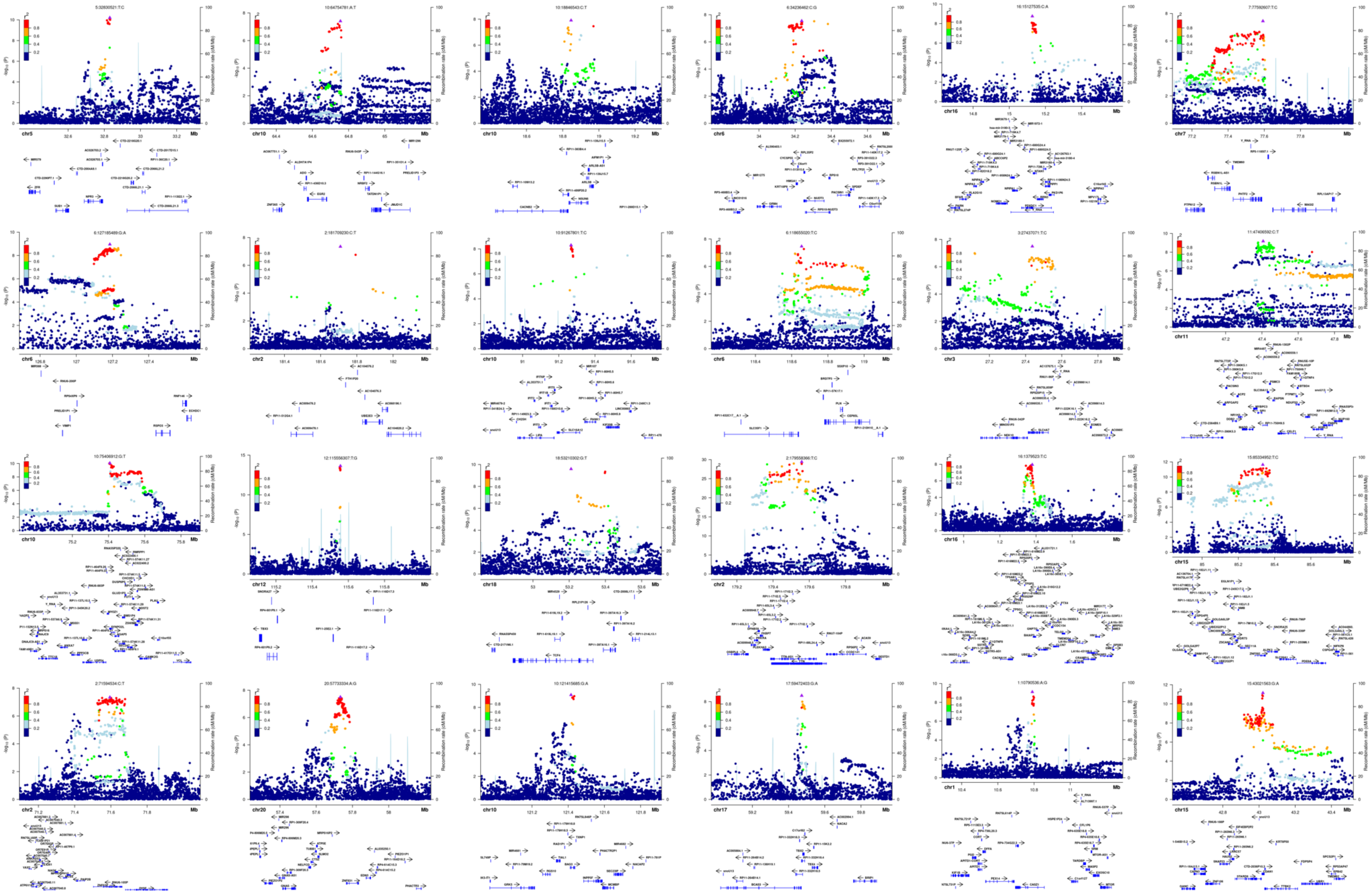

5:32830521:T:C
10:64754781:A:T
10:18846543:C:T
6:34236462:C:G
16:15127535:C:A
7:77592607:T:C
6:127185489:G:A
2:181709230:C:T
10:91267801:T:C
6:118655020:T:C
3:27437071:T:C
11:47406592:C:T
10:75406912:G:T
12:115556307:T:G
18:53210302:G:T
2:179558366:T:C
16:1379523:T:C
15:85334952:T:C
2:71594534:C:T
20:57733334:A:G
10:121415685:G:A
17:59472403:G:A
1:10790536:A:G
15:43021563:G:A

LVM_BSA

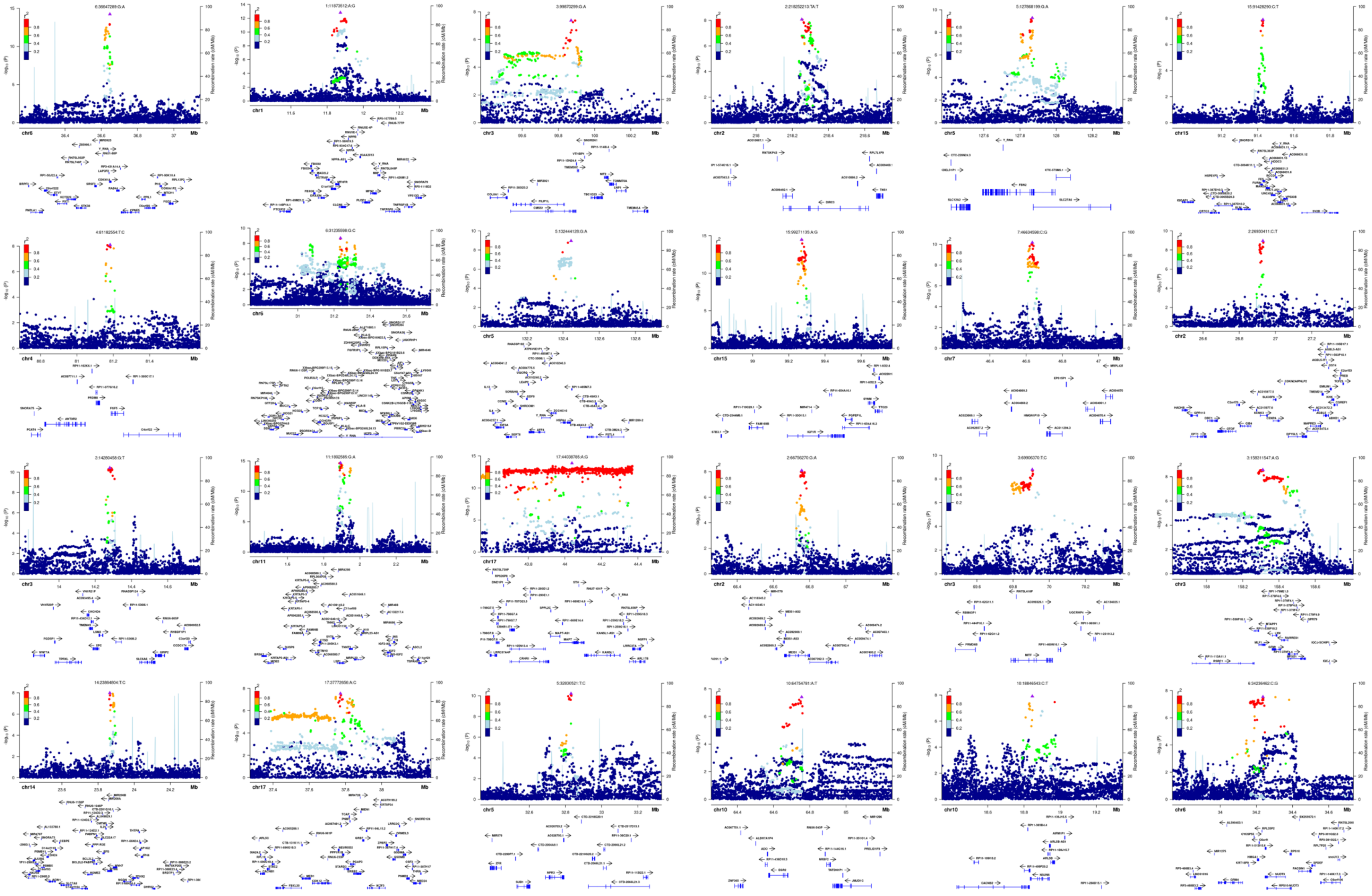

LVM_BSA

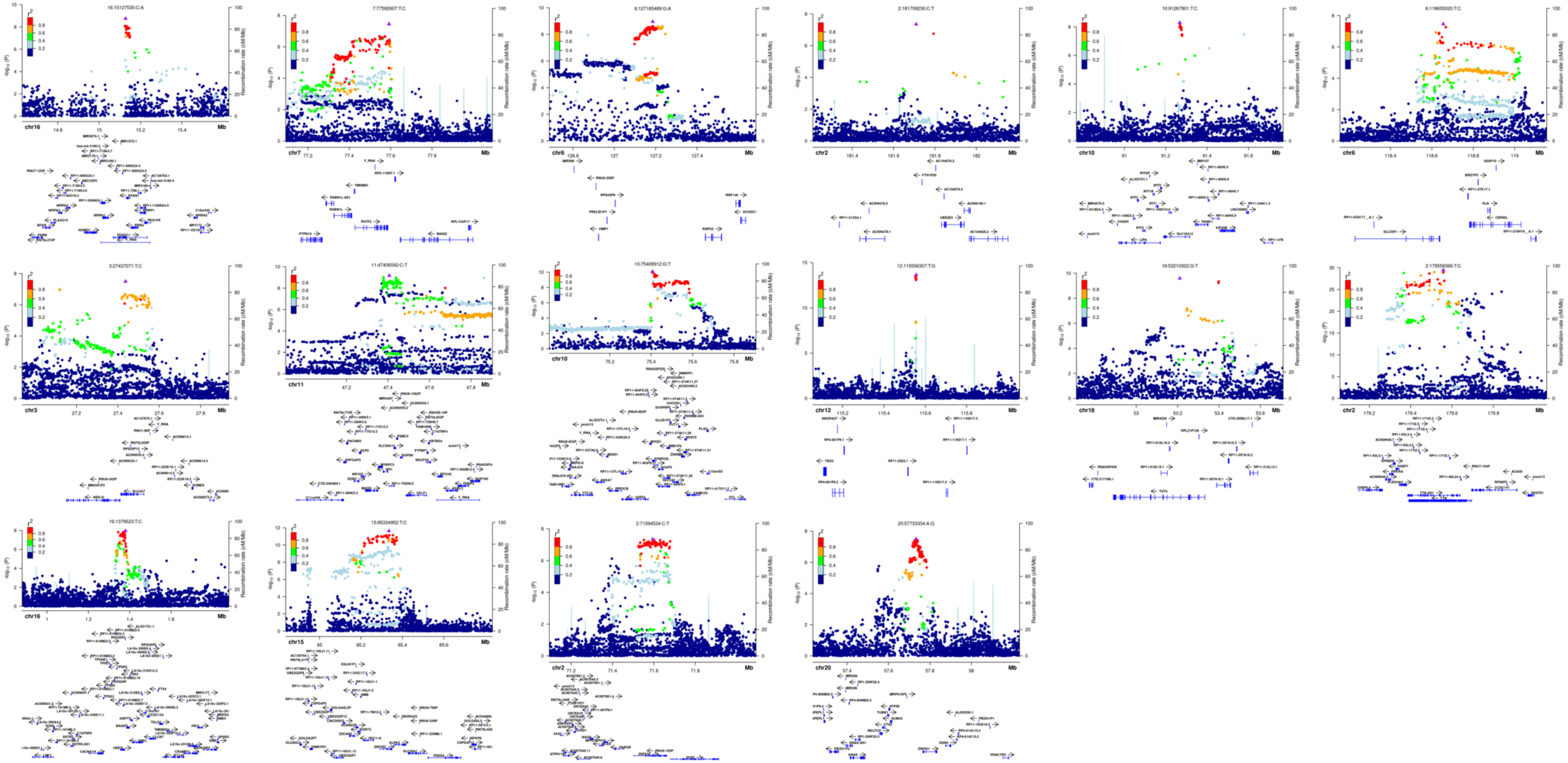
16:15127535:C:A
7:77592607:T:C
6:127185489:G:A
2:181709230:C:T
10:91267801:T:C
6:118655020:T:C
3:27437071:T:C
11:47406592:C:T
10:75406912:G:T
12:115556307:T:G
18:53210302:G:T
2:179558366:T:C
16:1379523:T:C
15:85334952:T:C
2:71594534:C:T
20:57733334:A:G

LVMCF

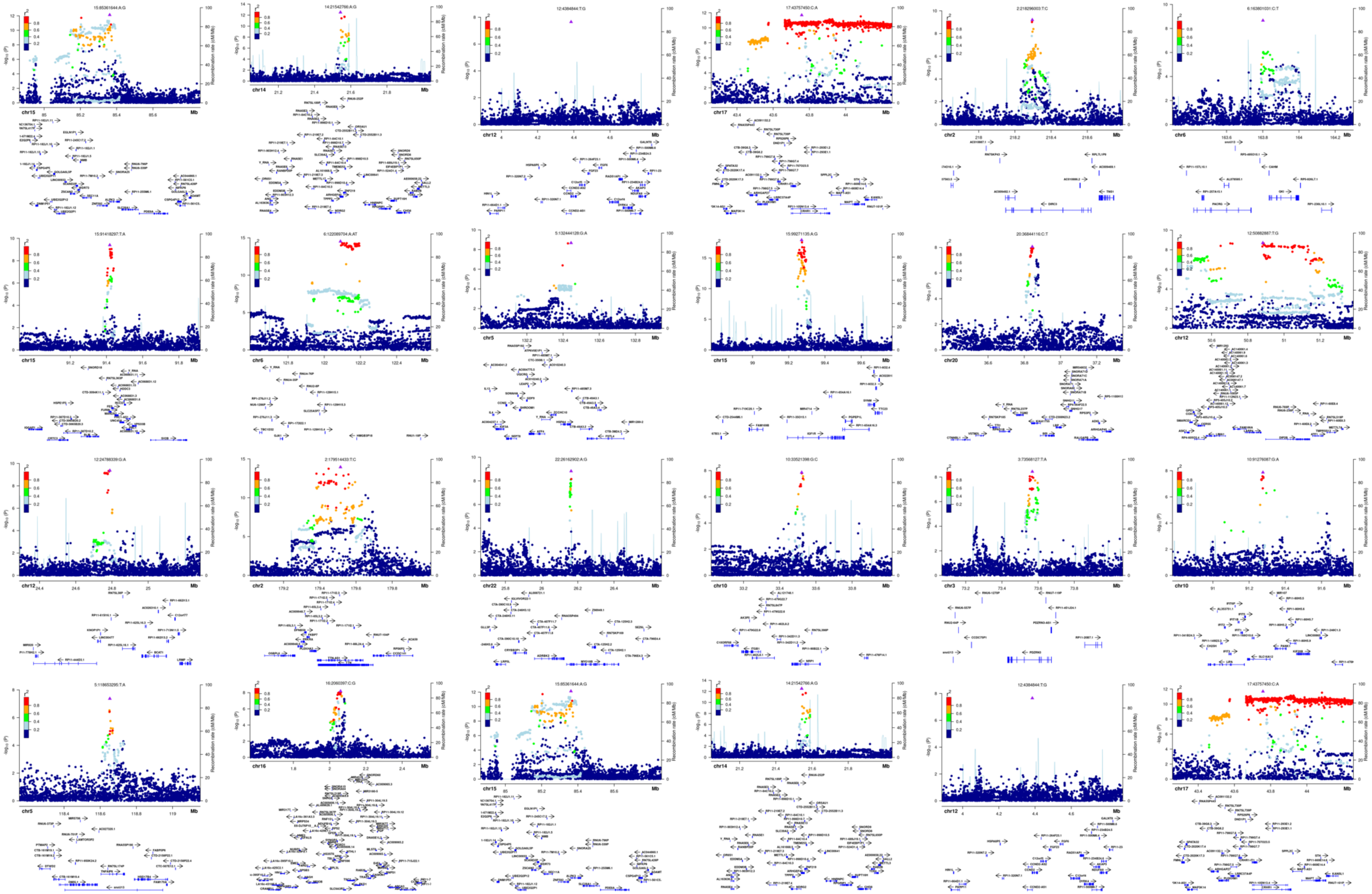

LVMCF

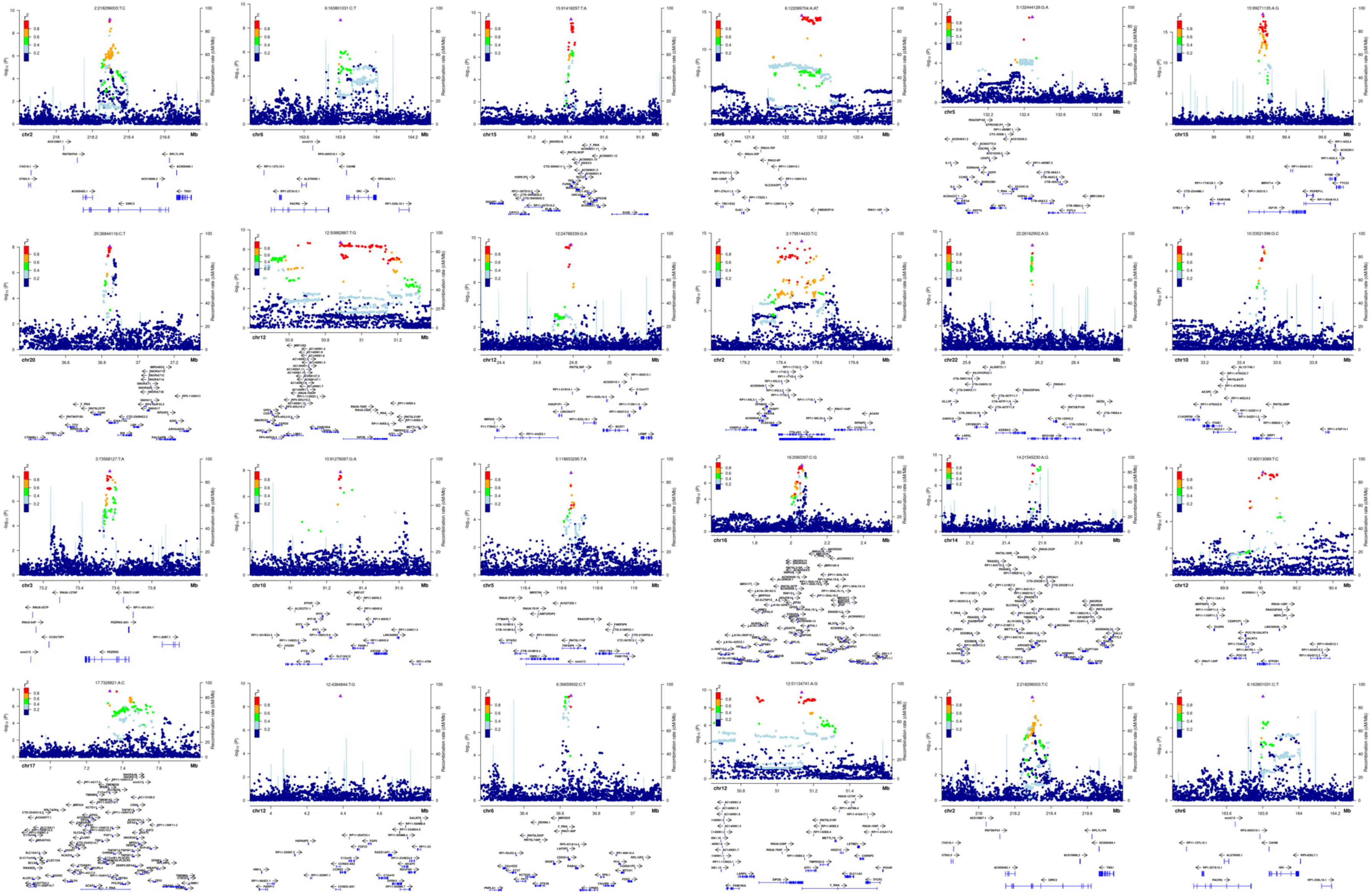

2:218296003:T:C
6:163801031:C:T
15:91418297:T:A
6:122089704:A:AT
5:132444128:G:A
15:99271135:A:G
20:36844116:C:T
12:50862887:T:G
12:24788339:G:A
2:179514433:T:C
22:26162902:A:G
10:33521398:G:C
3:73568127:T:A
10:91276087:G:A
5:118653295:T:A
16:2060397:C:G
14:21545230:A:G
12:90013089:T:C
17:7328821:A:C
12:4384844:T:G
6:36659932:C:T
12:51134741:A:G
2:218296003:T:C
6:163801031:C:T

LVMCF

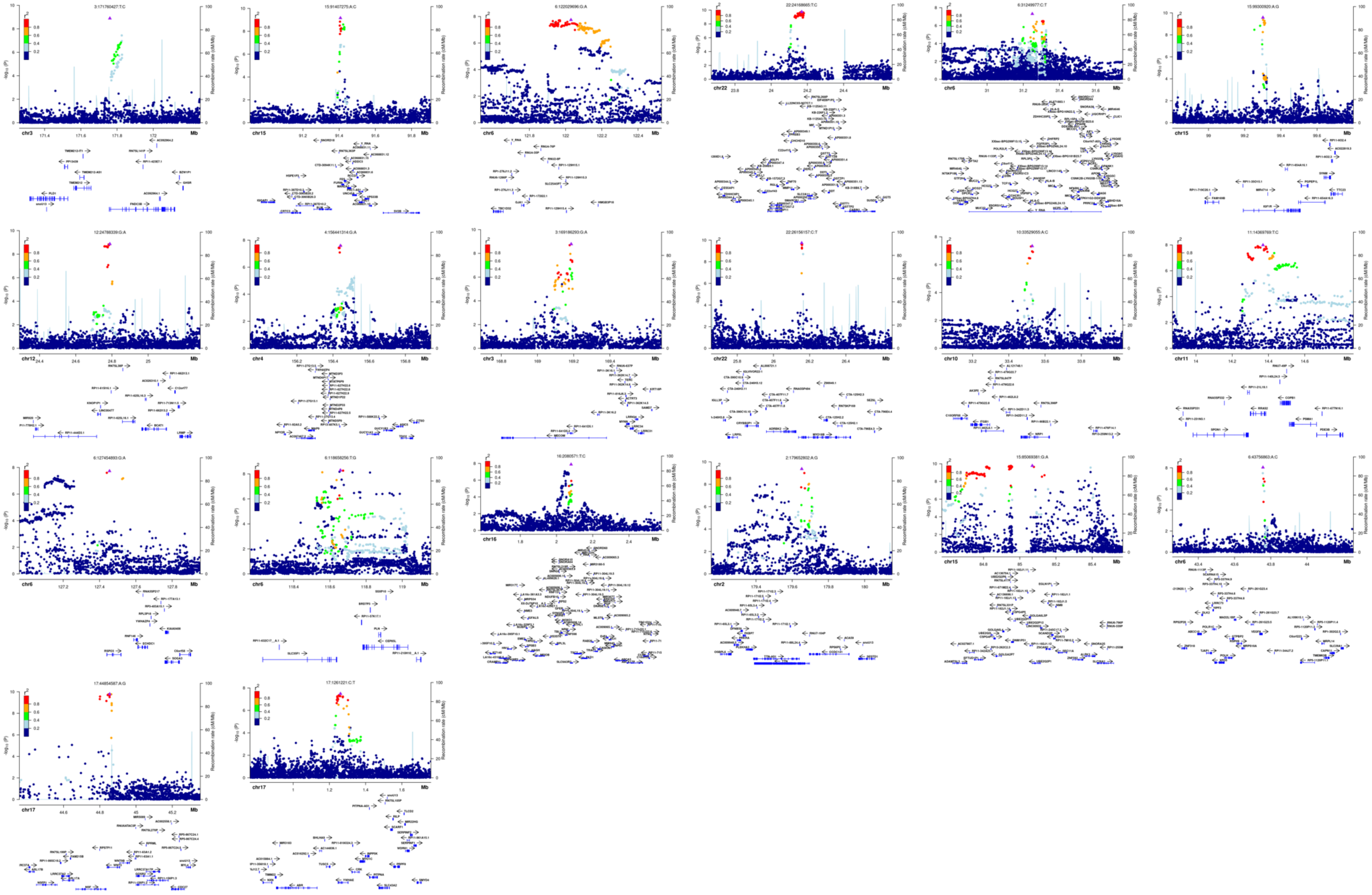

LVMVR

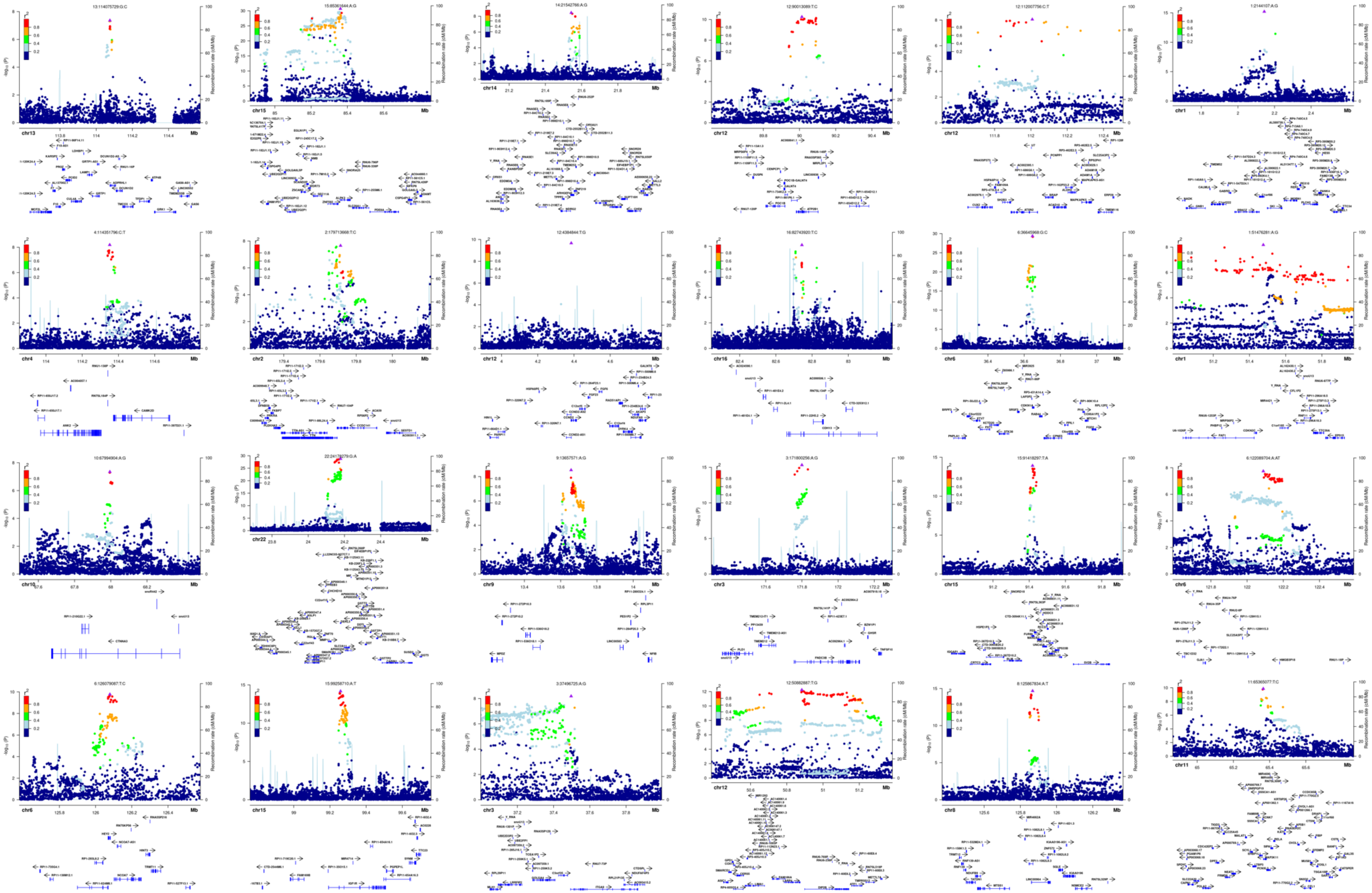

LVMVR

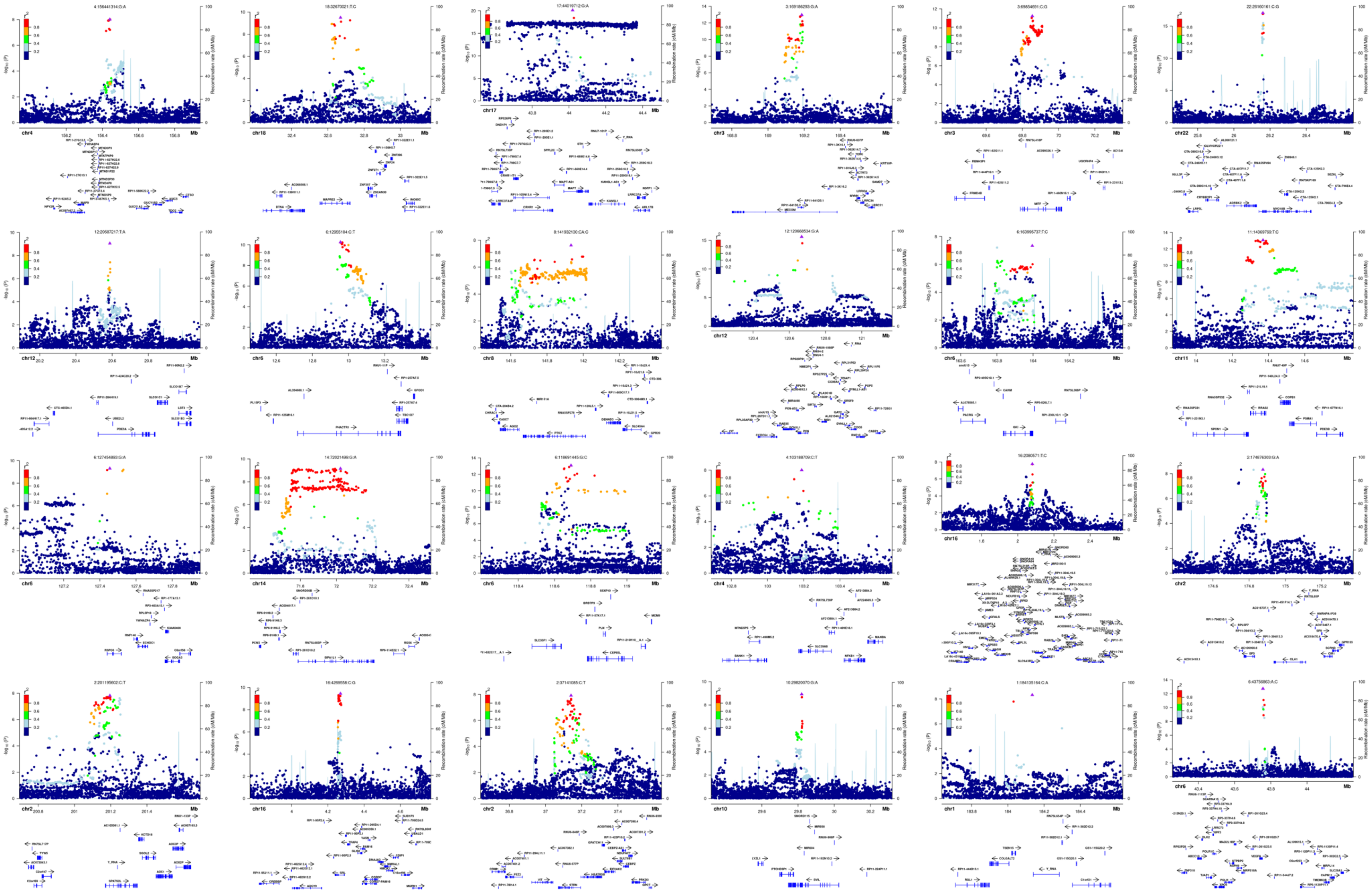

LVMVR

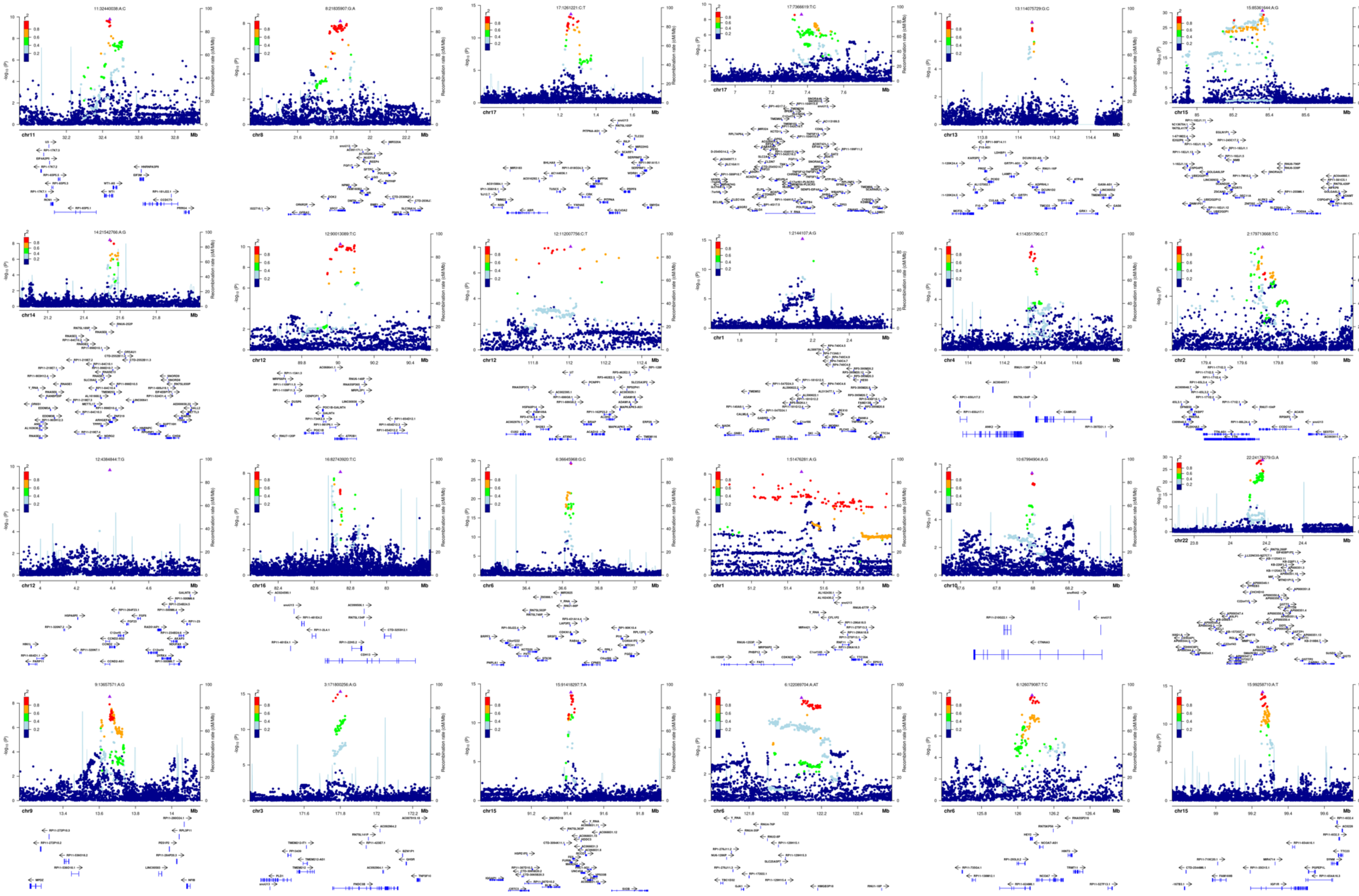

LVMVR

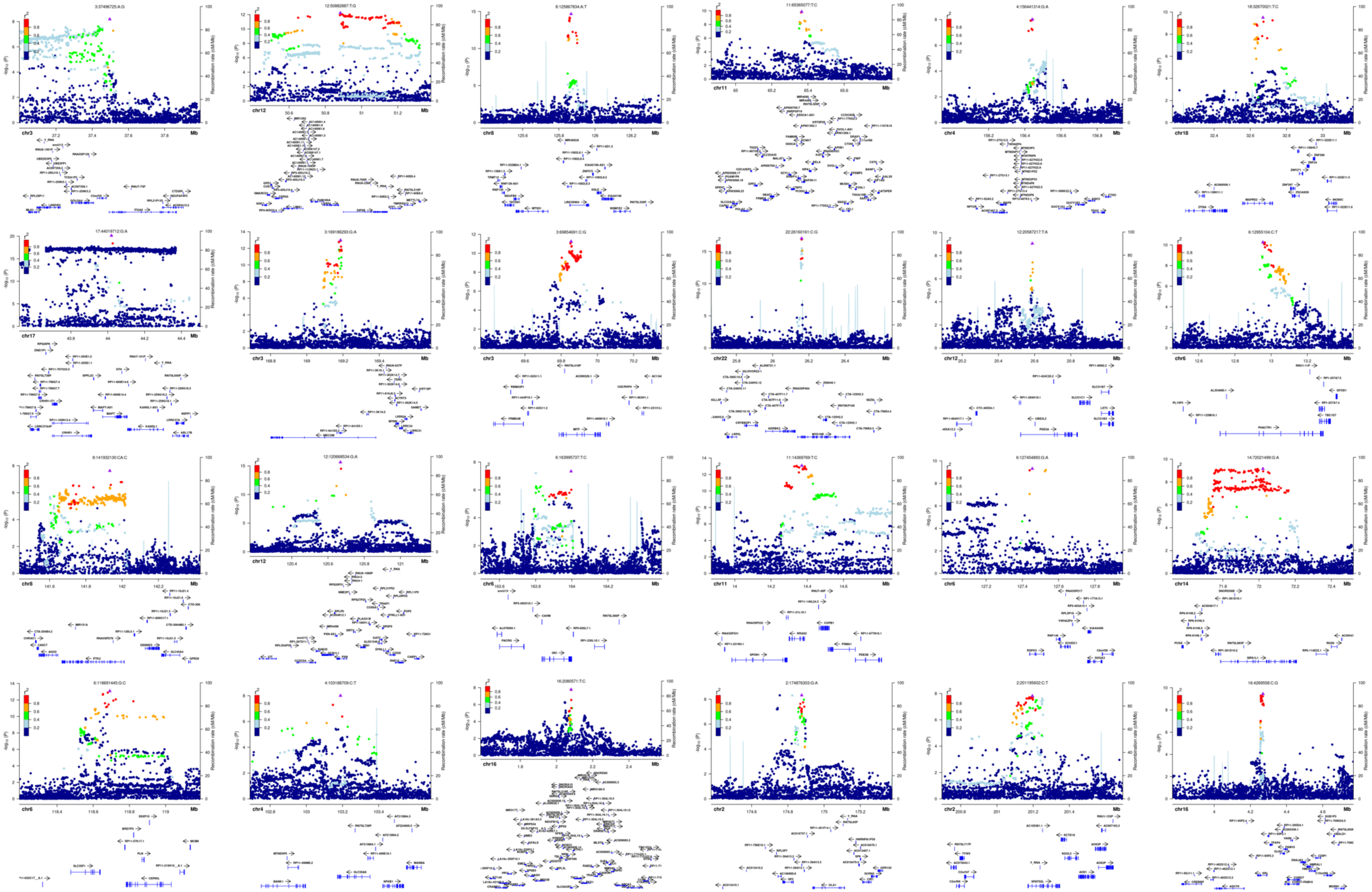

LVMVR

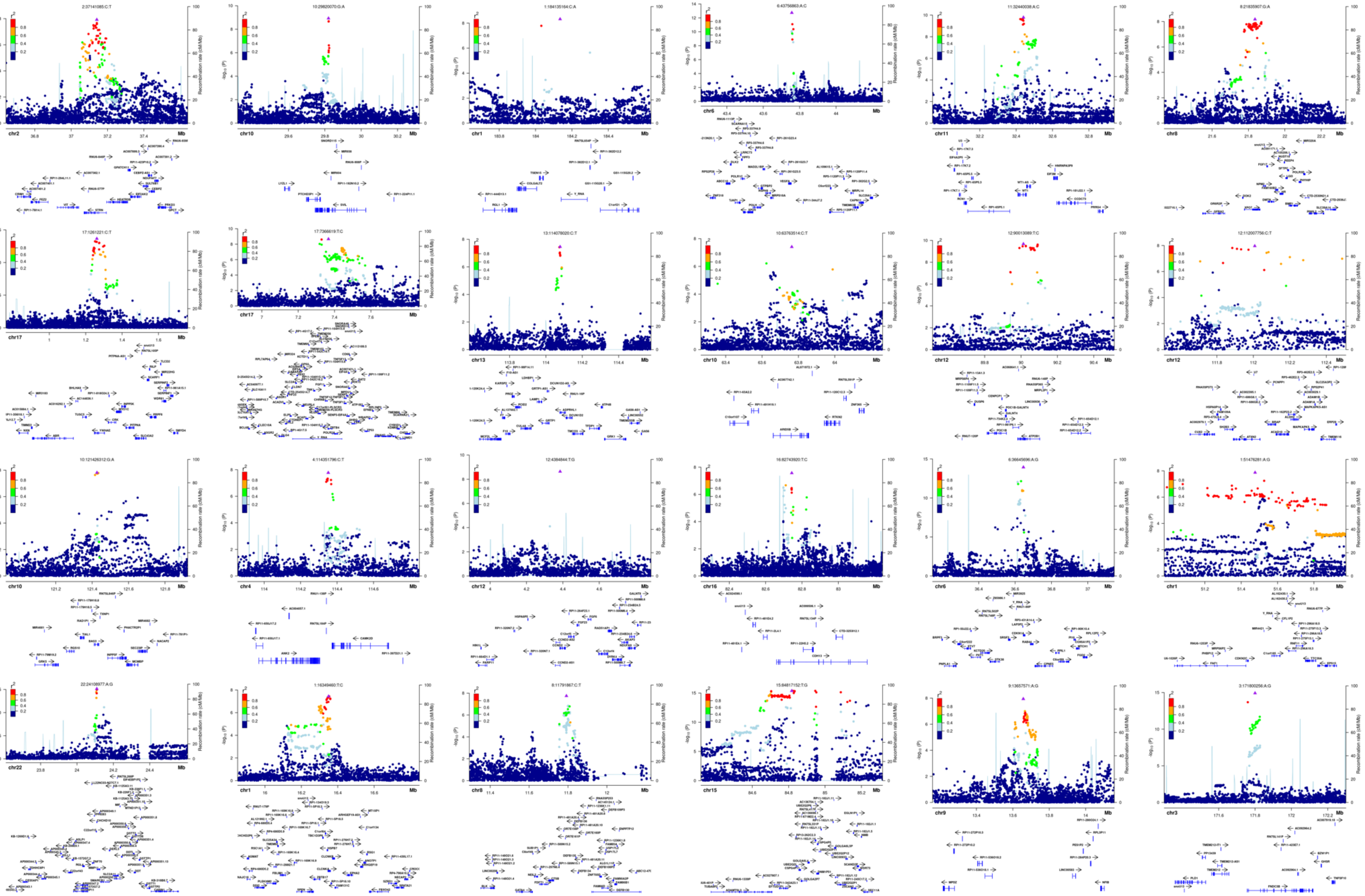

LVMVR

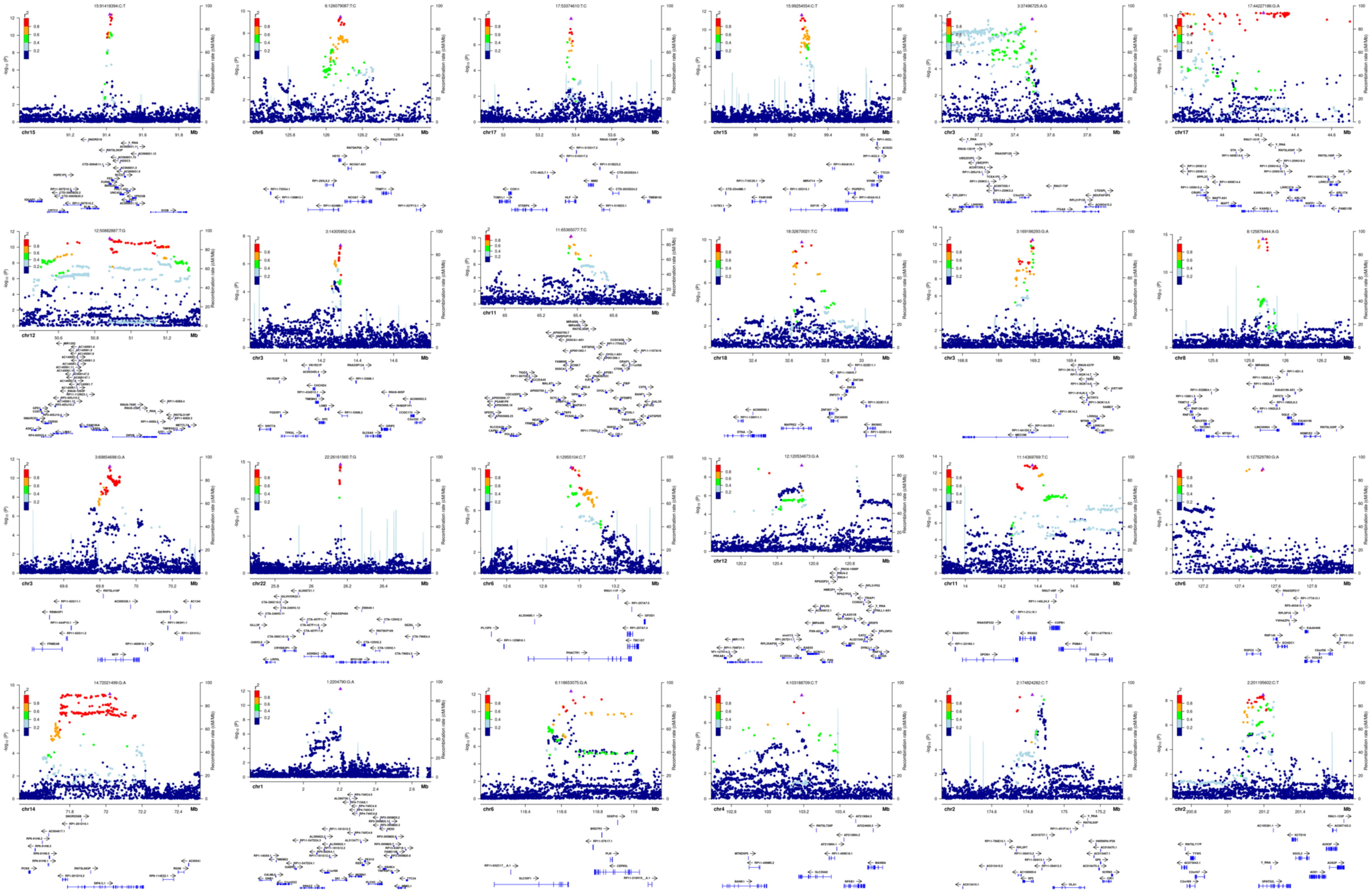
15:91418394:C:T
6:126079087:T:C
17:53374610:T:C
15:99254554:C:T
3:37496725:A:G
17:44227186:G:A
12:50882887:T:G
3:14305952:G:A
11:65365077:T:C
18:32670021:T:C
3:169186293:G:A
8:125876444:A:G
3:69854698:G:A
22:26161565:T:G
6:12955104:C:T
12:120534673:G:A
11:14369769:T:C
6:127529780:G:A
14:72021499:G:A
1:2204790:G:A
6:118653075:G:A
4:103188709:C:T
2:174824282:C:T
2:201195602:C:T

LVMVR

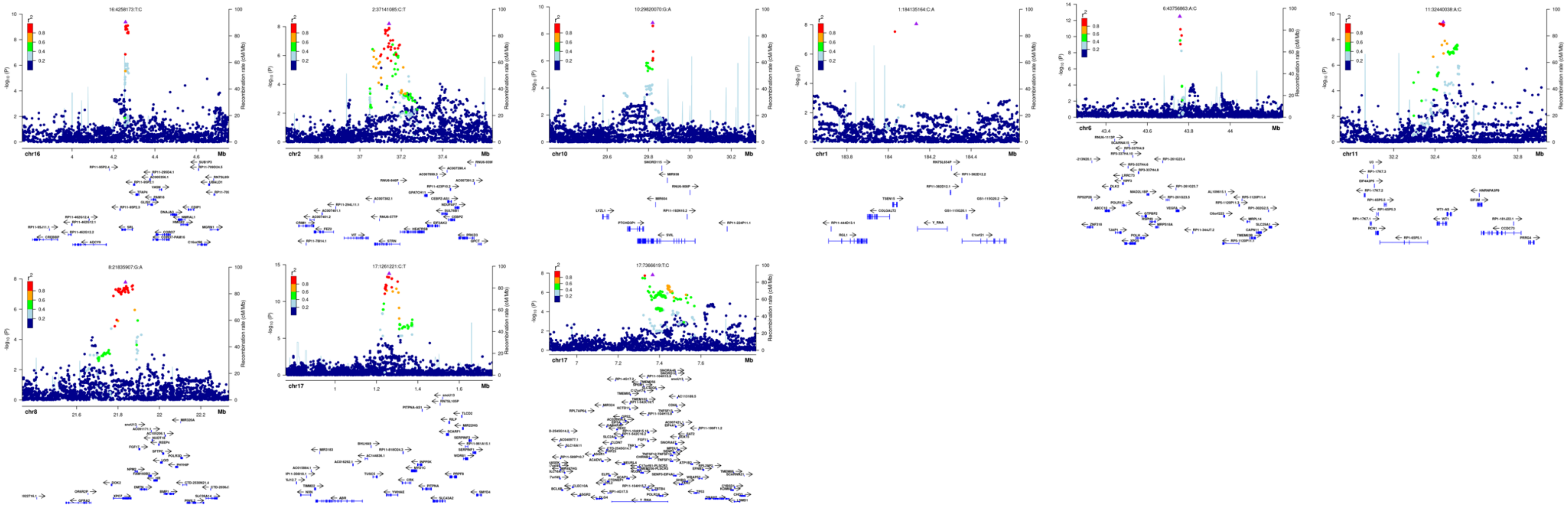

16:4258173:T:C
2:37141085:C:T
10:29820070:G:A
1:184135164:C:A
6:43756863:A:C
11:32440038:A:C
8:21835907:G:A
17:1261221:C:T
17:7366619:T:C

LVSV

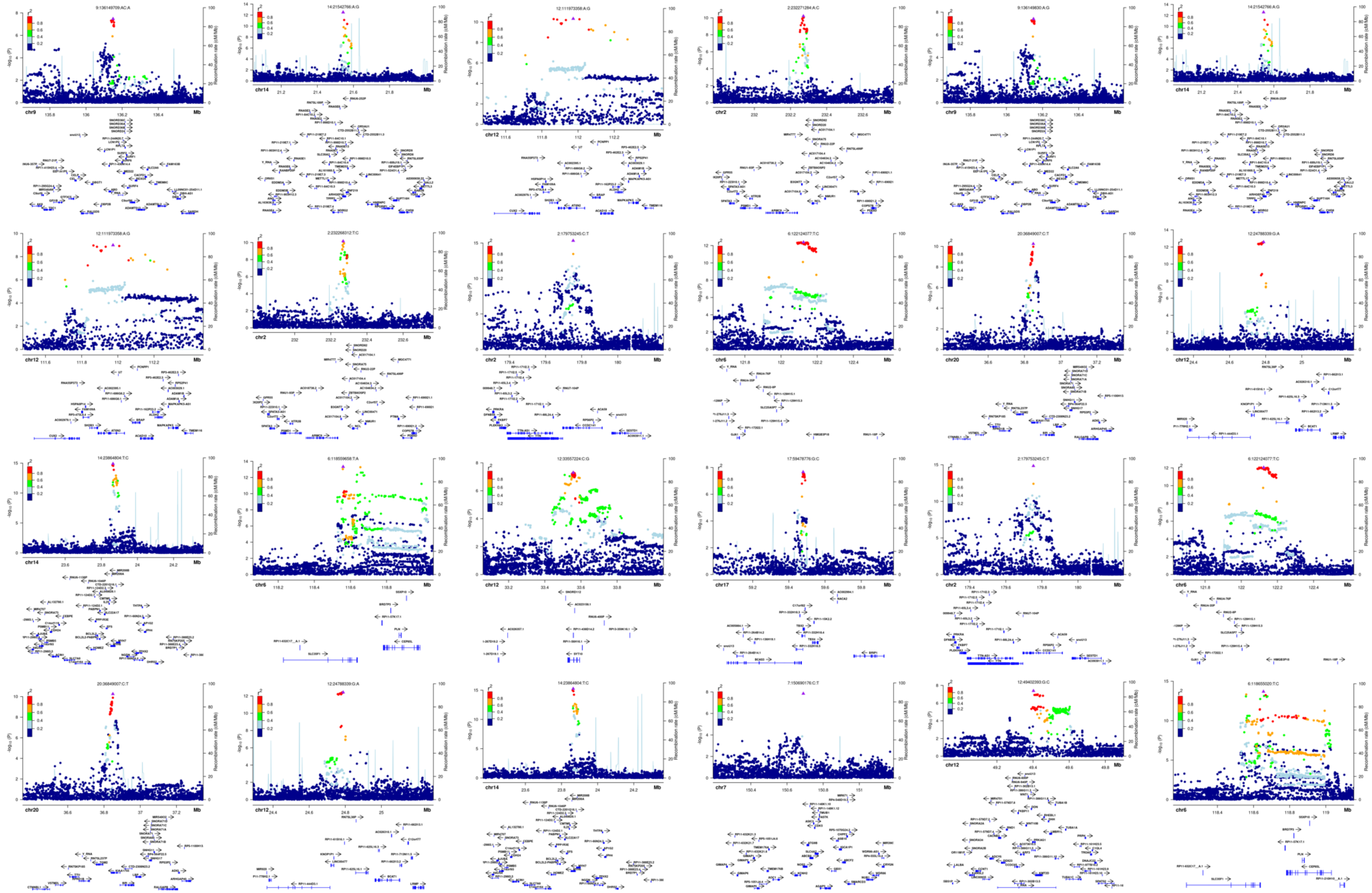

LVSV

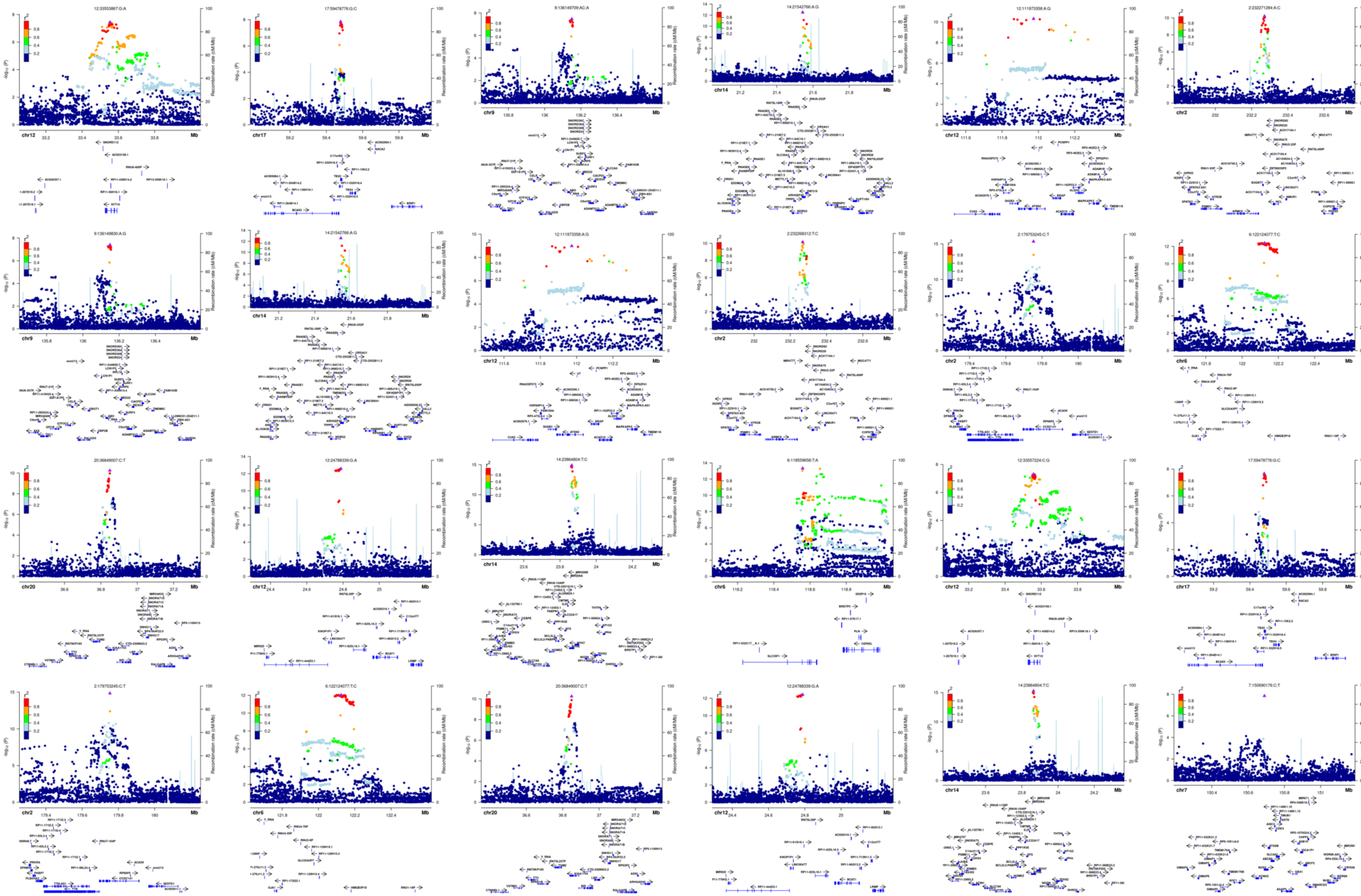
12:33553867:G:A
17:59478776:G:C
9:136149709:AC:A
14:21542766:A:G
12:111973358:A:G
2:232271284:A:C
9:136149830:A:G
14:21542766:A:G
12:111973358:A:G
2:232268312:T:C
2:179753245:C:T
6:122124077:T:C
20:36849007:C:T
12:24788339:G:A
14:23864804:T:C
6:118559658:T:A
12:33557224:C:G
17:59478776:G:C
2:179753245:C:T
6:122124077:T:C
20:36849007:C:T
12:24788339:G:A
14:23864804:T:C
7:150690176:C:T

LVSV

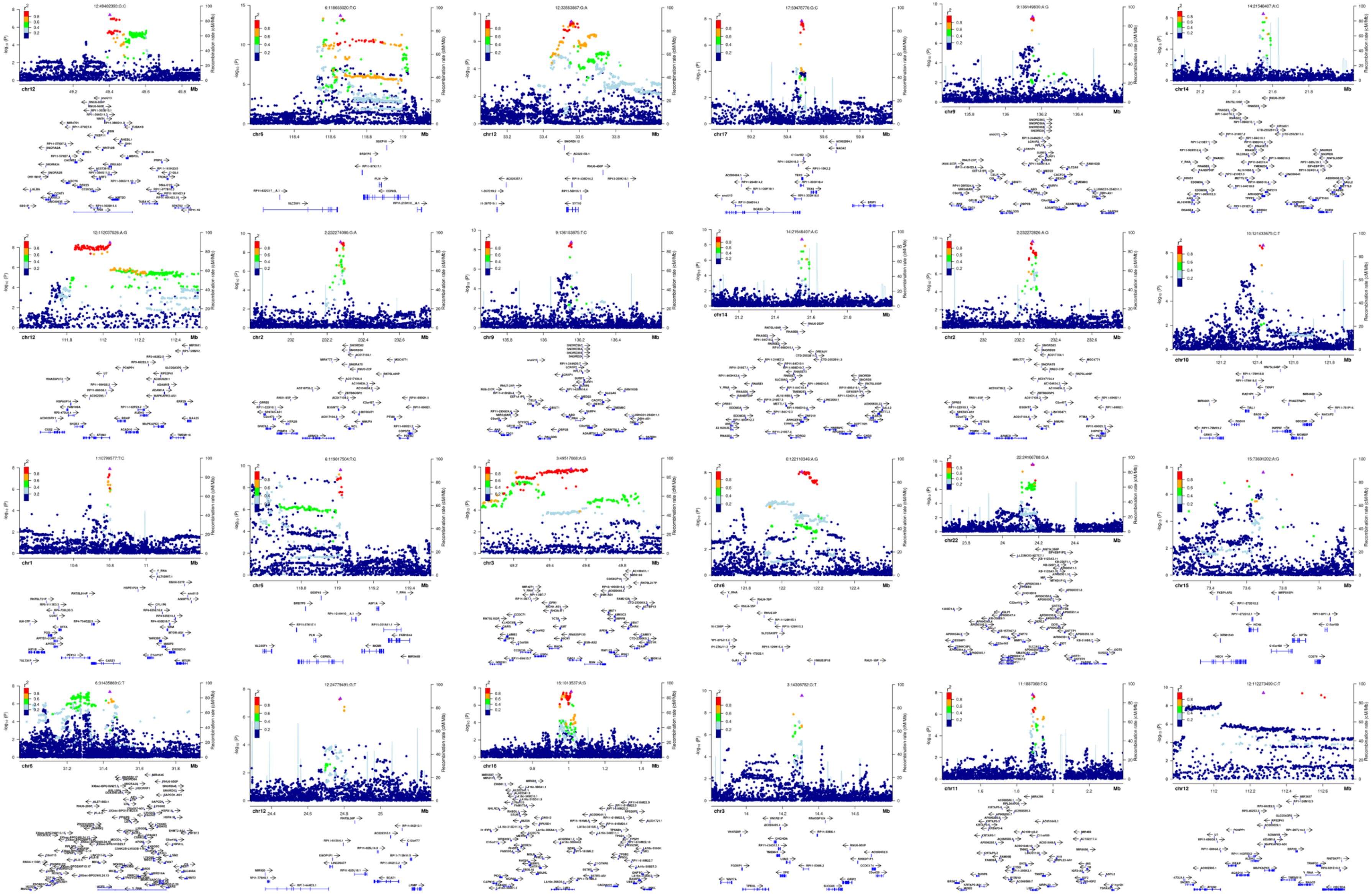

LVSV

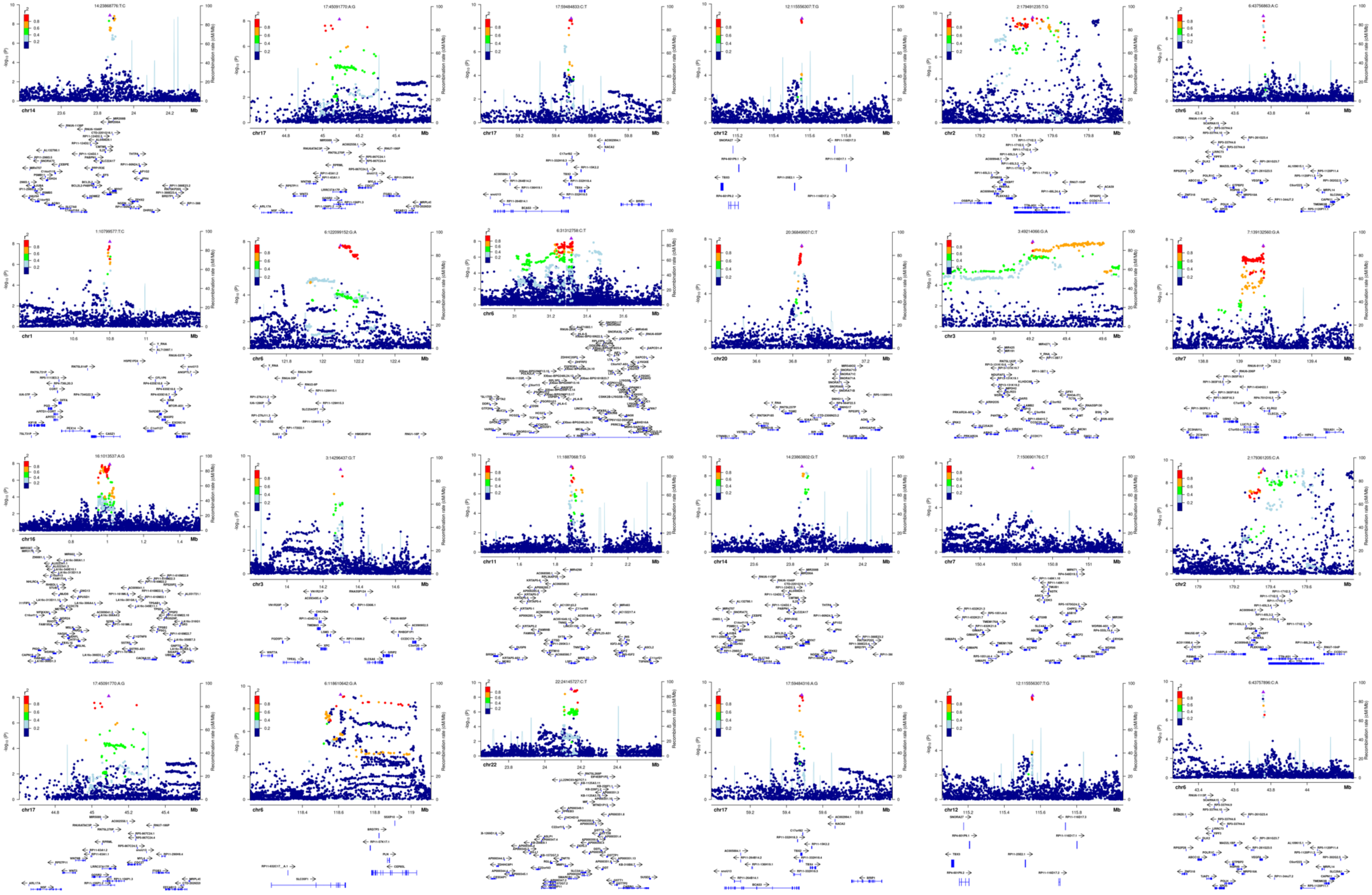

LVSV_BSA

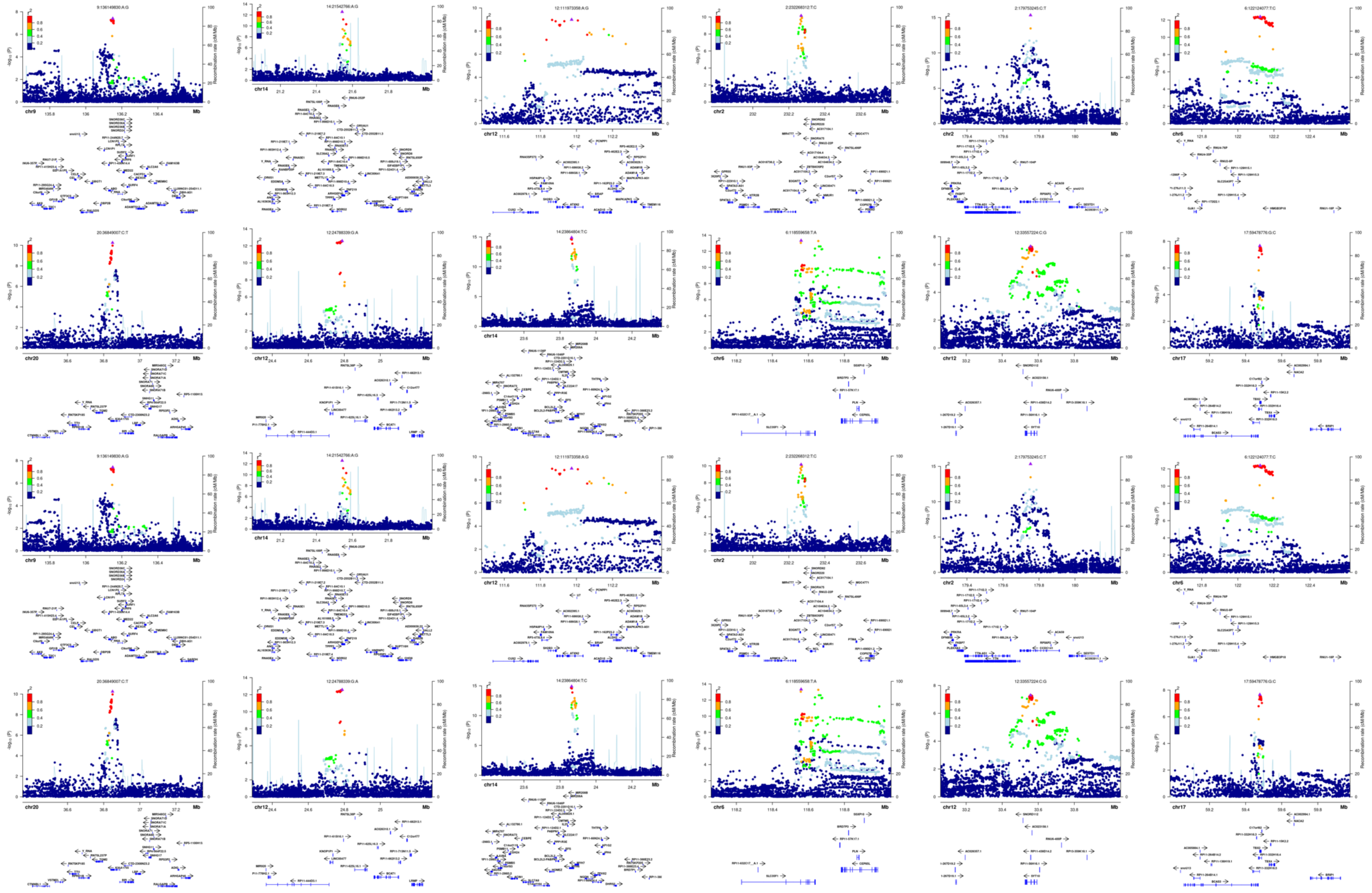

LVSV_BSA

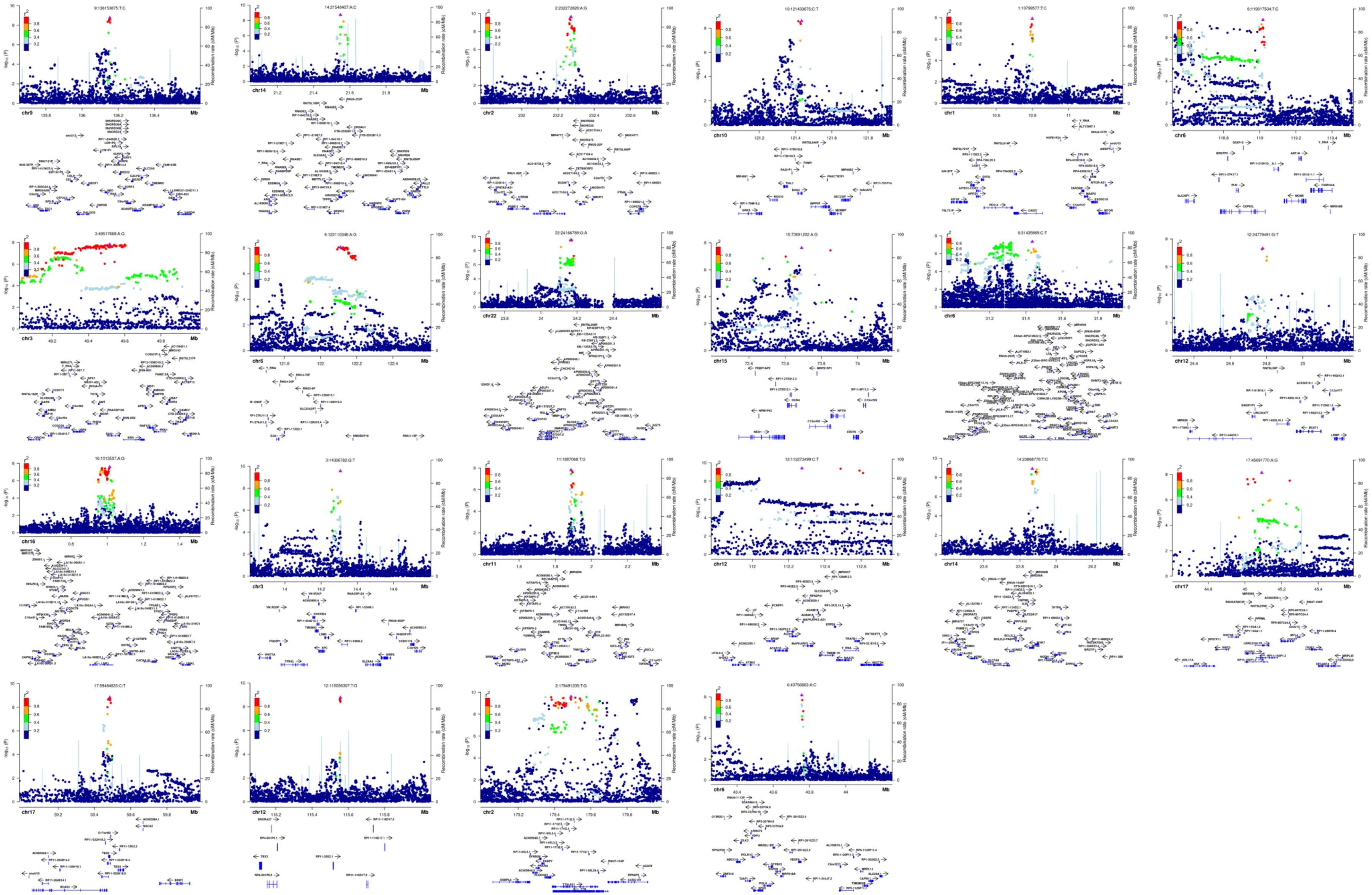

RV_LV_ratio

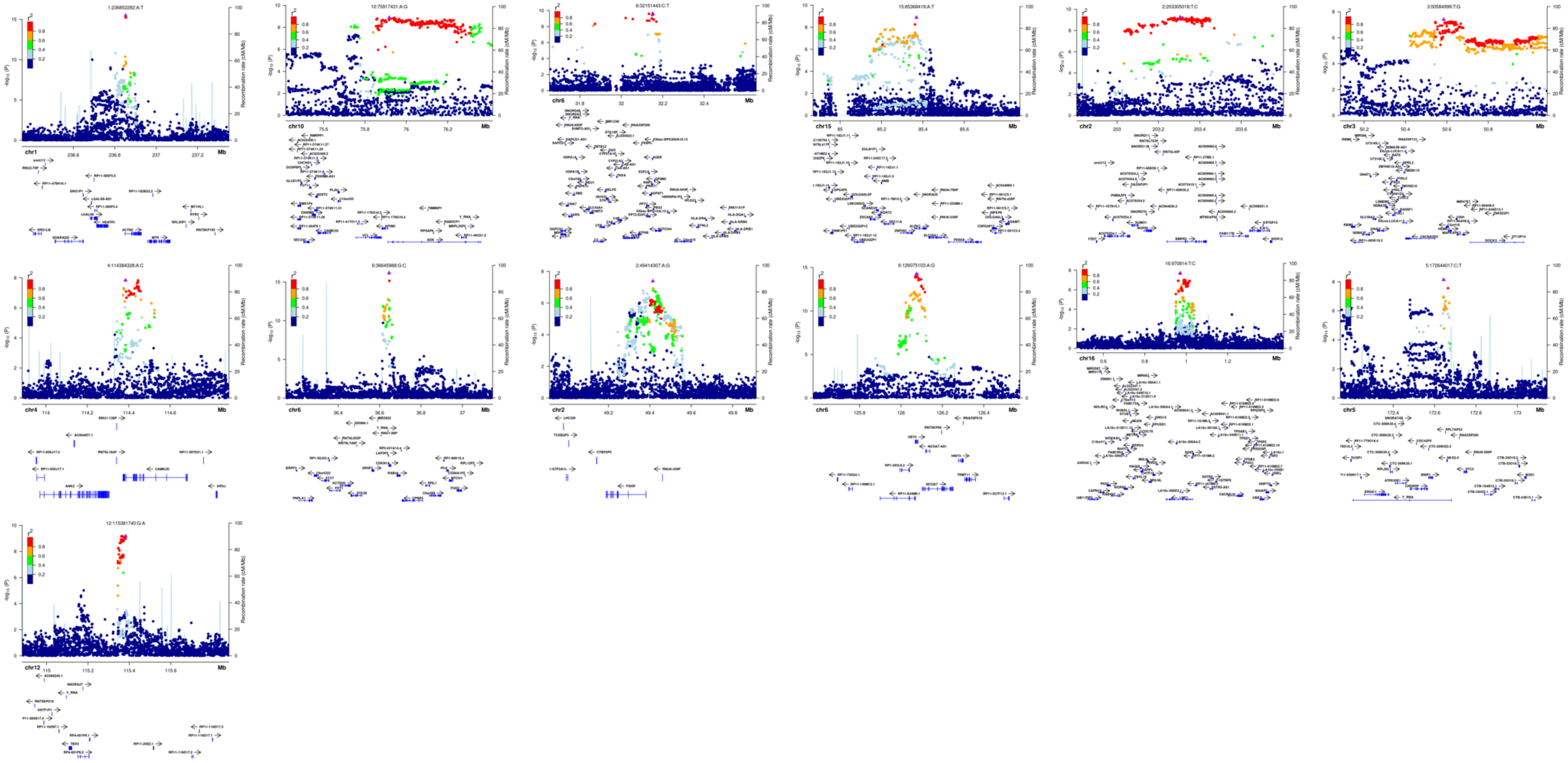

1:236852282:A:T
10:75917431:A:G
6:32151443:C:T
15:85368419:A:T
2:203305018:T:C
3:50584999:T:G
4:114384328:A:C
6:36645988:G:C
2:49414307:A:G
6:126075103:A:G
16:970814:T:C
5:172644017:C:T
12:115381740:G:A

RVEDV

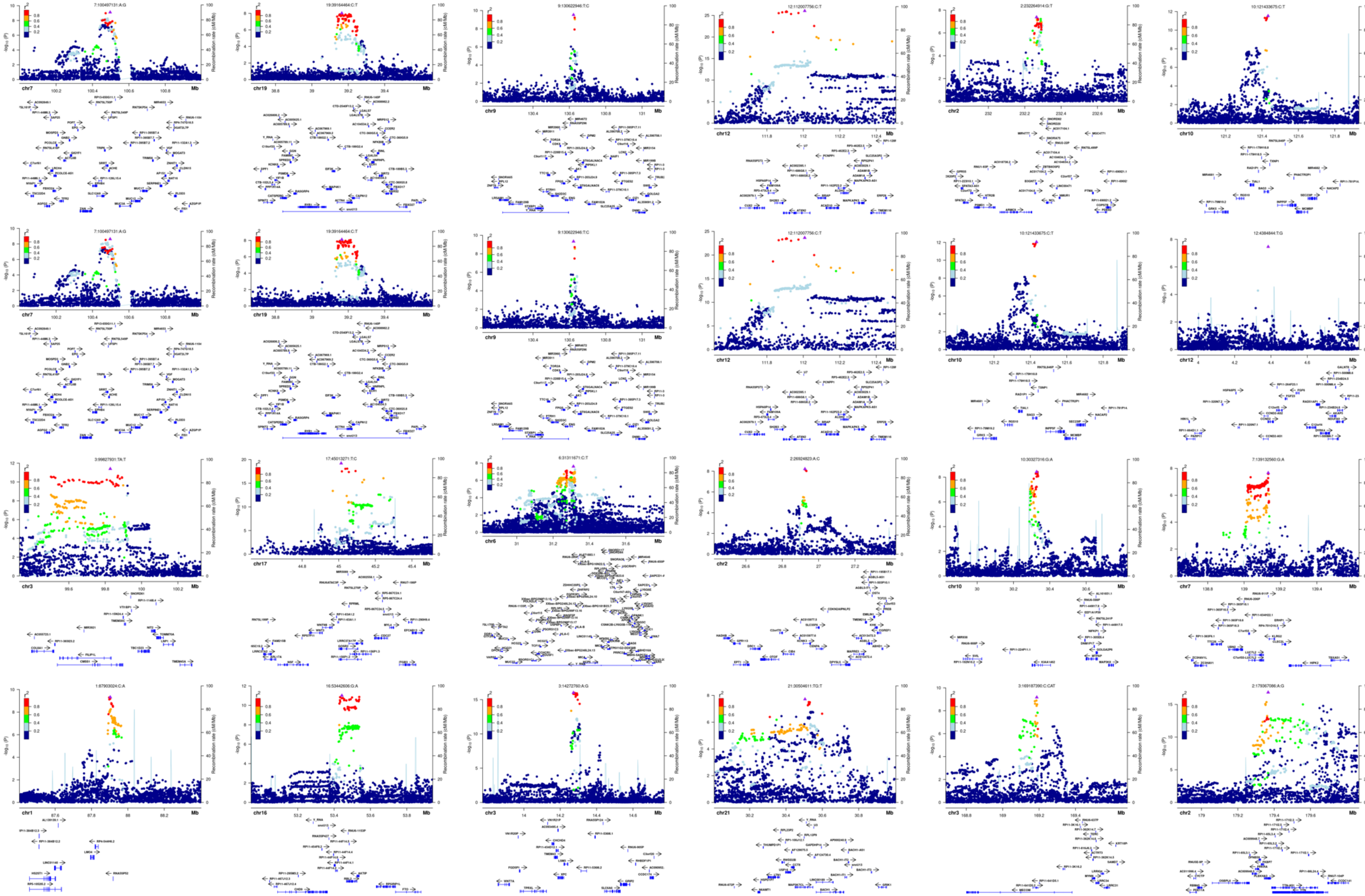
7:100497131:A:G
19:39164464:C:T
9:130622946:T:C
12:112007756:C:T
2:232264914:G:T
10:121433675:C:T
7:100497131:A:G
19:39164464:C:T
9:130622946:T:C
12:112007756:C:T
10:121433675:C:T
12:4384844:T:G
3:99827931:TA:T
17:45013271:T:C
6:31311671:C:T
2:26924823:A:C
10:30327316:G:A
7:139132560:G:A
1:87903024:C:A
16:53442606:G:A
3:14272760:A:G
21:30504611:TG:T
3:169187390:C:CAT
2:179367086:A:G

RVEDV

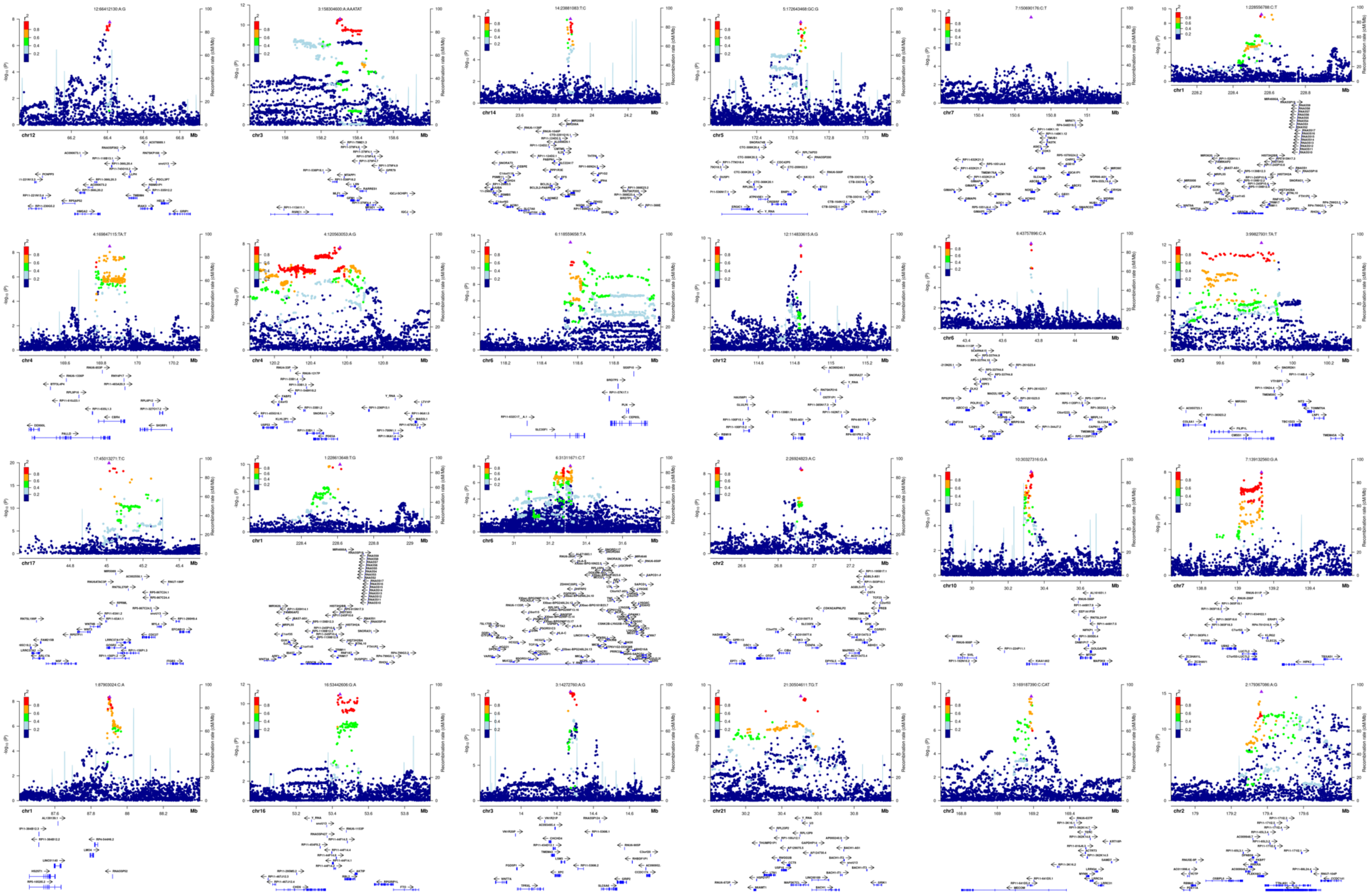

RVEDV

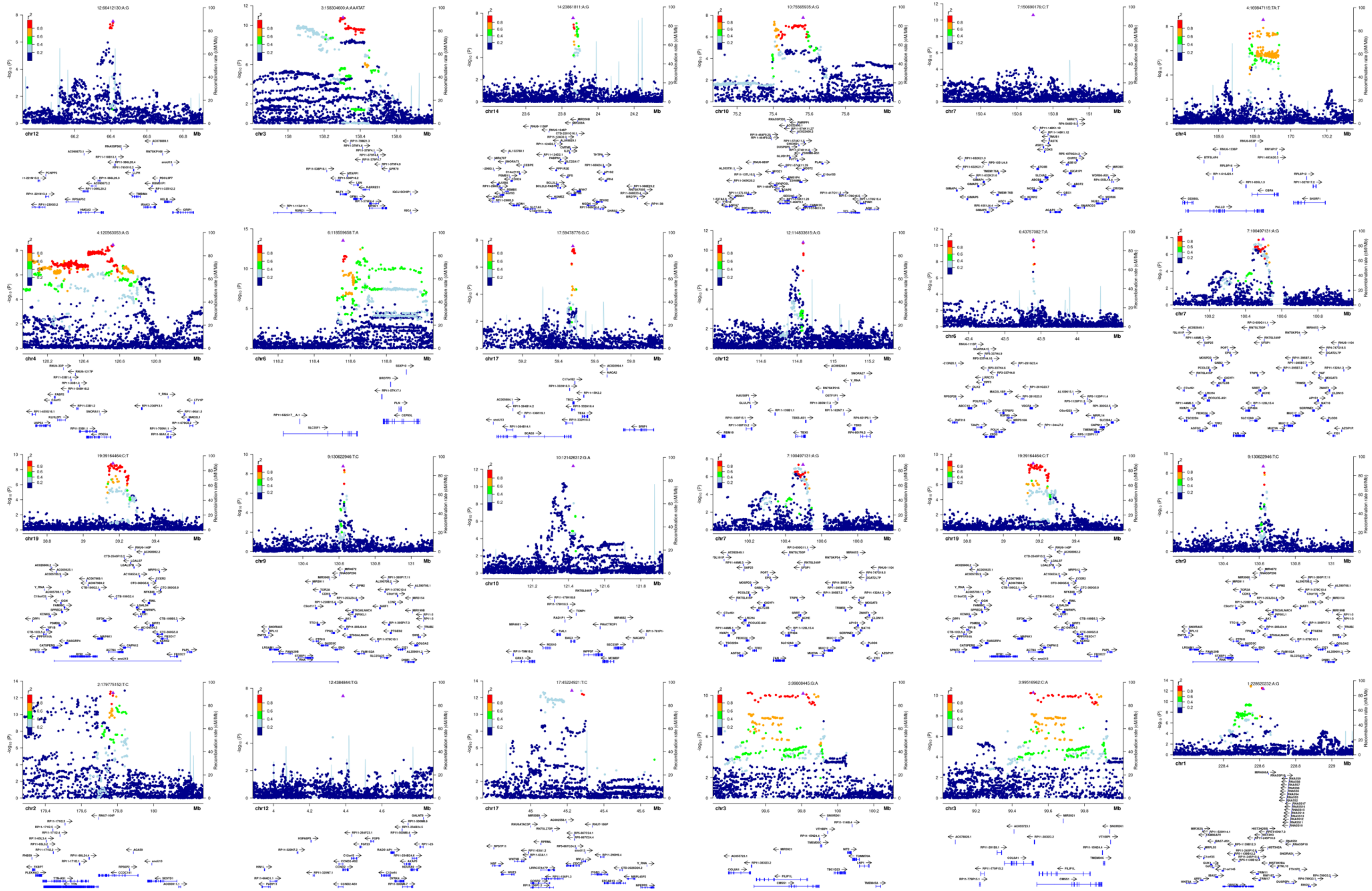

RVEDV

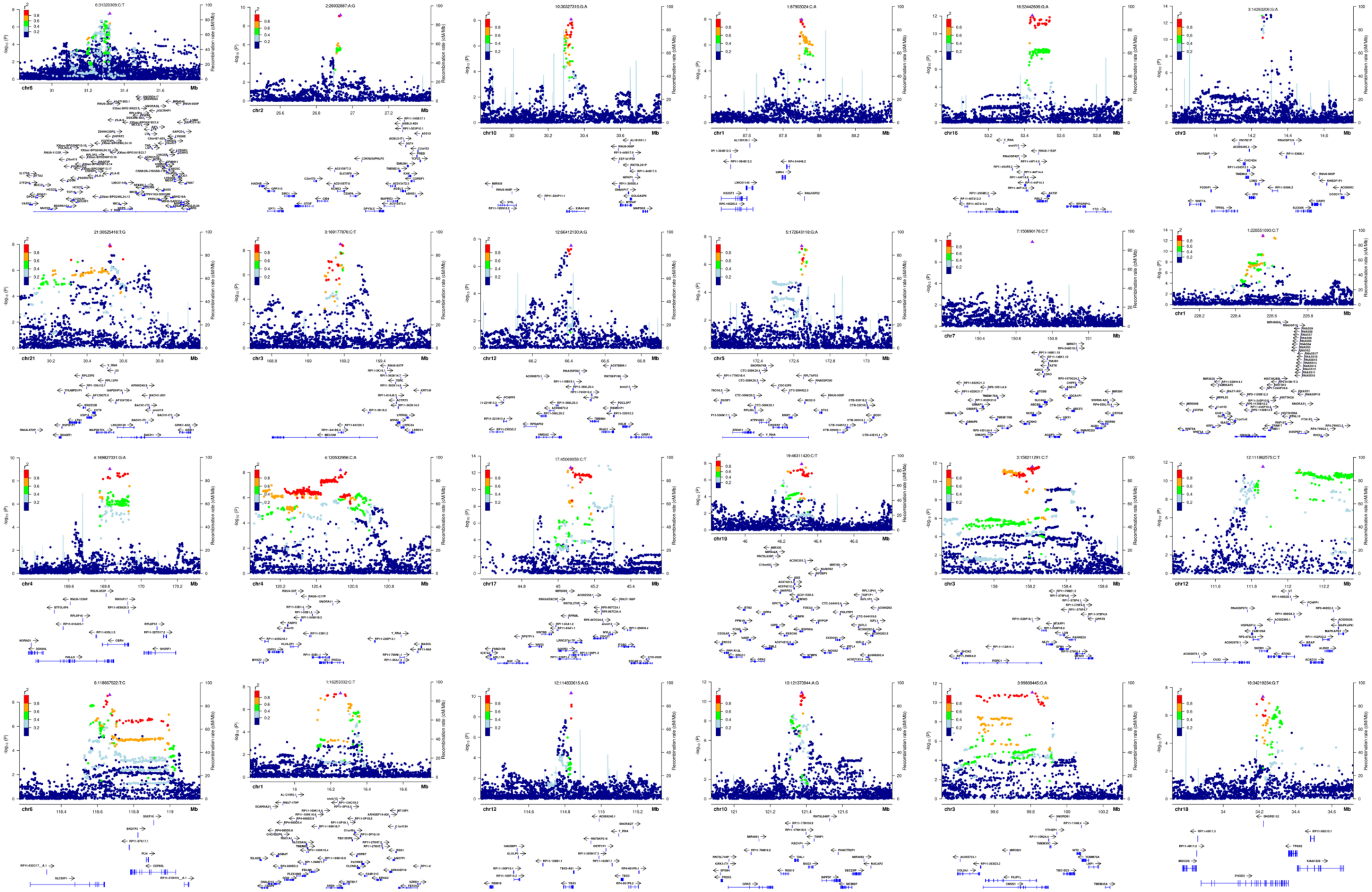

6:31320309:C:T
2:26932887:A:G
10:30327316:G:A
1:87903024:C:A
16:53442606:G:A
3:14263206:G:A
21:30525418:T:G
3:169177876:C:T
12:66412130:A:G
5:172643118:G:A
7:150690176:C:T
1:226551090:C:T
4:169827031:G:A
4:120532956:C:A
17:45069058:C:T
19:46311420:C:T
3:158211291:C:T
12:111862575:C:T
6:118667522:T:C
1:16253332:C:T
12:114833615:A:G
10:121373944:A:G
3:99808445:G:A
18:34219234:G:T

RVEDV

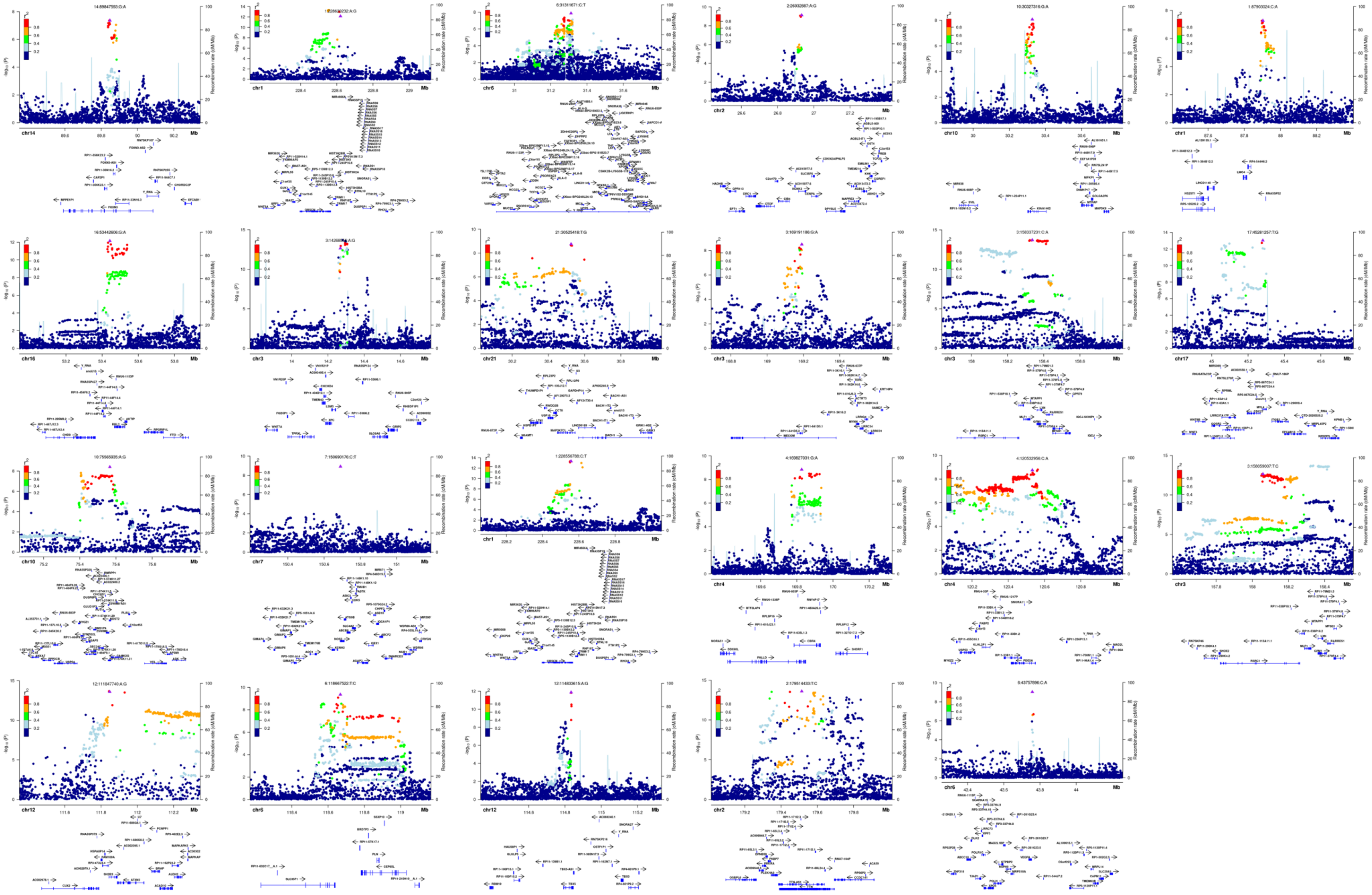

RVEDV_BSA

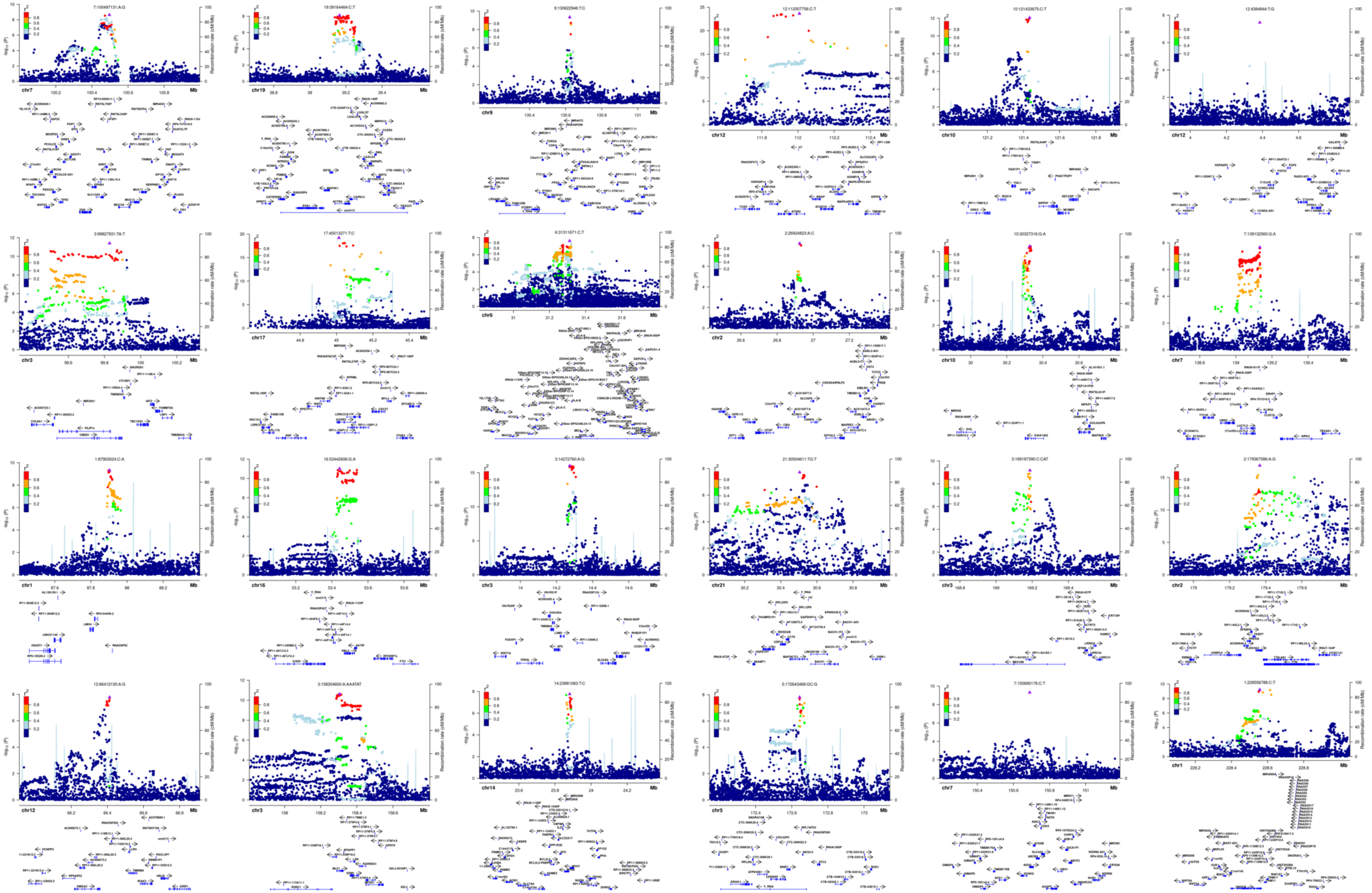
7:100497131:A:G
19:39164464:C:T
9:130622946:T:C
12:112007756:C:T
10:121433675:C:T
12:4384844:T:G
3:99827931:TA:T
17:45013271:T:C
6:31311671:C:T
2:26924823:A:C
10:30327316:G:A
7:139132560:G:A
1:87903024:C:A
16:53442606:G:A
3:14272760:A:G
21:30504611:TG:T
3:169187390:C:CAT
2:179367086:A:G
12:66412130:A:G
3:158304600:A:AAATAT
14:23881083:T:C
5:172643468:GC:G
7:150690176:C:T
1:228556788:C:T

RVEDV_BSA

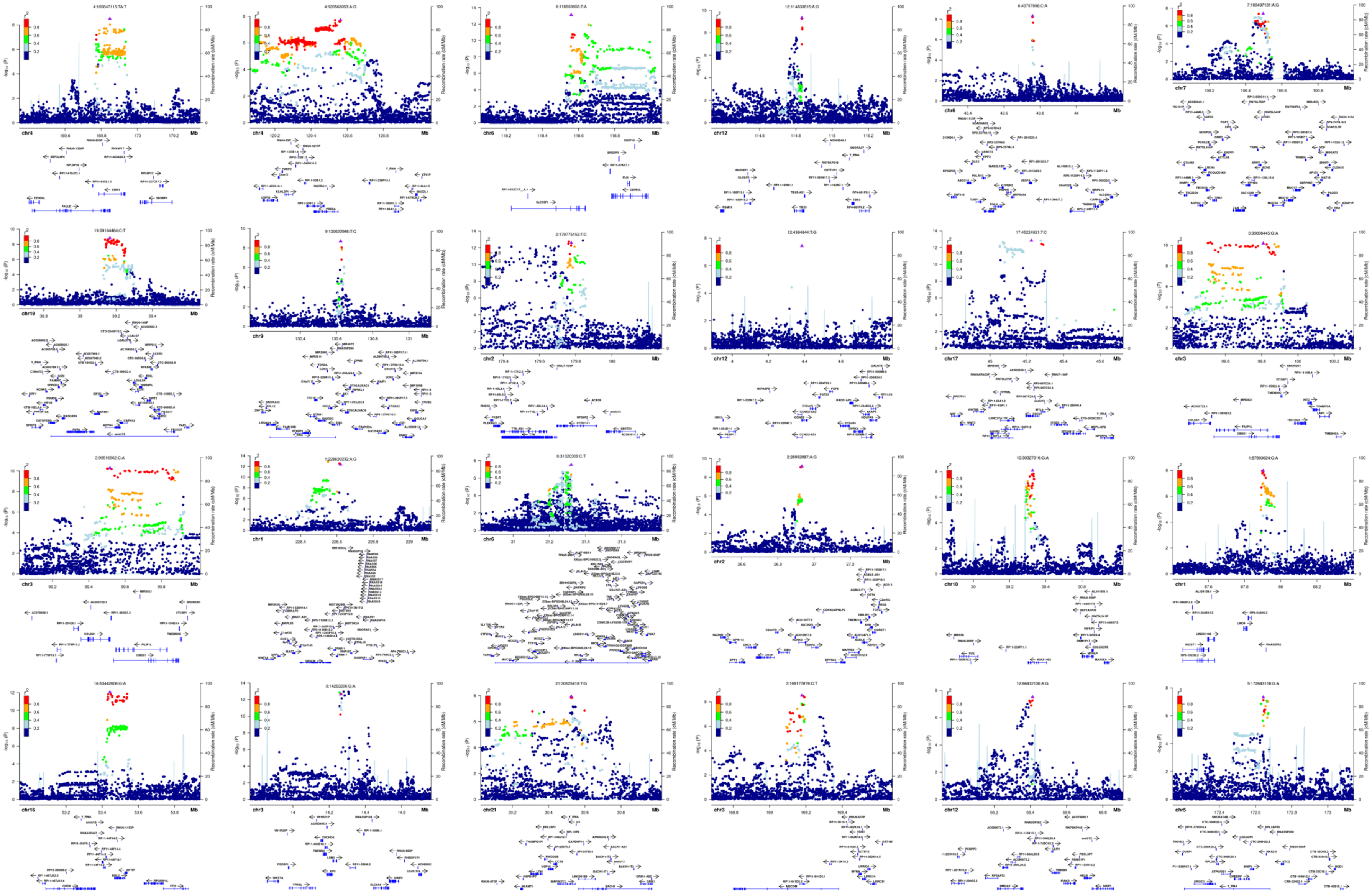

RVEDV_BSA

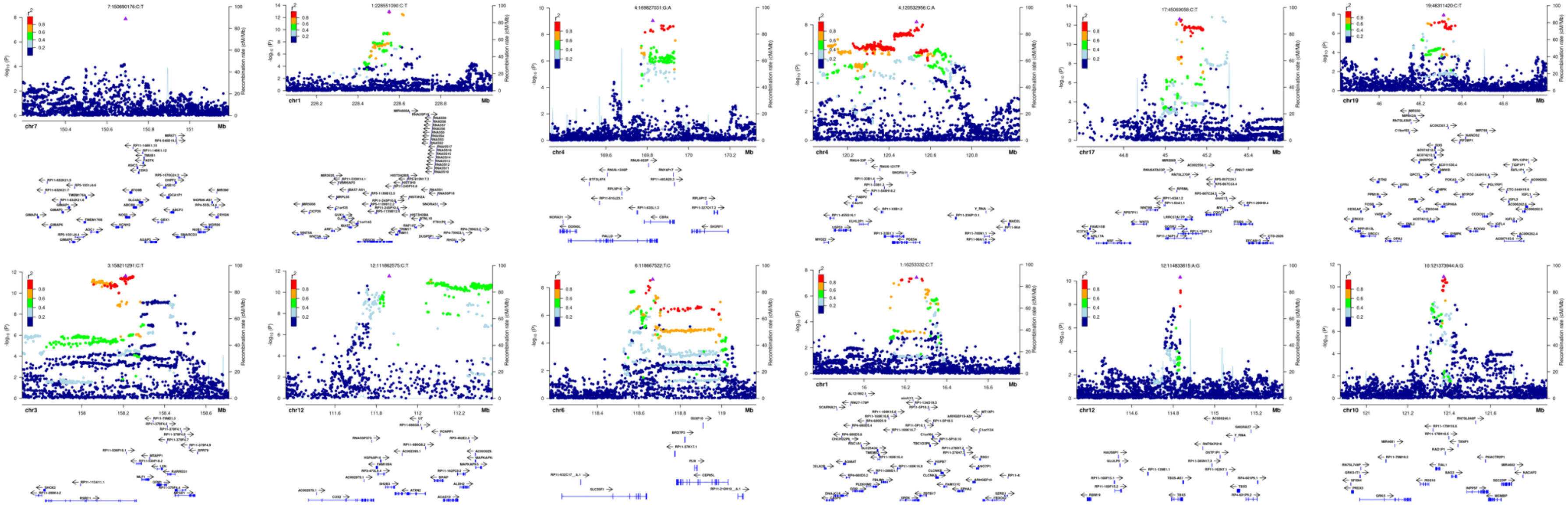

RVEF

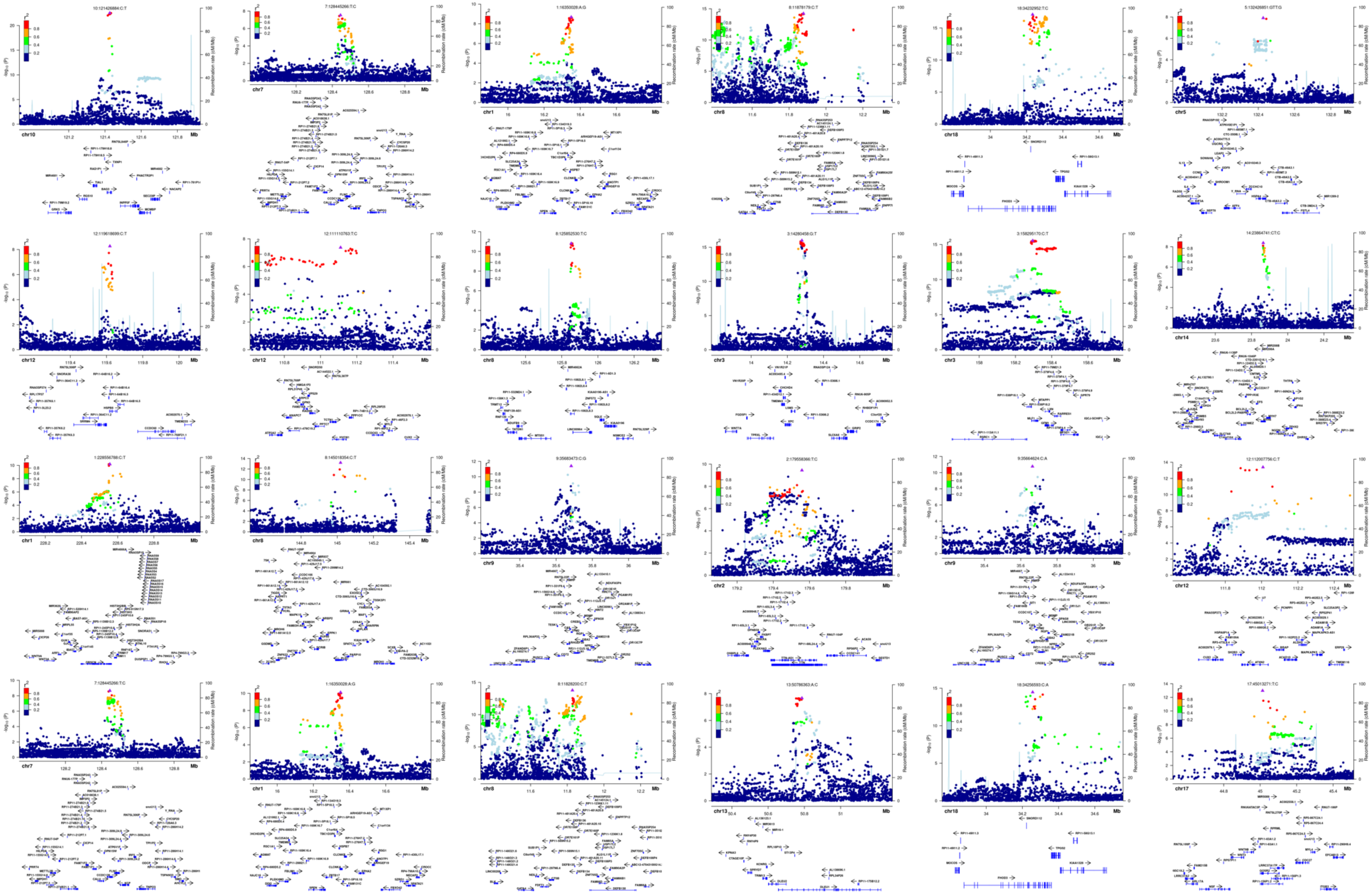
10:121426884:C:T
7:128445266:T:C
1:16350028:A:G
8:11878179:C:T
18:34232952:T:C
5:132426851:GTT:G
12:119618699:C:T
12:111110763:T:C
8:125852530:T:C
3:14280458:G:T
3:158295170:C:T
14:23864741:CT:C
1:228556788:C:T
8:145018354:C:T
9:35683473:C:G
2:179558366:T:C
9:35664624:C:A
12:112007756:C:T
7:128445266:T:C
1:16350028:A:G
8:11828200:C:T
13:50786363:A:C
18:34256593:C:A
17:45013271:T:C

RVEF

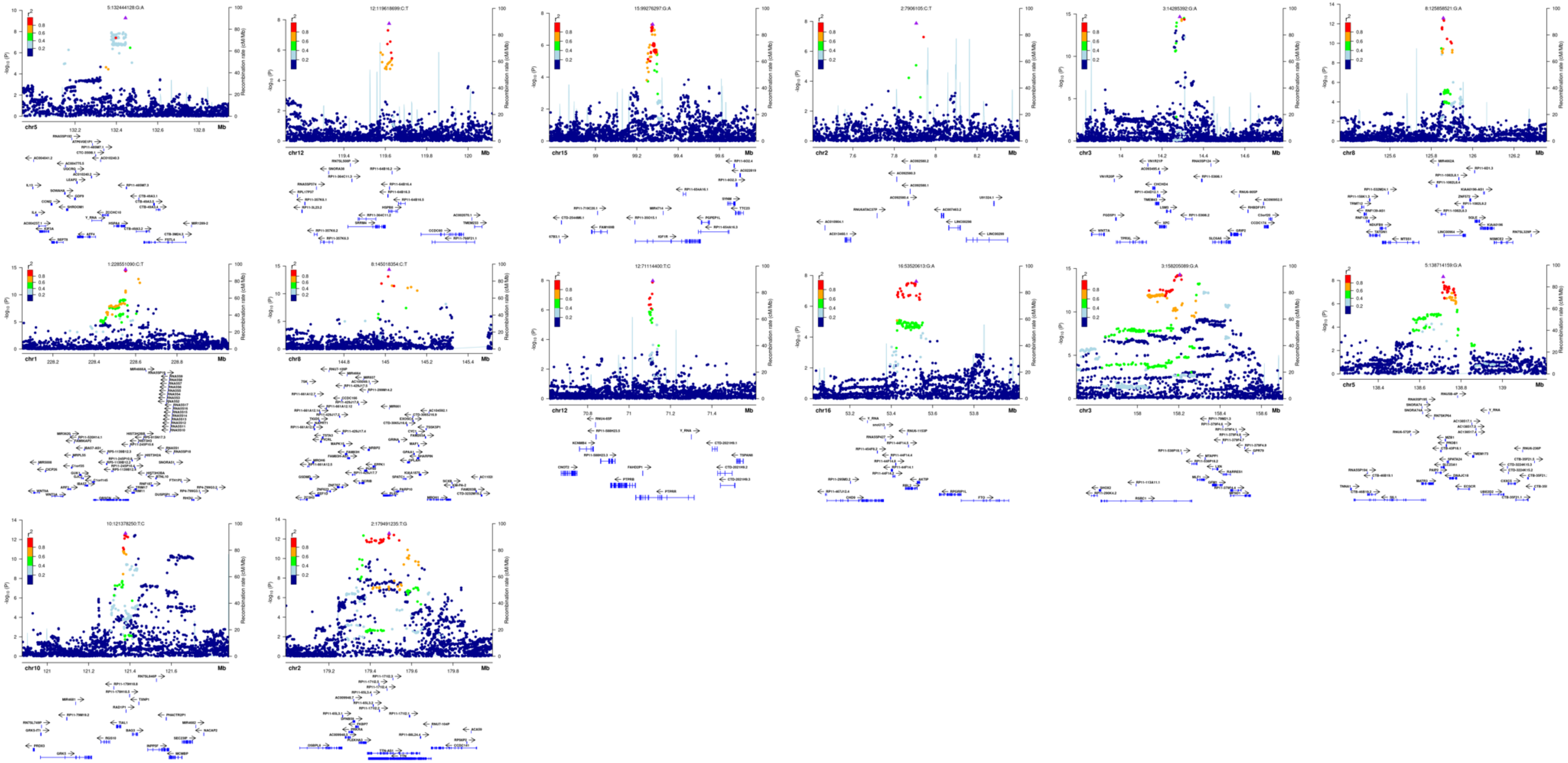

5:132444128:G:A
12:119618699:C:T
15:99276297:G:A
2:7906105:C:T
3:14285392:G:A
8:125858521:G:A
1:228551090:C:T
8:145018354:C:T
12:71114400:T:C
16:53520613:G:A
3:158205089:G:A
5:138714159:G:A
10:121378250:T:C
2:179491235:T:G

RVESV

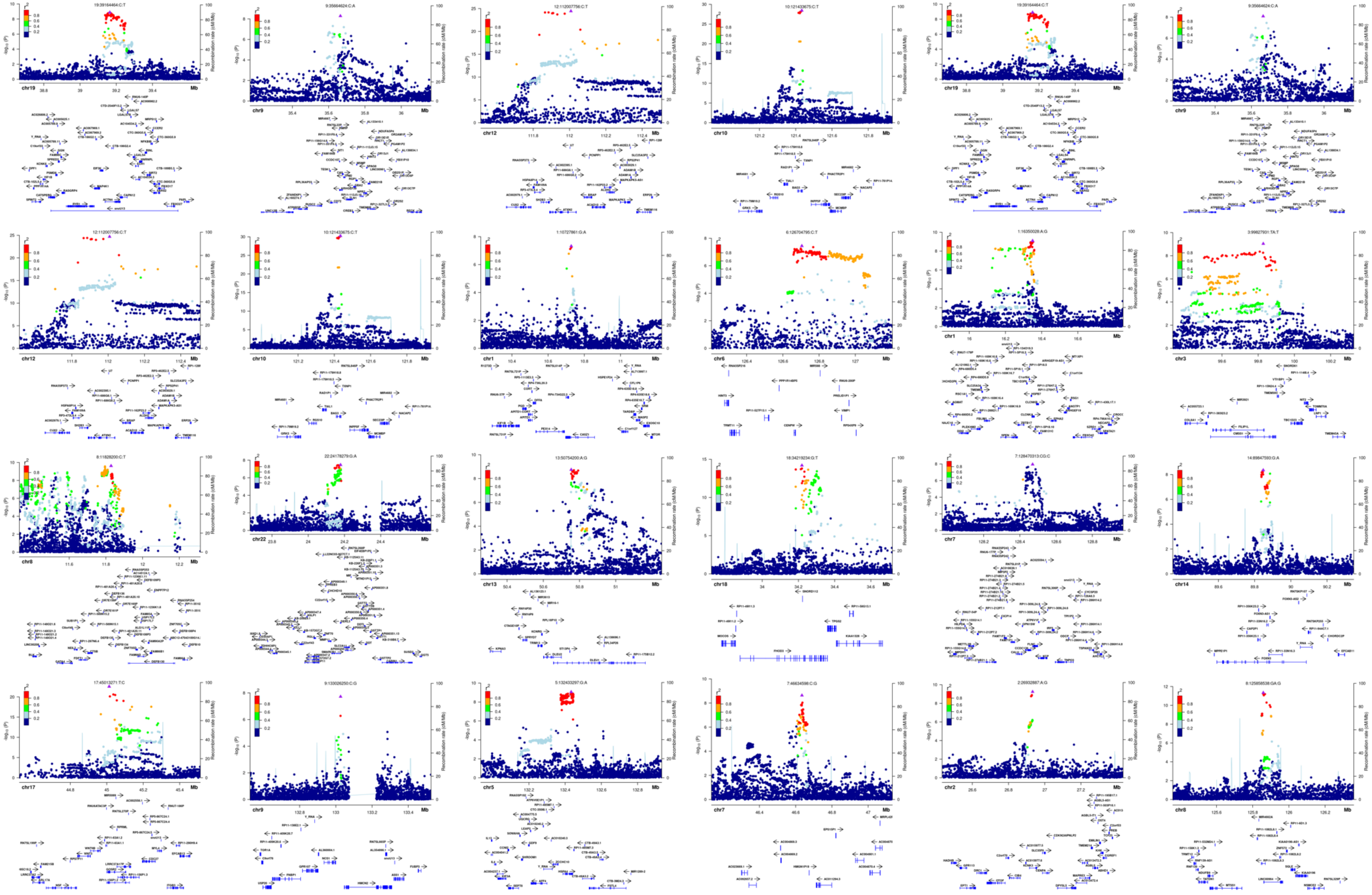

RVESV

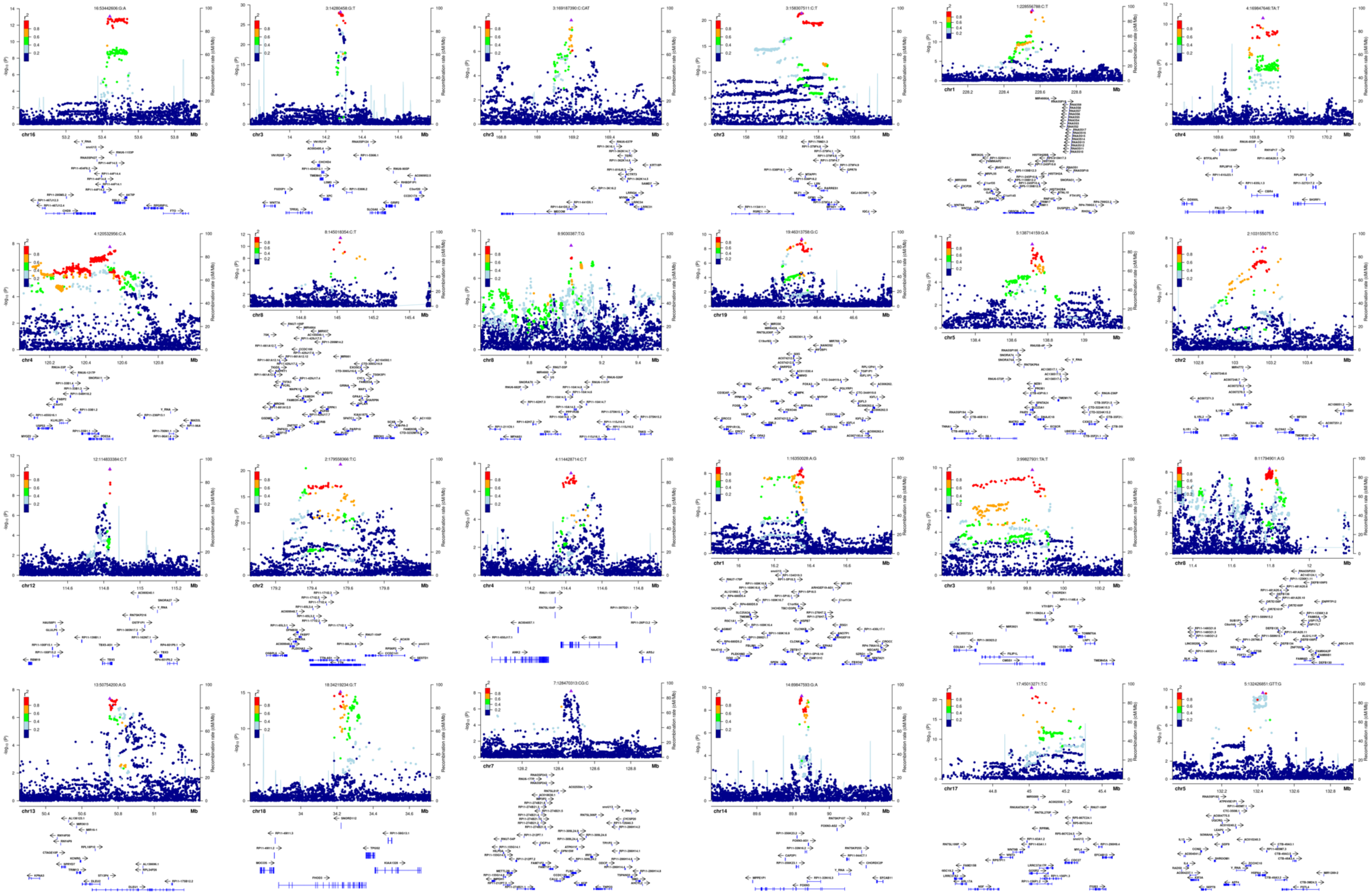
16:53442606:G:A
3:14280458:G:T
3:169187390:C:CAT
3:158307511:C:T
1:228556788:C:T
4:169847646:TA:T
4:120532956:C:A
8:145018354:C:T
8:9030387:T:G
19:46313758:G:C
5:138714159:G:A
2:103155075:T:C
12:114833384:C:T
2:179558366:T:C
4:114426714:C:T
1:16350028:A:G
3:99827931:TA:T
8:11794901:A:G
13:50754200:A:G
18:34219234:G:T
7:128470313:CG:C
14:89847593:G:A
17:45013271:T:C
5:132426851:GTT:G

RVESV

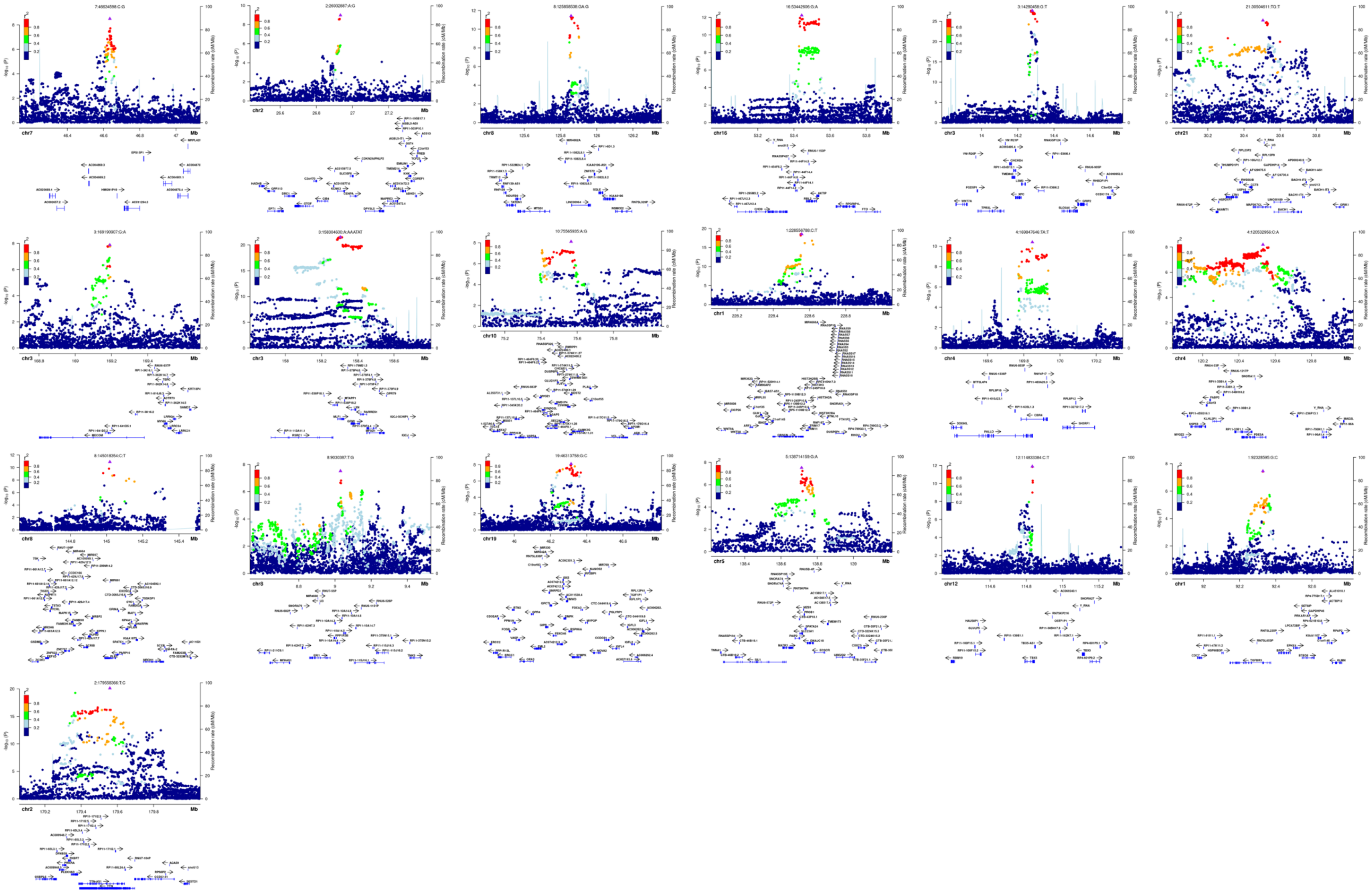
7:46634598:C:G
2:26932887:A:G
8:125858538:GA:G
16:53442606:G:A
3:14280458:G:T
21:30504611:TG:T
3:169190907:G:A
3:158304600:A:AAATAT
10:75565935:A:G
1:228556788:C:T
4:169847646:TA:T
4:120532956:C:A
8:145018354:C:T
8:9030387:T:G
19:46313758:G:C
5:138714159:G:A
12:114833384:C:T
1:92328595:G:C
2:179558366:T:C

RVESV_BSA

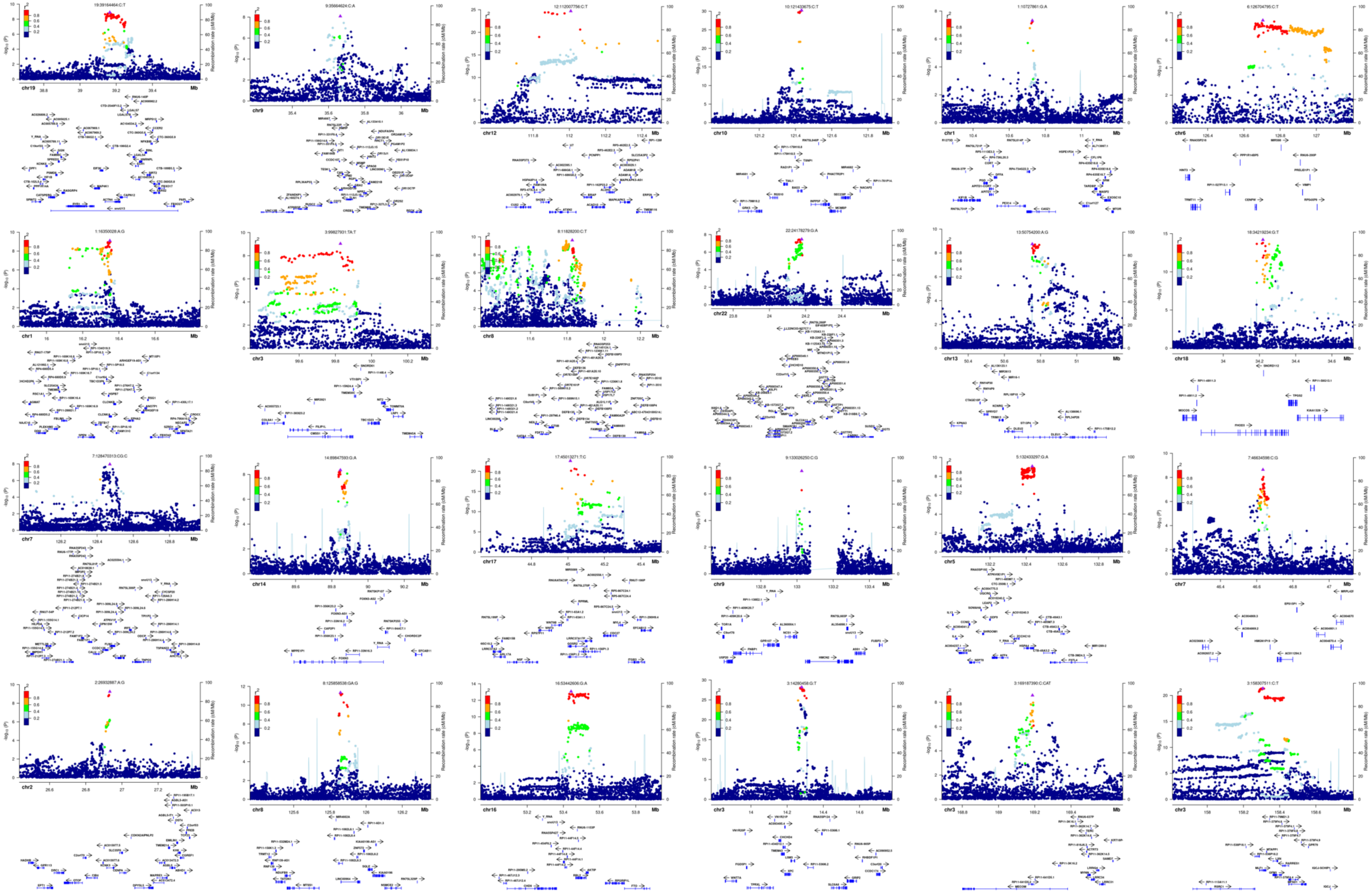
19:39164464:C:T
9:35664624:C:A
12:112007756:C:T
10:121433675:C:T
1:10727861:G:A
6:126704795:C:T
1:16350028:A:G
3:99827931:TA:T
8:11828200:C:T
22:24178279:G:A
13:50754200:A:G
18:34219234:G:T
7:128470313:CG:C
14:89847593:G:A
17:45013271:T:C
9:133026250:C:G
5:132433297:G:A
7:46634598:C:G
2:26932887:A:G
8:125858538:GA:G
16:53442606:G:A
3:14280458:G:T
3:169187390:C:CAT
3:158307511:C:T

RVESV_BSA

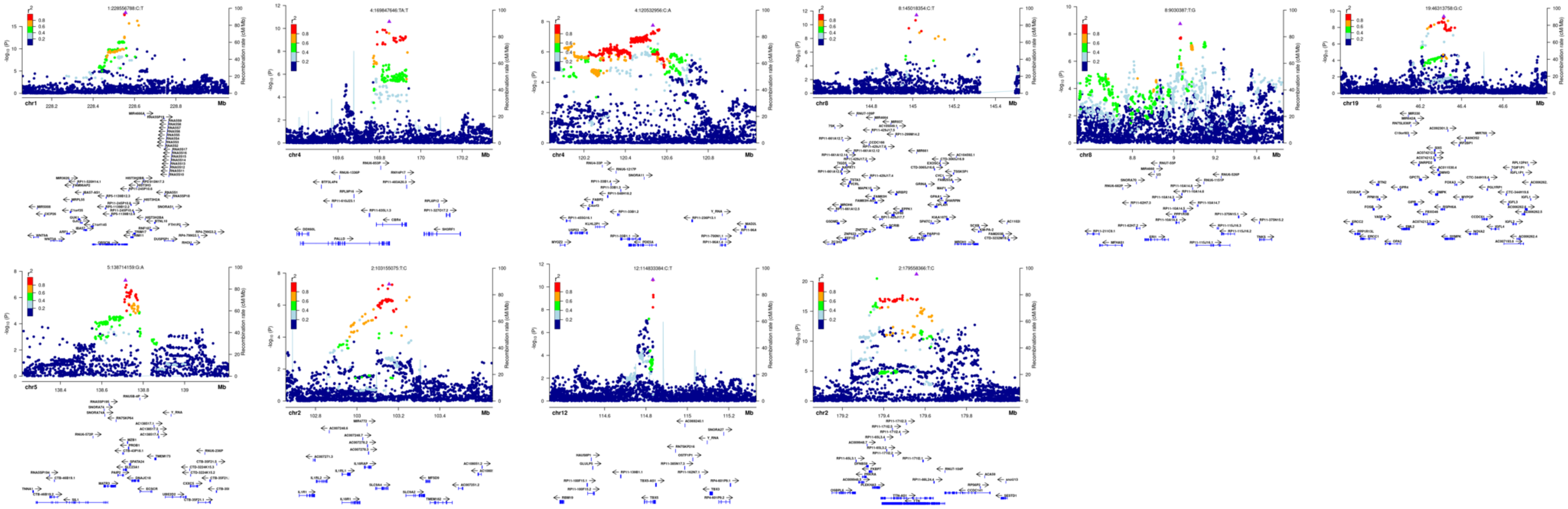

1:228556788:C:T
4:169847646:TA:T
4:120532956:C:A
8:145018354:C:T
8:9030387:T:G
19:46313758:G:C
5:138714159:G:A
2:103155075:T:C
12:114833384:C:T
2:179558366:T:C

RVSV

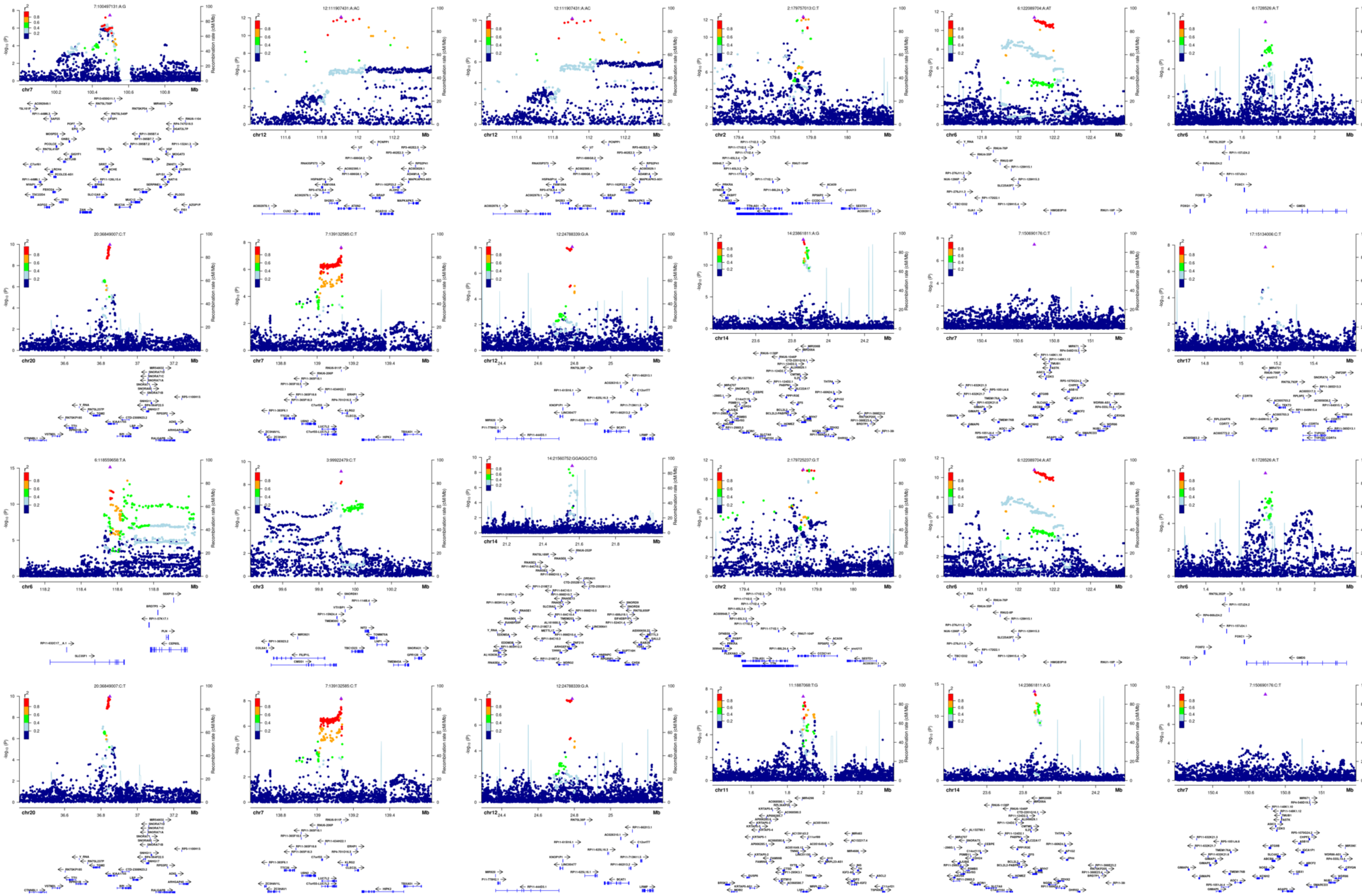

RVSV

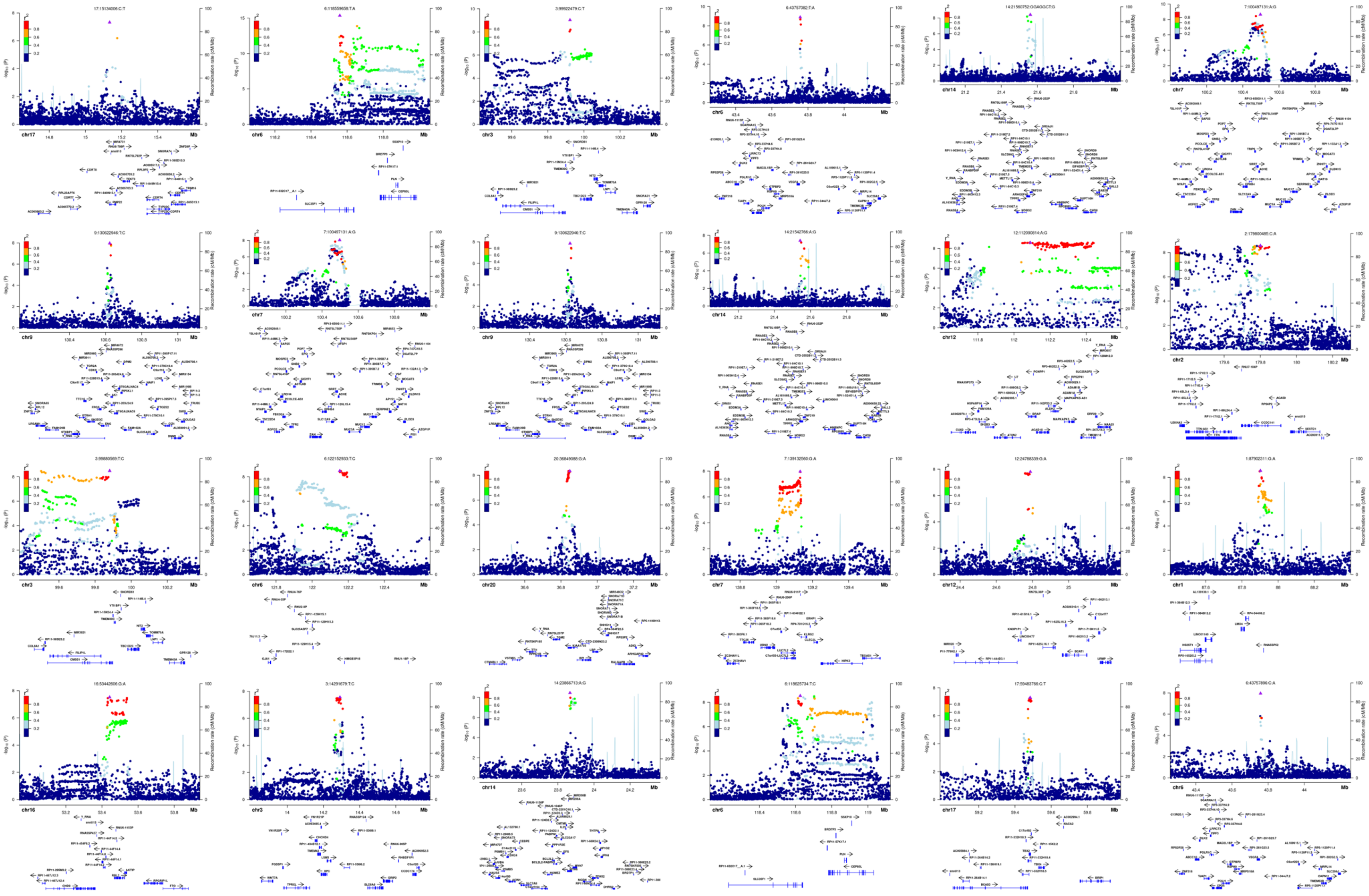

RVSV

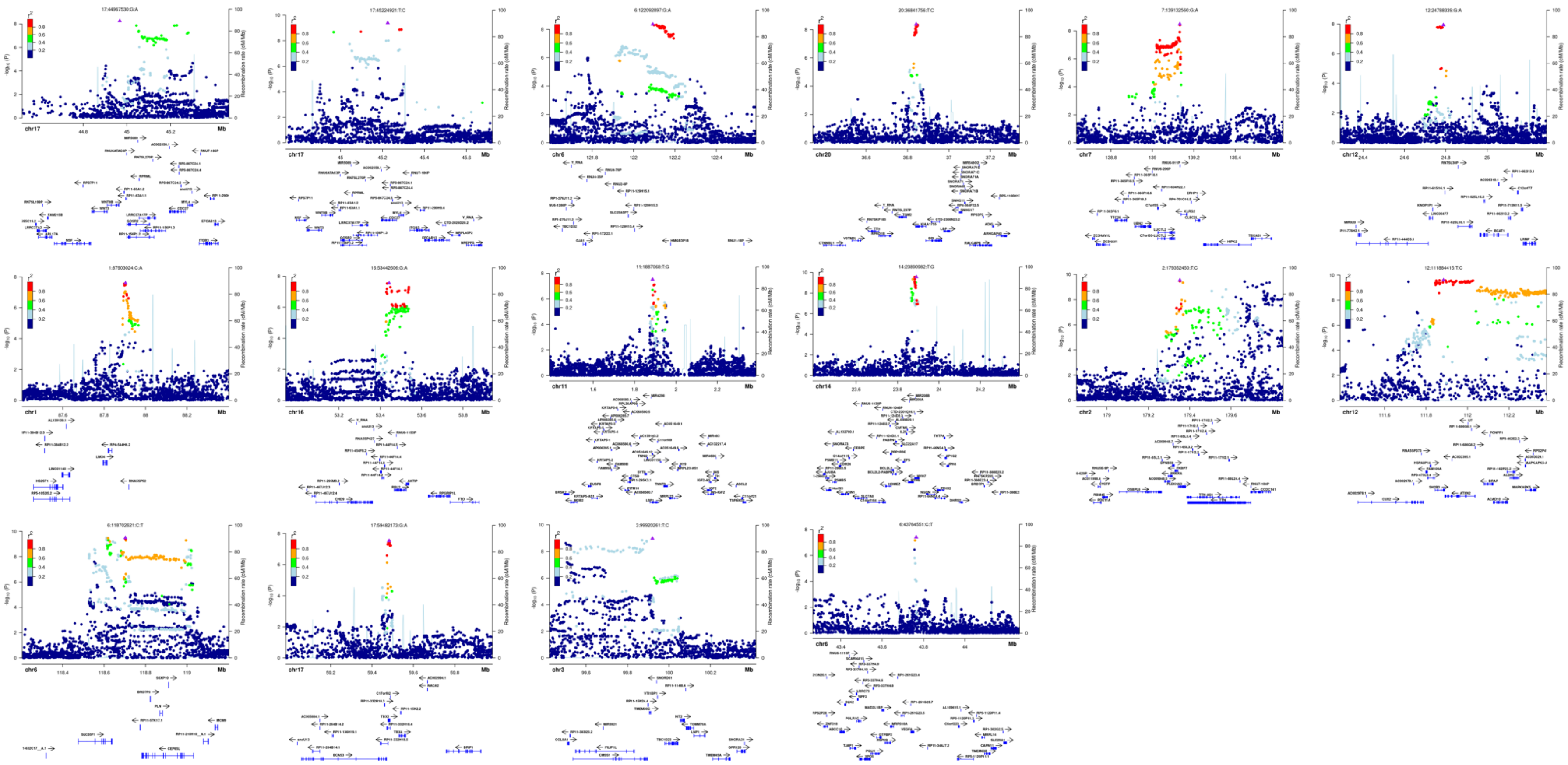
17:44967530:G:A
17:45224921:T:C
6:122092897:G:A
20:36841756:T:C
7:139132560:G:A
12:24788339:G:A
1:87903024:C:A
16:53442606:G:A
11:1887068:T:G
14:23890982:T:G
2:179352450:T:C
12:111884415:T:C
6:118702621:C:T
17:59482173:G:A
3:99920261:T:C
6:43764551:C:T

RVSV_BSA

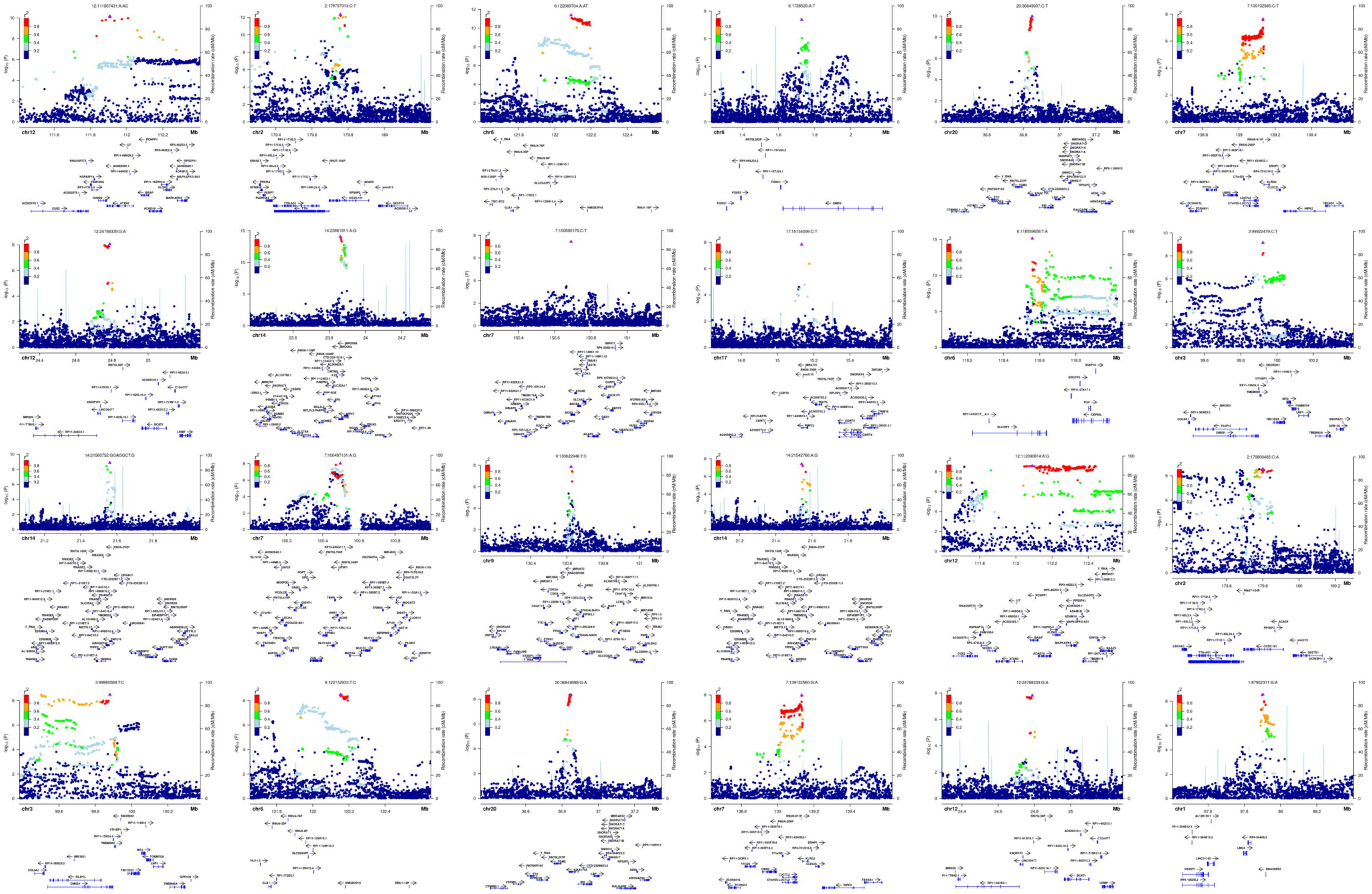

RVSV_BSA

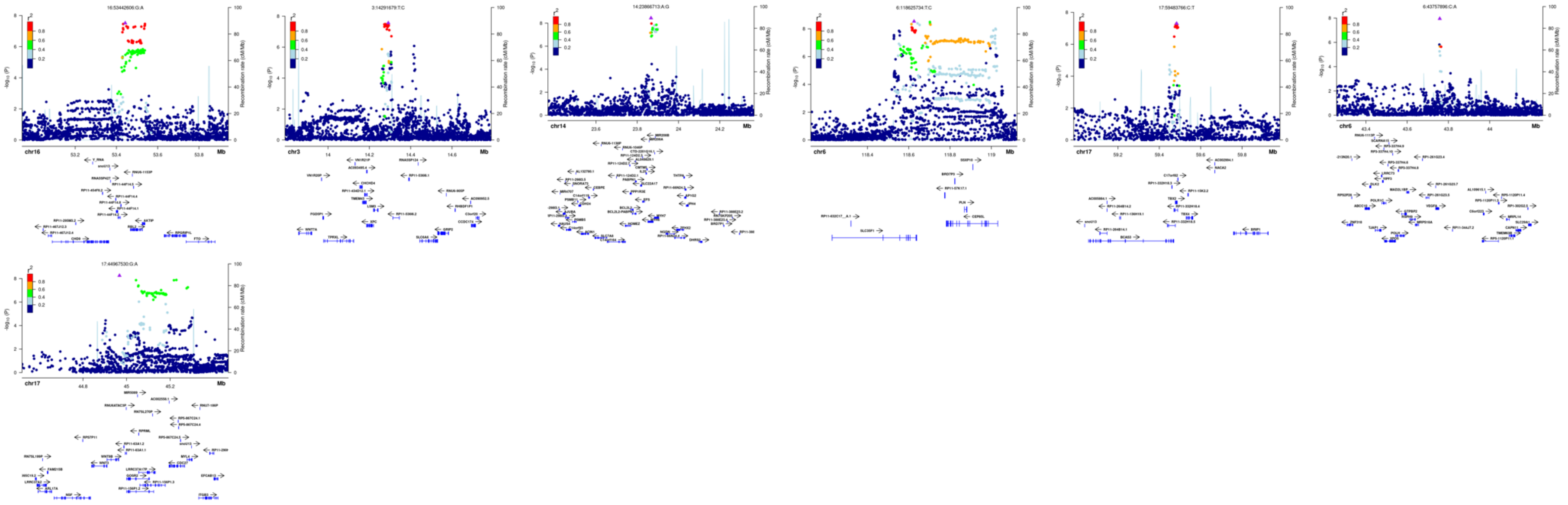

Supplementary Fig 3. LocusZoom plots of all left and right ventricular traits. Regional association plots of significant loci for left and right ventricular traits. P-values were calculated using a two-sided Wald test within the REGENIE linear regression framework. The additive genetic model was used to test for single-variant associations. The *y*-axis denotes the $\log_{10}$(*p*-value) and the *x*-axis denotes the genomic region. Linkage disequilibrium (r^2) for the lead variant (indicated by a purple triangle) was calculated based on the UK Biobank reference panel**.** The full summary statistics underlying these plots are publicly available via the GWAS Catalog (Accession codes: GCST90797570-GCST90797613). LVEDV, Left Ventricular End-Diastolic Volume; LVESV, Left Ventricular End-Systolic Volume; LVSV, Left Ventricular Stroke Volume; LVEF, Left Ventricular Ejection Fraction; LVGFI, Left Ventricular Global Function Index; LVMCF, Left Ventricular Myocardial Contraction Fraction; LVM, Left Ventricular Mass; LVMVR, Left Ventricular Mass-to-Volume Ratio; LVEDV BSA, Left Ventricular End-Diastolic Volume Indexed to Body Surface Area; LVESV BSA, Left Ventricular End-Systolic Volume Indexed to Body Surface Area; LVSV BSA, Left Ventricular Stroke Volume Indexed to Body Surface Area; LVM BSA, Left Ventricular Mass Indexed to Body Surface Area; RVEDV, Right Ventricular End-Diastolic Volume; RVESV, Right Ventricular End-Systolic Volume; RVSV, Right Ventricular Stroke Volume; RVEF, Right Ventricular Ejection Fraction; RV LV ratio, Right Ventricular to Left Ventricular Ratio; RVEDV BSA, Right Ventricular End-Diastolic Volume Indexed to Body Surface Area; RVESV BSA, Right Ventricular End-Systolic Volume Indexed to Body Surface Area; RVSV BSA, Right Ventricular Stroke Volume Indexed to Body Surface Area.

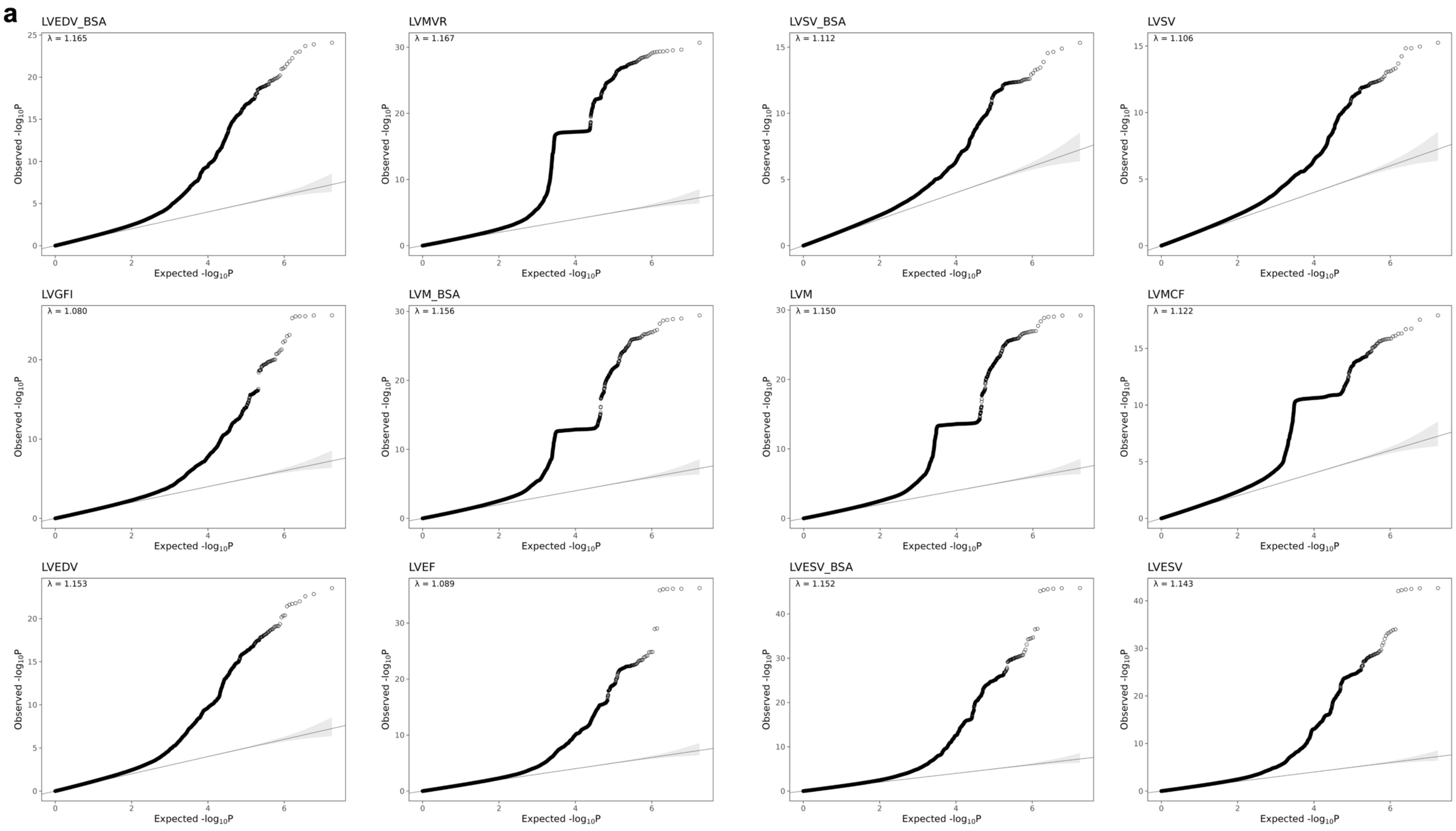
a
LVEDV_BSA
λ = 1.165
LVMVR
λ = 1.167
LVSV_BSA
λ = 1.112
LVSV
λ = 1.106
LVGFI
λ = 1.080
LVM_BSA
λ = 1.156
LVM
λ = 1.150
LVMCF
λ = 1.122
LVEDV
λ = 1.153
LVEF
λ = 1.089
LVESV_BSA
λ = 1.152
LVESV
λ = 1.143
Observed $-\log_{10}P$
Expected $-\log_{10}P$

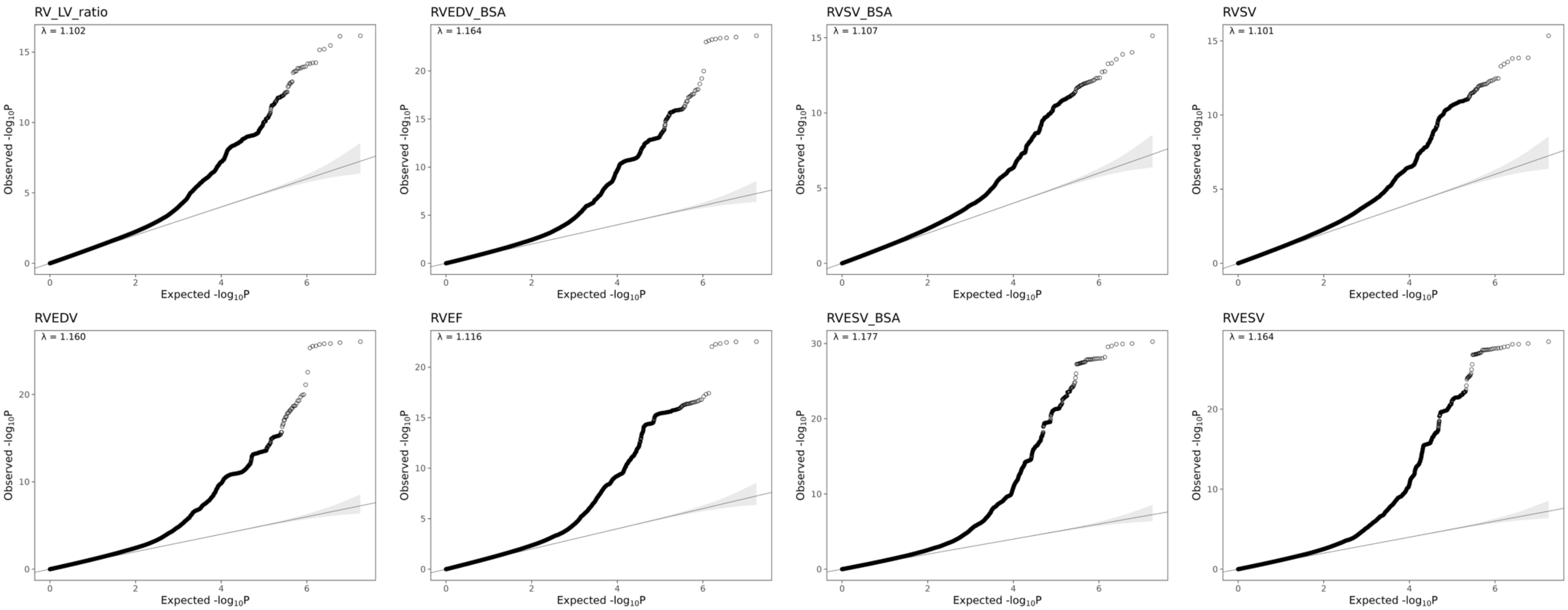
RV_LV_ratio
λ = 1.102
RVEDV_BSA
λ = 1.164
RVSV_BSA
λ = 1.107
RVSV
λ = 1.101
RVEDV
λ = 1.160
RVEF
λ = 1.116
RVESV_BSA
λ = 1.177
RVESV
λ = 1.164
Observed -log10P
Expected -log10P

b

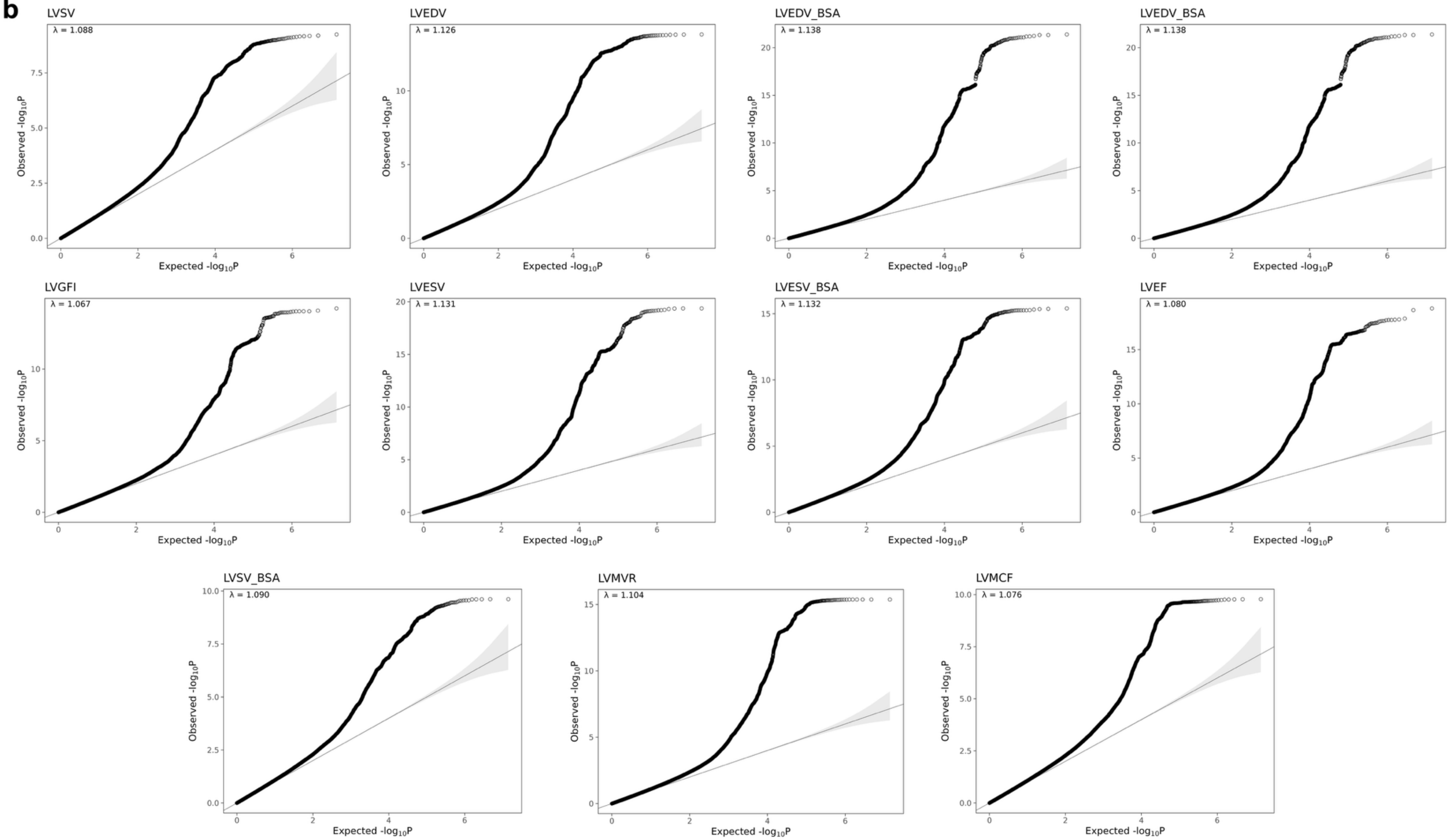
LVSV
λ = 1.088
LVEDV
λ = 1.126
LVEDV_BSA
λ = 1.138
LVEDV_BSA
λ = 1.138
LVGFI
λ = 1.067
LVESV
λ = 1.131
LVESV_BSA
λ = 1.132
LVEF
λ = 1.080
LVSV_BSA
λ = 1.090
LVMVR
λ = 1.104
LVMCF
λ = 1.076
Observed $-\log_{10}P$
Expected $-\log_{10}P$

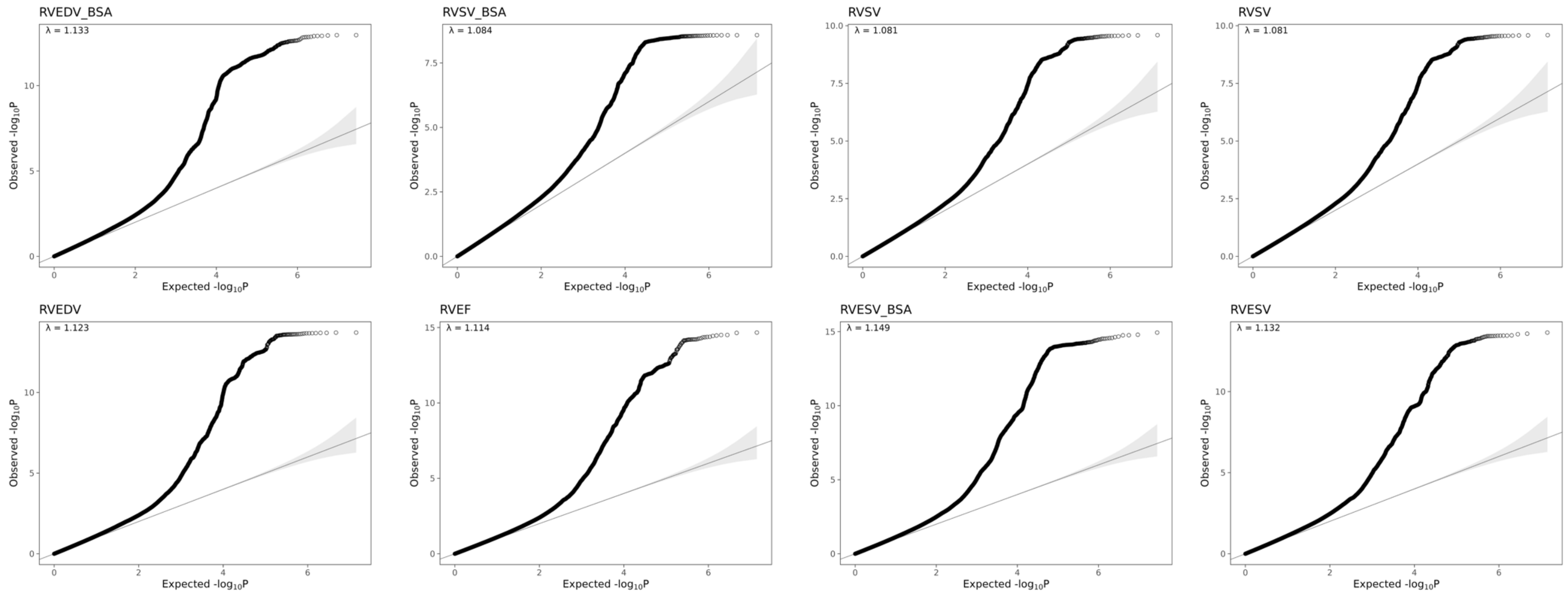

Supplementary Fig 4. QQ plots of all left and right ventricular traits. Single-trait quantile-quantile (QQ) plots (a) and multi-trait genome-wide association analysis QQ plots (b). P-values were derived from two-sided Wald tests using a linear regression model within the REGENIE framework. λ is the genomic inflation factor. The *y*-axis denotes the observed $\log_{10}$(*p*-value) and the *x*-axis denotes the expected $\log_{10}$(*p*-value). The full summary statistics underlying these plots are publicly available via the GWAS Catalog (Accession codes: GCST90797570-GCST90797613). LVEDV, Left Ventricular End-Diastolic Volume; LVESV, Left Ventricular End-Systolic Volume; LVSV, Left Ventricular Stroke Volume; LVEF, Left Ventricular Ejection Fraction; LVGFI, Left Ventricular Global Function Index; LVMCF, Left Ventricular Myocardial Contraction Fraction; LVM, Left Ventricular Mass; LVMVR, Left Ventricular Mass-to-Volume Ratio; LVEDV BSA, Left Ventricular End-Diastolic Volume Indexed to Body Surface Area; LVESV BSA, Left Ventricular End-Systolic Volume Indexed to Body Surface Area; LVSV BSA, Left Ventricular Stroke Volume Indexed to Body Surface Area; LVM BSA, Left Ventricular Mass Indexed to Body Surface Area; RVEDV, Right Ventricular End-Diastolic Volume; RVESV, Right Ventricular End-Systolic Volume; RVSV, Right Ventricular Stroke Volume; RVEF, Right Ventricular Ejection Fraction; RV LV ratio, Right Ventricular to Left Ventricular Ratio; RVEDV BSA, Right Ventricular End-Diastolic Volume Indexed to Body Surface Area; RVESV BSA, Right

Ventricular End-Systolic Volume Indexed to Body Surface Area; RVSV BSA, Right Ventricular Stroke Volume Indexed to Body Surface Area.

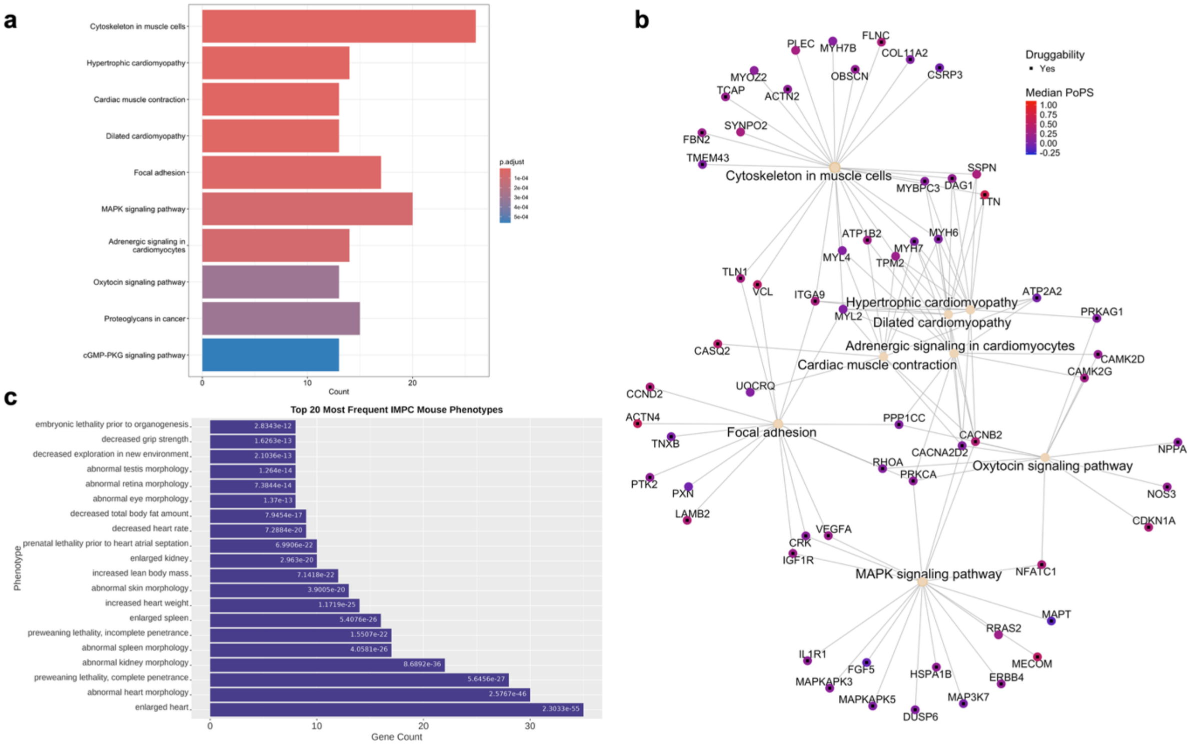

Supplementary Fig 5. Gene enrichment analysis. (a) shows the most significantly enriched KEGG (Kyoto Encyclopedia of Genes and Genomes) pathways for the 488 prioritised genes. Statistical significance was determined using a two-sided Fisher's Exact Test with p-values adjusted for multiple comparisons using the Benjamini-Hochberg False Discovery Rate (FDR) method. (b) visualises the gene network interaction between the top eight most significantly enriched KEGG pathways. In panel b, genes that have a "druggable" annotation in the drug-gene interaction database are denoted by a black square. The median PoPS (polygenic priority score) is identified per each gene node, using a red to blue colour gradient identifying highest (red) to lowest (blue) PoP scores. (c) shows a bar chart of the top 20 most significantly enriched knockout mouse model phenotypes and the counts of all their prioritised genes per phenotype. Statistical significance was assessed using a two-sided Fisher's Exact Test, with p-values adjusted for multiple comparisons using the FDR method. Source data are provided as a Source Data file.

a

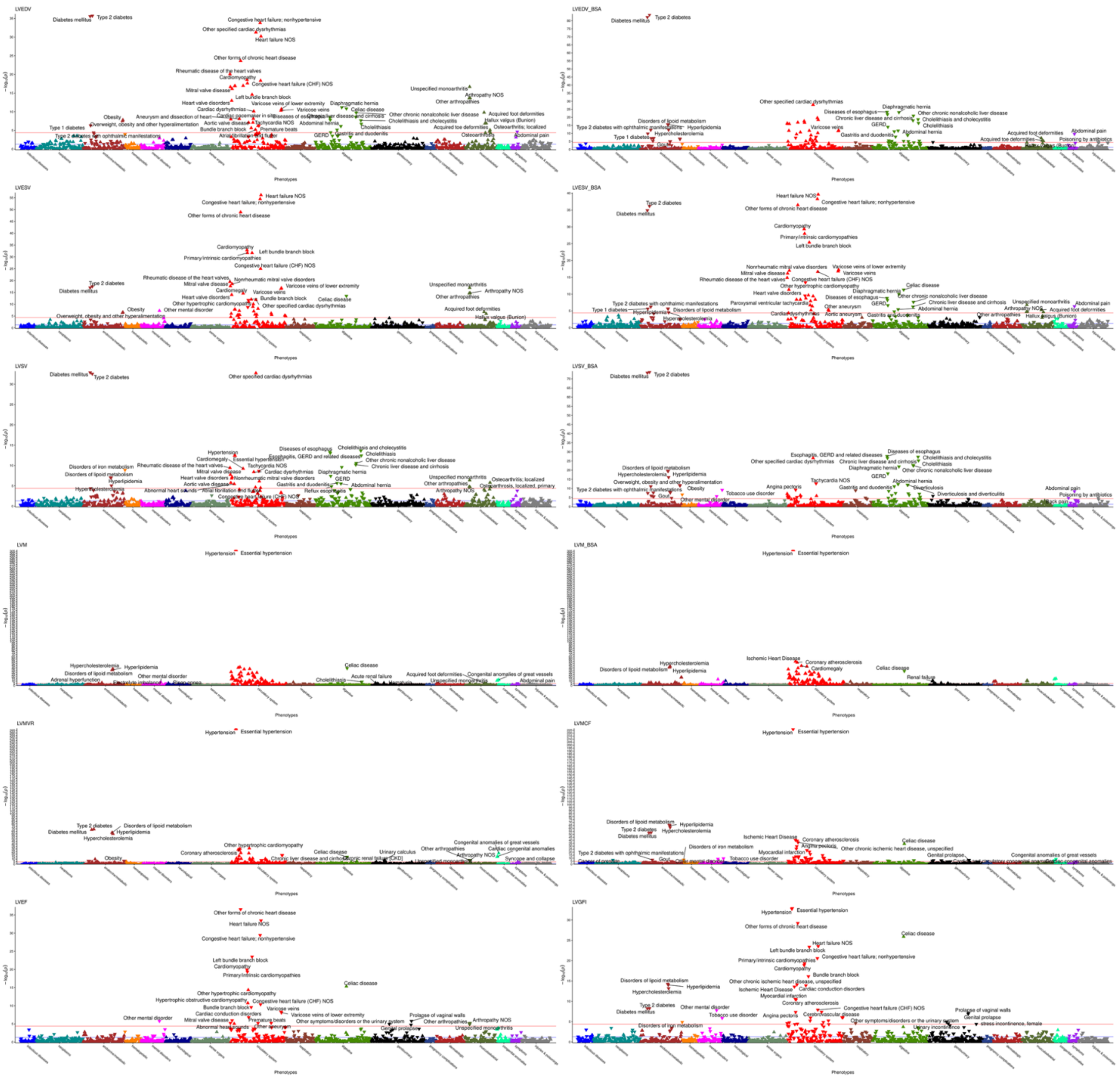
LVEDV
LVEDV_BSA
LVESV
LVESV_BSA
LVSV
LVSV_BSA
LVM
LVM_BSA
LVMVR
LVMCF
LVEF
LVGFI
Phenotypes

b

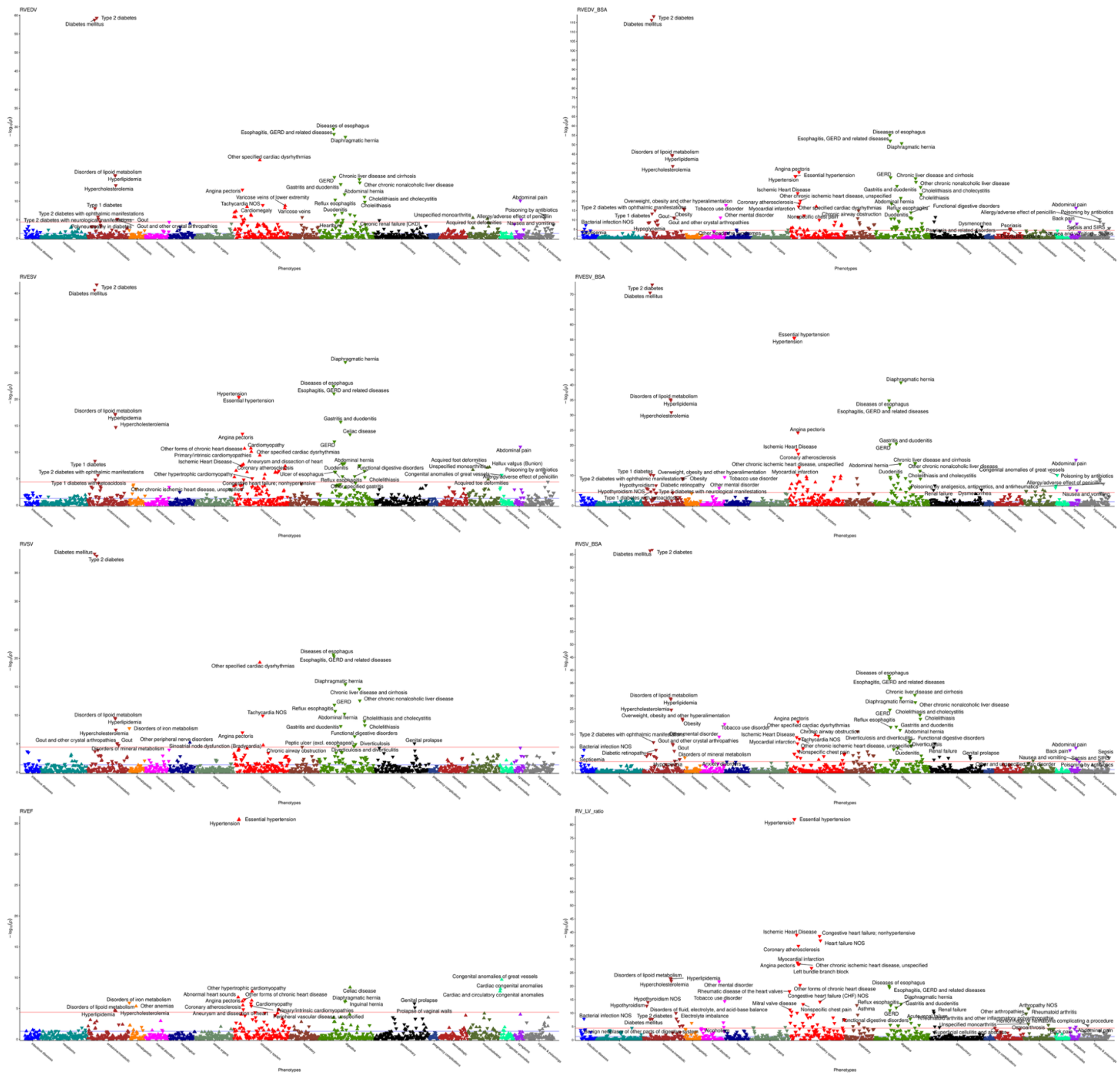

Supplementary Fig 6. Phenome-wide association analysis of left and right ventricular polygenic risk scores. In total, 1,855 phenotypes were analysed for associations with left ventricular (a) and right ventricular (b) polygenic risk scores adjusted for age, sex, and ten principal components. The associations were tested using two-sided regression models across phenotypes. Multiple testing was accounted for using a Bonferroni-adjusted phenome-wide significance threshold. The red line indicates the adjusted p-value significance threshold ($P<2.69x10^{-5}$) and the blue line indicates the nominal p-value threshold ($P<0.05$). The *y*-axis denotes the log_{10}(*p*-value) and the x-axis denotes the disease category. The triangles indicate positive and negative correlations (depicted by upright or inverted pointing directions respectively) and are colour-coded by disease category. Source data are provided as a Source Data file.
