# Supplementary material for: Genome-wide analysis of cardiac ventricular phenotypes reveals novel loci and therapeutic targets for heart failure
Source: Nat Commun. 2026 Feb 27;17:3293. doi: 10.1038/s41467-026-69982-0 (PMC13066029; doi:10.1038/s41467-026-69982-0)
Supplement: Supplementary file 2 — Description of Additional Supplementary Files [file 41467_2026_69982_MOESM2_ESM.pdf]

Description of Additional Supplementary Files

Supplementary Data 1: Cohort characteristics of the UK Biobank and Phenotype Definitions

Supplementary Data 2: Novel cardiovascular magnetic resonance imaging lead variants for all left and right ventricular phenotypes

Supplementary Data 3: Lead variants for all left and right ventricular phenotypes.

Supplementary Data 4: Lookup of all left and right ventricular GWAS lead variants in previously published GWAS of CMR-derived left and right ventricular phenotypes

Supplementary Data 5: Lookup of the left ventricular GWAS lead variants and their proxies in 99% credible sets in Phenoscanner v2

Supplementary Data 6: Lookup of the right ventricular GWAS lead variants and their proxies in 99% credible sets in Phenoscanner v2

Supplementary Data 7: GWAS catalog variant annotation of loci

Supplementary Data 8: Summary of left and right ventricular variant-level annotations in 99% credible sets

Supplementary Data 9: DEPICT gene set enrichment analysis results

Supplementary Data 10: Colocalization analysis with Heart Failure GWAS

Supplementary Data 11: Polygenic Priority Scores for all left and right ventricular associated genes

Supplementary Data 12: Summary of left and right ventricular gene-level annotations

Supplementary Data 13: Significant gene sets and pathways in g:Profiler

Supplementary Data 14: Enrichment analysis of IMPC mouse model phenotypes

Supplementary Data 15: Ranked genes drug indications and toxicity

Supplementary Data 16: Enrichment analysis of cardiovascular drug mechanisms of action

Supplementary Data 17: Gene burden testing associations

Supplementary Data 18: Phenome-wide Associations with all left and right ventricular phenotypes polygenic risk scores
