# Supplementary material for: Genome-wide analysis of cardiac ventricular phenotypes reveals novel loci and therapeutic targets for heart failure
Source: Nat Commun. 2026 Feb 27;17:3293. doi: 10.1038/s41467-026-69982-0 (PMC13066029; doi:10.1038/s41467-026-69982-0)
Supplement: Supplementary file 21 — Reporting Summary [file 41467_2026_69982_MOESM21_ESM.pdf]

nature portfolio

Corresponding author(s): Dr Nay Aung

Last updated by author(s): Dec 10, 2024

Reporting Summary

Nature Portfolio wishes to improve the reproducibility of the work that we publish. This form provides structure for consistency and transparency in reporting. For further information on Nature Portfolio policies, see our Editorial Policies and the Editorial Policy Checklist.

Statistics

For all statistical analyses, confirm that the following items are present in the figure legend, table legend, main text, or Methods section.

n/a	Confirmed	
☐	☒	The exact sample size (*n*) for each experimental group/condition, given as a discrete number and unit of measurement
☐	☒	A statement on whether measurements were taken from distinct samples or whether the same sample was measured repeatedly
☐	☒	The statistical test(s) used AND whether they are one- or two-sided<br>*Only common tests should be described solely by name; describe more complex techniques in the Methods section.*
☐	☒	A description of all covariates tested
☐	☒	A description of any assumptions or corrections, such as tests of normality and adjustment for multiple comparisons
☐	☒	A full description of the statistical parameters including central tendency (e.g. means) or other basic estimates (e.g. regression coefficient) AND variation (e.g. standard deviation) or associated estimates of uncertainty (e.g. confidence intervals)
☐	☒	For null hypothesis testing, the test statistic (e.g. *F*, *t*, *r*) with confidence intervals, effect sizes, degrees of freedom and *P* value noted<br>*Give P values as exact values whenever suitable.*
☒	☐	For Bayesian analysis, information on the choice of priors and Markov chain Monte Carlo settings
☒	☐	For hierarchical and complex designs, identification of the appropriate level for tests and full reporting of outcomes
☒	☐	Estimates of effect sizes (e.g. Cohen's *d*, Pearson's *r*), indicating how they were calculated

Our web collection on statistics for biologists contains articles on many of the points above.

Software and code

Policy information about availability of computer code

Data collection	*Provide a description of all commercial, open source and custom code used to collect the data in this study, specifying the version used OR state that no software was used.*
Data analysis	Data was analysed with several open source software and programming packages (R [v4.1.1], Python [v3.9.19], PLINK [v1.9], REGENIE [v3], GCTA [v1.94.1], LDSC [v1.0.1], MTAG [v1.0.8], DEPICT, MAGMA [v1.10], PoPs [v0.2], DrugnomeAI [v1.0.0], Exomiser [v14.0.0], g:Profiler R package (v2_0.2.2), Drugenrichr [https://maayanlab.cloud/DrugEnrichr/], coloc R package [v5.2.3], Variant Effect Predictor [108 release], poolr R package[v1.2-0], and PheWAS R package [v1.0]). We used publicly available software for all the analyses, and all software used is listed and described with sufficient details in the Methods section of our manuscript. We also include code used to perform our GWAS in our Code Availability statement (https://doi.org/10.5281/zenodo.18431669). The algorithms for CMR image analysis are available in https://github.com/baiwenjia/ukbb_cardiac. For automated CMR image analysis, we used python v3.6 and tensorflow v1.9.0.

For manuscripts utilizing custom algorithms or software that are central to the research but not yet described in published literature, software must be made available to editors and reviewers. We strongly encourage code deposition in a community repository (e.g. GitHub). See the Nature Portfolio guidelines for submitting code & software for further information.

Data

Policy information about availability of data

All manuscripts must include a data availability statement. This statement should provide the following information, where applicable:

- Accession codes, unique identifiers, or web links for publicly available datasets
- A description of any restrictions on data availability
- For clinical datasets or third party data, please ensure that the statement adheres to our policy

The full genome-wide association summary statistics generated in this study have been deposited in the GWAS Catalog under accession codes GCST90797570–GCST90797613 [https://www.ebi.ac.uk/gwas/downloads/summary-statistics]. These summary statistics are fully available for download and use. The raw individual-level genotype and cardiac MRI data from the UK Biobank are protected and are not publicly available due to data privacy laws; access can be obtained through application to the UK Biobank Access Management System (https://www.ukbiobank.ac.uk/enable-your-research/apply-for-access), subject to UK Biobank approval. The data generated in this study for all figures and source data are provided in the Supplementary Information/Source Data file. Source data are provided with this paper. The HF GWAS performed by Levin et al. and used in our colocalization analysis is available in the GWAS Catalog (accession ID: GCST90162626 [https://www.ebi.ac.uk/gwas/studies/GCST90162626]).

Research involving human participants, their data, or biological material

Policy information about studies with human participants or human data. See also policy information about sex, gender (identity/presentation), and sexual orientation and race, ethnicity and racism.

Reporting on sex and gender	Self-reported sex at the time of recruitment from the UK Biobank was an adjusted covariate in the genome-wide association study. Sex was included as a covariate to account for well-documented sexual dimorphism in cardiac structure and function, specifically regarding ventricular volumes and myocardial mass. Gender identity (a social construct) was not collected by the UK Biobank at the time of recruitment, and therefore no analyses were performed regarding gender-specific social or cultural factors. Findings are reported for the total population with sex-adjustment; sex-stratified analyses were not the primary focus of this discovery GWAS. Overall this study included 27,277 males and 29,232 females.
Reporting on race, ethnicity, or other socially relevant groupings	We used genetic principal component analysis to ascertain ancestry and selected European ancestry for our GWAS.
Population characteristics	The total 56,609 individuals studied were of European ancestry, all population characteristics have been outlined in supplementary table 1.
Recruitment	The UK Biobank is a prospective cohort study that recruited ~500,000 participants aged between 49-60 years who were registered with the UK National Health Service and living <25 miles from any of the 22 enrollment centres. Informed consent was obtained from all participants in accordance with the UK Biobank ethics framework (https://www.ukbiobank.ac.uk/learn-more-about-uk-biobank/governance/ethics-advisory-committee). Participants with cardiovascular magnetic resonance imaging data were identified. Potential biases with this cohort lie in limited sample size with annotated cardiovascular magnetic resonance images and the cohort being predominantly European.
Ethics oversight	The ethics approval for the UK Biobank was provided by the National Health Service National Research Ethics Service (June 17, 2011 [reference 11/NW/0382] and was extended on May 10, 2016 [reference 16/NW/0274]).

Note that full information on the approval of the study protocol must also be provided in the manuscript.

Field-specific reporting

Please select the one below that is the best fit for your research. If you are not sure, read the appropriate sections before making your selection.

[x] Life sciences [ ] Behavioural & social sciences [ ] Ecological, evolutionary & environmental sciences

For a reference copy of the document with all sections, see nature.com/documents/nr-reporting-summary-flat.pdf

Life sciences study design

All studies must disclose on these points even when the disclosure is negative.

Sample size	Sample size was determined by the availability of individuals with cardiovascular magnetic resonance imaging and who have had their images annotated with the phenotypes investigated. Based on the sample sizes used in prior GWASs for similar traits, our sample size is adequate (>80% power) to discover novel loci at a pre-specified P threshold of 5x10-8.
Data exclusions	Participants with poor CMR image quality or poor genotype data quality were excluded.
Replication	Associated loci were compared against previous genome-wide association studies across cardiovascular magnetic resonance imaging phenotypes to discover replicated/validate loci. Due to a small sample size of available participants with cardiovascular magnetic resonance imaging data, the GWAS analysis was not replicated.
Randomization	Given the observational nature of this study and GWAS design, randomization was not applicable.

Blinding	CMR image analysts were blinded to participants' genotype, demographic and clinical characteristics. Blinding was not relevant to the downstream genome-wide association study analysis, which provides data per genetic variant, no longer at the participant level.

Reporting for specific materials, systems and methods

We require information from authors about some types of materials, experimental systems and methods used in many studies. Here, indicate whether each material, system or method listed is relevant to your study. If you are not sure if a list item applies to your research, read the appropriate section before selecting a response.

Materials & experimental systems

n/a	Involved in the study	
☒	☐	Antibodies
☒	☐	Eukaryotic cell lines
☒	☐	Palaeontology and archaeology
☒	☐	Animals and other organisms
☒	☐	Clinical data
☒	☐	Dual use research of concern
☒	☐	Plants

Methods

n/a	Involved in the study	
☒	☐	ChIP-seq
☒	☐	Flow cytometry
☒	☐	MRI-based neuroimaging

Plants

Seed stocks	*Report on the source of all seed stocks or other plant material used. If applicable, state the seed stock centre and catalogue number. If plant specimens were collected from the field, describe the collection location, date and sampling procedures.*
Novel plant genotypes	*Describe the methods by which all novel plant genotypes were produced. This includes those generated by transgenic approaches, gene editing, chemical/radiation-based mutagenesis and hybridization. For transgenic lines, describe the transformation method, the number of independent lines analyzed and the generation upon which experiments were performed. For gene-edited lines, describe the editor used, the endogenous sequence targeted for editing, the targeting guide RNA sequence (if applicable) and how the editor was applied.*
Authentication	*Describe any authentication procedures for each seed stock used or novel genotype generated. Describe any experiments used to assess the effect of a mutation and, where applicable, how potential secondary effects (e.g. second site T-DNA insertions, mosiacism, off-target gene editing) were examined.*
